# Benzo-ring modification on Malaria Box hit MMV008138: effects on antimalarial potency and microsomal stability

**DOI:** 10.1039/d5md00439j

**Published:** 2025-08-15

**Authors:** Maryam Ghavami, Haibo Li, Lixuan Liu, Joshua H. Butler, Sha Ding, Grant J. Butschek, Reagan S. Haney, R. McAlister Council-Troche, R. Justin Grams, Emilio F. Merino, Jennifer M. Davis, Maxim Totrov, Maria B. Cassera, Paul R. Carlier

**Affiliations:** a Department of Chemistry and Virginia Tech Center for Drug Discovery, Virginia Tech 1040 Drillfield Drive Blacksburg VA 24061 USA; b Department of Biochemistry and Molecular Biology and Center for Tropical and Emerging Global Diseases, University of Georgia 120 E. Green St. Athens GA 30602 USA; c Department of Biomedical Sciences & Pathobiology, Virginia-Maryland College of Veterinary Medicine Blacksburg VA 24061 USA; d Molsoft LLC 11999 Sorrento Valley Road San Diego CA 92121 USA; e Department of Pharmaceutical Sciences, University of Illinois at Chicago 833 S. Wood St Chicago IL 60612 USA pcarlier@uic.edu

## Abstract

Tetrahydro-β-carboline 1 (MMV008138) controls growth of asexual blood-stage *Plasmodium falciparum* by inhibiting IspD, an enzyme in the MEP pathway for synthesis of a critical metabolite, isopentenyl pyrophosphate (IPP). We have previously investigated the structure activity relationship (SAR) of three of its four rings (B, C, and D). In this report we investigate the SAR of the benzo- (*i.e.* A-ring) of 1, with the goal of increasing its *in vitro* antimalarial potency and metabolic stability. As in our previous studies of the B- and C-ring substitution, extreme sensitivity to substitution was also seen in the benzo-ring. In total, 19 benzo-ring substitution variants of 1 were prepared. When tested against multidrug-resistant (Dd2 strain) *P. falciparum*, only three derivatives (20a, c, d) possessed asexual blood stage (ABS) activity with EC_50_ values within 3-fold of the parent. As hoped, one analog (20c) showed a marked improvement in microsomal stability. However, this improvement unfortunately did not improve plasma exposure relative to 1, and did not lead to oral efficacy in a mouse model of malaria.

## Abstract 2

7-fluoro analog 20c showed greatly improved *in vitro* metabolic stability relative to 1, with only a 50% reduction in antimalarial potency.

## Graphical abstract

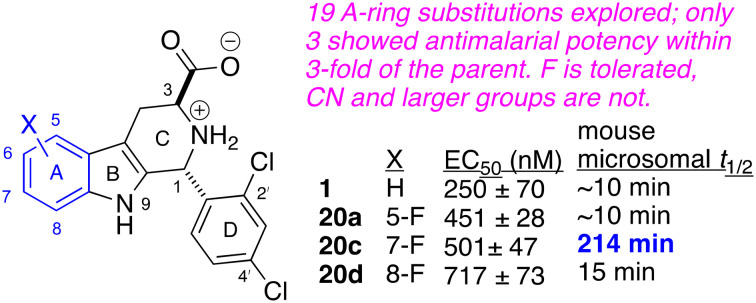

## 1. Introduction

**Fig. 1:**
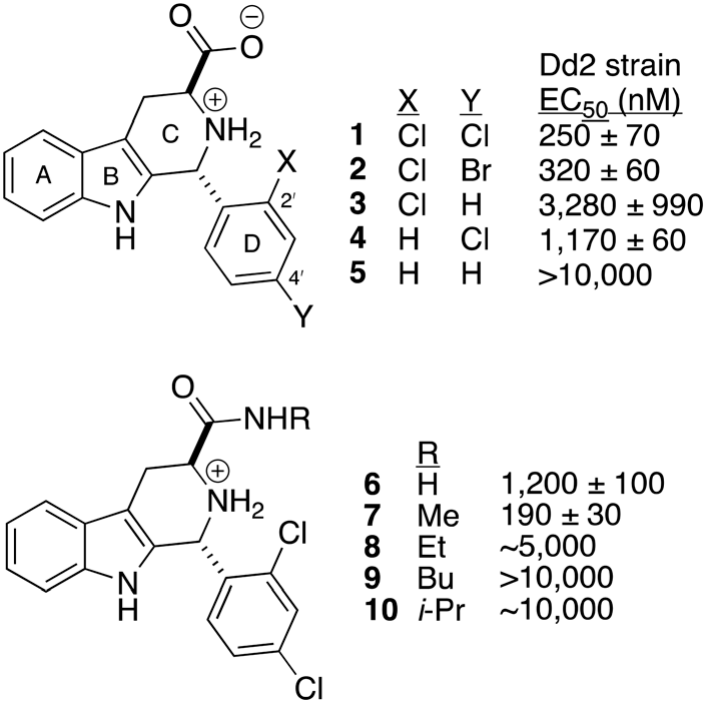
Structures of tetrahydro-β-carboline antimalarial MMV008138 (1), D-ring analogs 2–5, and amide derivatives 6–10. Reported growth inhibition values (EC_50_) are derived from 72 h exposure of multidrug-resistant Dd2 strain *Plasmodium falciparum*.

Malaria remains a serious threat in the developing world, killing more than 590 000 people in 2023, most of whom were children under 5 years of age.^1^ Furthermore, the annual case incidence rate of 263 million persons remains largely unchanged from 2015. This unfortunate stall in malaria reduction is due to several factors, but rapidly emerging resistance to all current antimalarials is a key contributor. Thus, the discovery of antimalarials with new mechanisms of action is essential. We have invested considerable effort to study analogs of MMV008138 (1, Fig. 1), one of the original 400 compounds of the Malaria Box.^2^

Compound 1 attracted our interest because it controls growth of *Plasmodium falciparum* by inhibiting the methylerythritol phosphate (MEP) pathway for isoprenoid precursor biosynthesis.^3^ Since this pathway is essential for *P. falciparum*,^4,5^ and absent in human,^6^ it represents a very promising target for therapeutic development. Studies in our lab and those of others established that *in vitro* antimalarial activity of 1 resides only in the depicted (1*R*,3*S*)-stereoisomer,^7,8^ and results from inhibition of the third enzyme of the MEP pathway (IspD).^7,9,10^ In addition, 2′,4′-halogen di-substitution of the D-ring was found to be critical for potency (*cf.*1, 2*vs.*3–5),^8,10^ and the 3-carboxy group is essential and can only be replaced by a methyl amide (*cf.*7*vs.*6, 8–10, Fig. 1).^8,10^

**Fig. 2:**
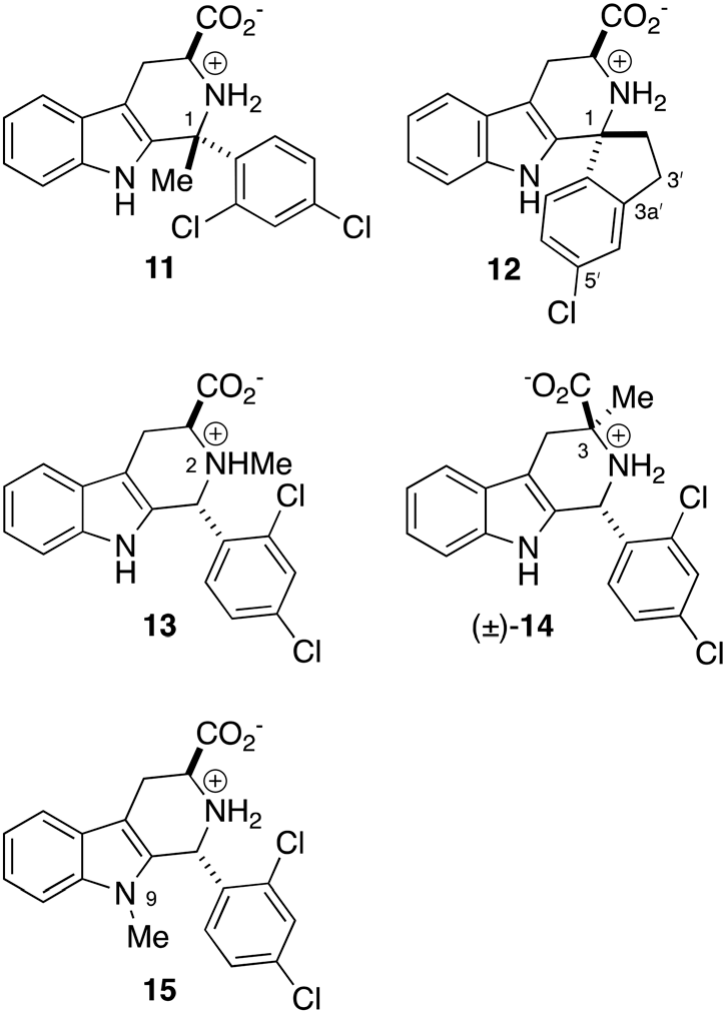
Inactive analogs of 1 (EC_50_ ≥ 8000 nM) featuring methyl and spiro-substitution at C1 (11, 12), and methyl substitution at N2, C3, and N9 (13–15).

As a recent review highlights, IspD is a compelling drug target not only for malaria, but also for the development of new antibacterials and herbicides.^11^ However to date, no IspD-targeting antimalarial has shown efficacy in an animal model of infection, including benzoisothiazolones,^12^ diarylureas,^13^ and as we previously reported,^14^ tetrahydro-β-carboline 1. To remedy this deficiency of 1, methyl- and spiro-substitution of C1 (*i.e.*11, 12), and methyl substitution at N2, C3, or N9 (*i.e.*13–15, Fig. 2) were explored to exert conformational bias, as a means to improve target engagement.^15^ However, all these changes abrogated *P. falciparum* growth inhibition potency (EC_50_ > 8000 nM).

In this report we take a new direction to discover improved analogs of 1, reporting the synthesis and bioassay of 19 compounds that vary in substitution of the benzo-ring (*i.e.* A-ring, Fig. 1). Surprisingly, even the smallest substituent explored (F) adversely effected growth inhibition potency. However, judicious placement of a fluorine at C7 greatly improved metabolic stability, while increasing growth inhibition EC_50_ 2-fold. Lastly, we describe pharmacokinetic and efficacy studies of 7-fluoro analog 20c, and compare them to 1.

## 2. Results and discussion

### 2.1 Design, synthesis, and *in vitro* antimalarial potency

The motivation to explore substitution of the benzo ring to improve *in vitro* antimalarial potency arose from the two factors; it was the last remaining unexplored area for structural modification of 1, and we suspected that benzo-ring substitution might improve plasma exposure. In mouse liver microsomes 1 was found to have *t*_1/2_ ∼10 min (Table 3 below). Although metabolite ID studies were not undertaken, we suspected that Cyp450-mediated oxidation of the benzo-ring of the indole was responsible for the rapid loss of parent. Thus, we hoped that appropriate substitution on this ring might serve two functions: slow metabolism, and improve *in vitro* potency by improving ligand-protein complementarity.

For convenience, initial exploration of benzo-ring variants of 1 focused on racemic compounds. Since we have published syntheses of D-ring,^8,10^ and B/C-ring variants^15^ of 1 previously, here we provide only a brief outline of the synthesis of these compounds; full details are provided in the SI. Commercial substituted indoles were first converted to the corresponding racemic substituted tryptophan methyl esters.^16^ These in turn were reacted with 2,4-dichlorobenzaldehyde, and the *trans*-Pictet–Spengler adducts were obtained by column chromatography; stereochemistry was confirmed by ^1^H NMR.^17^ Ester hydrolysis was achieved using a catch-and-release protocol,^18^ giving the desired amino acid zwitterions. *In vitro* ABS *P. falciparum* (Dd2 strain) growth inhibition potencies (EC_50_ values) for the racemic samples are shown in Table 1.

**Table 1:** ABS *P. falciparum* (Dd2 strain) growth inhibition potencies of 1 and racemic benzo-ring substitution variants^a^

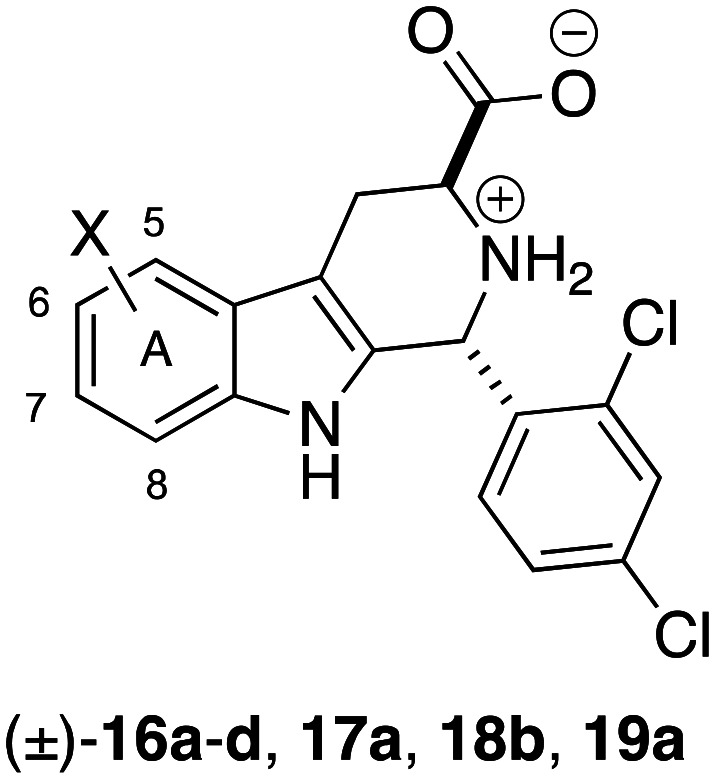		
Compound	X	Dd2 strain *P. falciparum* growth inhibition EC_50_ (nM)
1	H	250 ± 70 nM^b^
(±)-16a	5-Me	>10 000 nM
(±)-16b	6-Me	>10 000 nM
(±)-16c	7-Me	>10 000 nM
(±)-16d	8-Me	>10 000 nM
(±)-17a	5-CF_3_	>10 000 nM
(±)-17c	7-CF_3_	1250–2500 nM
(±)-18b	6-Br	>10 000 nM
(±)-19a	5-Cl	>10 000 nM

a Growth inhibition (EC_50_ values) of ABS was determined using SYBR Green I assay at 72 h endpoint. *P. falciparum* Dd2 strain is multi-drug resistant.

b Previously reported.^8^

As can be seen in Table 1, all of the Me-, CF_3_-, Br-, and Cl-substitutions explored resulted in a >40-fold loss of growth inhibition potency, relative to 1. In particular, placement of single methyl group at any position of the benzo ring is not tolerated. This remarkable sensitivity to substitution on the A-ring of 1 parallels what was seen for substitution on the B- and C-rings (Fig. 2).^15^

**Scheme 1:**
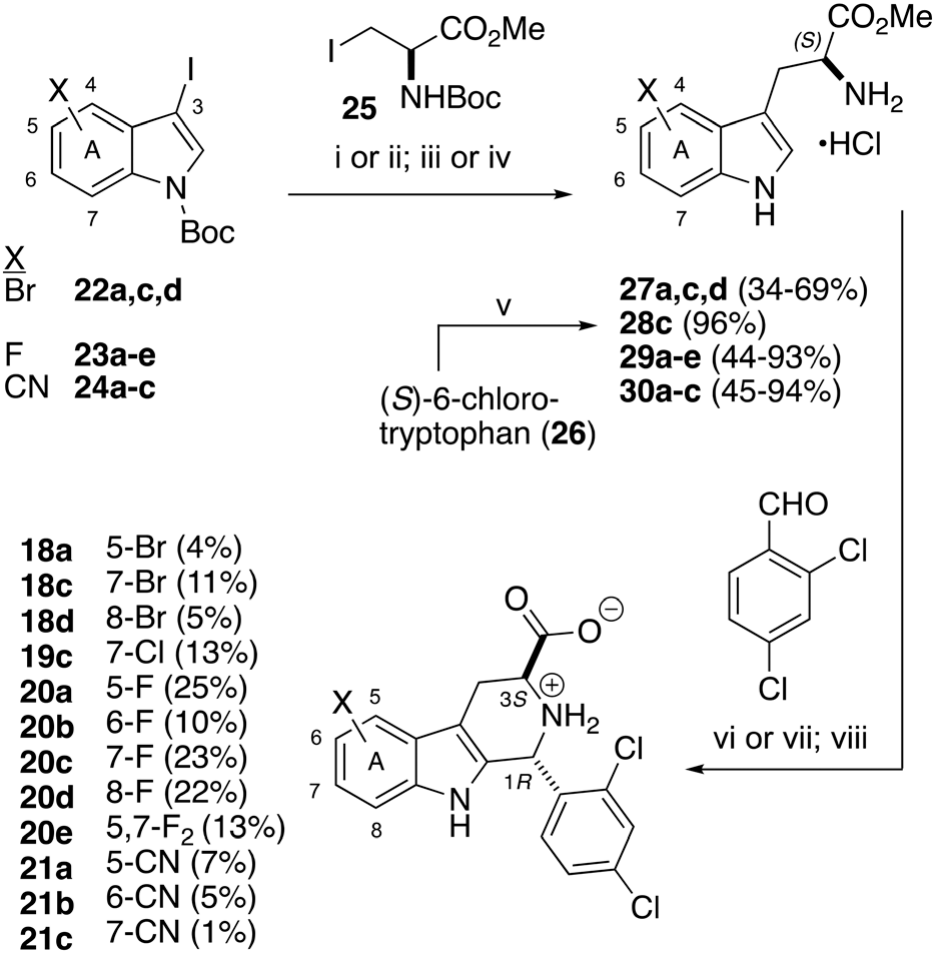
Synthesis of enantiopure A-ring variants of 1. i) NiCl_2_ (10 mol%), BPhen (10 mol%), Mn (3 equiv.), 25 (1.2 equiv.), NMP. ii) Zn (2.9 equiv.), I_2_ (0.1 equiv.), 25 (1.3 equiv.), DMF; add iodoindole, Pd_2_dba_3_ (2 mol%), SPhos (4 mol%). iii) HCl in MeOH. iv) HCl in EtOAc. v) SOCl_2_, MeOH. vi) 4 Å MS, CH_2_Cl_2_; TFA; isolate *trans*-diastereomer. vii) Ti(Oi-Pr)_4_, TFAA, TFA, 70 °C; MeOH, NaOH (aq); isolate *trans*-diastereomer. viii) Amberlyst hydroxide, THF/MeOH/H_2_O, 16 h; AcOH (aq).

We thus turned our attention to the synthesis of analogs bearing smaller substituents (F, CN). These compounds were prepared in enantiopure form, maintaining the desired (1*R*,3*S*)-configuration of 1. For the sake of completeness, we also continued our exploration of Br- and Cl-substitution. To synthesize these compounds, substituted *N*-Boc-3-iodoindoles 22a, c, d, 23a–e and 24a–c (prepared in two steps from the commercial indoles, see SI) were converted to the requisite substituted (*S*)-tryptophan methyl esters 27a, c, d, 29a–e, and 30a–c (Scheme 1). This transformation was achieved by either Ni-catalyzed reductive cross-coupling^19^ or Negishi coupling^14,20^ of the corresponding *N*-Boc-3-iodoindole and (*R*)-*N*-Boc-iodoalanine methyl ester 25. Both cross-coupling methods are known to proceed with retention,^19–21^ affording enantiopure products. (*S*)-6-Chlorotryptophan methyl ester hydrochloride 28c was prepared from the commercial amino acid 26. Note that Boc-deprotection to afford cyano-substituted tryptophan methyl esters 30a–c had to be performed with HCl/EtOAc rather than HCl/MeOH, to avoid Pinner reaction of the cyano group.

These (*S*)-tryptophan methyl esters were then subjected to Pictet–Spengler reaction with 2,4-dichlorobenzaldehyde^8,10^ and the desired *tran*s-esters were isolated and identified by analysis of ^1^H-^1^H coupling constants.^17^ In the case of cyano-substituted tryptophans 30a–b, the standard Pictet–Spengler protocol did not produce any of the desired tetrahydro-β-carbolines, apparently due to the strongly electron-withdrawing effect of the CN group. We thus applied Horiguchi's Ti(Oi-Pr)_4_-mediated protocol,^22^ which we had previously reported for the synthesis of 11 and 12.^15^ Catch-and-release hydrolysis using Amberlyst hydroxide^18^ gave the desired (1*R*,3*S*)-configured A-ring variants of 1 (Scheme 1). Note that the yields of desired *trans*-diastereomers in Pictet–Spengler reactions rarely exceeds 25%. The very low two-step yields reported for several of the compounds in Scheme 1 reflect difficulties in chromatographic isolation of the desired *trans*-ester intermediate, and in the case of cyano-substituted tryptophans 30a–c, poor conversion in the Pictet–Spengler reaction, as mentioned above.

As can be seen in Table 2, in the enantiopure series, bromo-substitution at C5, C7, and C8 adversely affected growth inhibition potency (18a, 18c, 18d), as it had at in the racemic series at C6 ((±)-18b, Table 1). The enantiopure 7-chloro analog 19c suffered only a 5-fold loss in growth inhibition potency, less than the 17-fold potency loss of 7-bromo analog 18c, relative to 1. Moving to the fluorinated series 20a–e, sub-micromolar growth inhibition potency was finally achieved.

**Table 2:** ABS *P. falciparum* (Dd2 strain) growth inhibition potencies of 1 and enantiopure benzo-ring substitution variants^a^

Compound	X	Dd2 strain *P. falciparum* growth inhibition EC_50_ (nM)	% recovery^b^ (200 μM IPP)
1	H	250 ± 70 nM^c^	100%@2.5 μM^c^
18a	5-Br	>10 000	nd
18c	7-Br	4300 ± 600	nd
18d	8-Br	>10 000	nd
19c	7-Cl	1300 ± 100	nd
20a	5-F	451 ± 28	100%@2.5 μM
20b	6-F	1250–2500	nd
20c	7-F	501 ± 47	100%@2.5 μM
20d	8-F	717 ± 73	100%@10 μM
20e	5,7-F_2_	964 ± 63	80%@10 μM
21a	5-CN	>10 000	nd
21b	6-CN	>10 000	nd
21c	7-CN	>20 000	nd

a Growth inhibition (EC_50_ values) of ABS *P. falciparum* was determined using SYBR Green I assay at 72 h endpoint. Values represent average ± S.E.M from at least two biological replicates (with two technical replicates). *P. falciparum* Dd2 strain is multi-drug resistant. For compounds that did not achieve 100% growth inhibition at 10 000 nM, EC_50_ values are reported as “>X” where X is the highest concentration at which no inhibition was observed.

b Recovery of parasite growth activity seen in the presence of 200 μM IPP, at the indicated concentration of drug.

c Previously reported.^8^

In particular, 5-fluoro (20a), 7-fluoro (20c), 8-fluoro (20d), and 5,7-difluoro (20e) analogs met this criterion. The most potent example, 20c was roughly half as potent as 1. All demonstrated metabolic rescue upon co-application of IPP, demonstrating that their antimalarial action (like that of 1) is due to inhibition of the MEP pathway.^3,4^ Interestingly, none of the 3-cyano substituted analogs explored inhibited *P. falciparum* growth at the highest concentrations tested (10 000–20 000 nM). Both fluoro- and cyano- are considered small substituents: their “thin-ness” is reflected in very small A-values (0.15 and 0.17 respectively; *cf.* 1.7 for methyl, all in kcal mol^−1^).^23^ The decisive difference between F- and cyano-substitution likely stems from length. Whereas the C–F bond length in fluorobenzene is 1.34 Å,^24^ the benzo-C-CN distance in benzonitrile is 1.269 Å longer (2.609 Å).^25^ Apparently a substituent of this length cannot be accommodated at the 5-, 6-, or 7-positions.

### 2.2 *In vitro* metabolic stability, *in vivo* pharmacokinetics and *in vivo* antimalarial efficacy

To test our hypothesis that benzo-ring substitution of 1 might reduce the rate of liver metabolism, we tested the three most potent fluorinated analogs (20a, 20c, 20d) against 1 in mouse liver microsomes (Table 3). As can be seen, only 20c, featuring a 7-fluoro substituent evidenced a lower rate of metabolism in liver microsomes, with *t*_1/2_ of 214 min. This promising increased oxidative stability prompted pharmacokinetic analysis in mice (40 mg kg^−1^ po, 10 mg kg^−1^ IV). Both 1 and 20c are absorbed very quickly, with *t*_max_ at 0.25 and 0.5 h respectively (Table 3).

**Table 3:** Mouse microsomal stability and *in vivo* mouse pharmacokinetic data of 1 and fluorinated analogs 20a, c, d

Parameter	1	20a	20c	20d
Microsomal *t*_1/2_ (min)	∼10^a^	∼10^a^	214	16
*t*_max_ (h)^b^	0.25	nd	0.5	nd
*C*_max_ (μM)^b^	46.1	nd	19.9	nd
AUC_0-inf_ (h μM)^b^	88	nd	114	nd
CL_obs_ (mL min^−1^ kg^−1^)	12.1	nd	8.85	nd
*t*_1/2_ (h)^c^	8.5	nd	5.2	nd
% *F*	57	nd	58	nd
*V*_d_ (L)	3.5	nd	3.8	nd

a Approximate, see SI.

b Oral dosing 40 mg kg^−1^, first oral time point is 0.25 h.

c Oral, 40 mg kg^−1^, reflects elimination phase only (4–24 h).

But to our surprise, the improved microsomal stability of 20c relative to 1 (*t*_1/2_ of 214 *vs.* ∼10 min) did not manifest in greatly improved plasma exposure. The oral AUC_0-inf_ value of 20c is only 30% higher than that of 1, and the IV Cl_obs_ value of 20c is correspondingly ∼30% lower than that of 1. Contrary to expectation, the elimination phase half-life of 20c (5.2 h) is somewhat shorter than that of 1 (8.5 h). Furthermore, no significant differences were seen between 1 and 20c in terms of oral bioavailability and volume of distribution (Table 3). It is possible that clearance in the elimination phase of 1 and 20c is not driven by Cyp450 oxidation, but rather by phase II processes, such as glucuronidation of the carboxyl group. Nevertheless, we evaluated 20c for *in vivo* efficacy in the same *P. berghei* mouse model used for 1. Unfortunately, as for both oral and IV dosing of 1 (Fig. S1–S3, Tables S1 and S2), no reduction in parasitemia was seen following oral dosing of 20c (5 × 40 mg per kg per day, Fig. S6, Table S10). Apparently, the marginal increase in the AUC_0-inf_ value of 20c is more than offset by its lower *in vitro* antimalarial potency relative to 1. It appears that in order to realize *in vivo* efficacy in this series, substantial improvements in plasma exposure must be achieved.

## 3. Conclusion

To attempt improvement of the antimalarial properties of 1, we explored 19 benzo-ring substitution variants. Unexpectedly, introduction of Cl, Br, Me, CF_3_, and CN groups on the benzo-ring greatly reduced *in vitro* growth inhibition potency against asexual blood stages of *P. falciparum* (Tables 1 and 2). Only 5-fluoro (20a), and 7-fluoro (20c) substitution gave EC_50_ values within 2-fold of 1 (Table 2). As hoped, 7-fluoro variant 20c had greatly improved mouse microsomal stability (Table 3, *t*_1/2_ = 214 min *vs.* ∼10 min for 1). However, this improvement did not improve plasma exposure in mice relative to 1 following oral dosing at 40 mg kg^−1^ (Table 3), and 20c was not efficacious in a *P. berghei* mouse infection model (40 mg kg^−1^ × 5 d). Thus, oral efficacy in the tetrahydro-β-carboline class of IspD inhibitors remains elusive.

Looking forward, *in vitro P. falciparum* growth inhibition potency for this pharmacophore undoubtedly requires significant improvement. We see two significant questions to address here. First, is the extremely tight growth inhibition SAR demonstrated in this work and earlier studies driven solely by target engagement, or does access to the apicoplast, (where *Pf*IspD is located) play a significant role? Our earlier study of the *Pf*IspD inhibition SAR of D-ring substitution and carboxyl analogs of 1 demonstrated a good correspondence between *P. falciparum* growth inhibition EC_50_ values and *Pf*IspD IC_50_ values. However, more comparative work in this area is certainly merited, and an understanding of factors that improve IspD inhibitor accumulation in the apicoplast would be greatly beneficial.

Second, since no X-ray structures of *Plasmodium* spp. IspD have been reported to date, could docking studies with homology models be useful to develop more potent *Pf*IspD inhibitors? Numerous bacterial and plant IspD X-ray structures are available, and one earlier study docked 1 in the CTP-binding site of a *Pf*IspD homology model derived from *Escherichia coli* IspD.^9^ However allosteric CTP-competitive inhibition of *Arabidopsis thaliana* IspD has been demonstrated crystallographically for the azolopyrimidines,^26^ pseudilins,^27^ and phenylisoxazoles.^28^ The case for allosteric inhibition of *Pf*IspD by 1 is also supported by the observation that one resistance locus (E688Q)^9^ is in the C-terminal domain, far from the CTP-binding site. Thus, we are not confident that homology modeling would lead to accurate binding predictions at this time. We are currently focusing our efforts on crystallizing *Pf*IspD in the presence of inhibitors like 1 and 20c.

Lastly, the usefulness of the *P. berghei*-infection *in vivo* mouse model to assess efficacy of *P. falciparum* IspD inhibitors remains an open question. Other investigators have shown that *P. vivax* IspD is less sensitive than *P. falciparum* IspD to inhibition by 1 (IC_50_ values of 310 and 47 nM, respectively).^9^ Therefore, *P. berghei* IspD may also be much less sensitive than the *P. falciparum* IspD to inhibition by 1 and 20c, which would explain the lack of *in vivo* efficacy. This possibility is under investigation, and results will be communicated in due course.

## 4. Experimental

### 4.1 Synthesis of antimalarial compounds

New compounds (19 in total) were prepared according to the methods described in Scheme 1 and accompanying text. Detailed synthetic procedures, purification protocols, and full tabulation of analytical data are provided in the SI. Nuclear magnetic resonance (NMR) spectroscopy (^1^H, ^13^C, ^19^F), and high resolution mass spectrometry (HRMS-ESI) confirmed the proposed structure in each case. NMR spectra were obtained at ^1^H-resonant frequencies of 400 and 500 MHz; all tested compounds were >95% pure (see SI).

### 4.2 *P. falciparum* culture for asexual blood stage activity

*P. falciparum* strains Dd2 (MRA-150, resistant to chloroquine, pyrimethamine and mefloquine) and 3D7 (MRA-102, drug-sensitive) were obtained from MR4 Malaria Reagent Repository (ATCC, Manassas, VA), a part of the BEI Resources Repository (NIAID, NIH). Parasites were maintained in O-positive human erythrocytes (Grifols, Memphis, TN, USA) at 5% hematocrit in RPMI 1640 medium supplemented with 5 g L^−1^ Albumax I (Gibco Life Technologies), 2 g L^−1^ glucose (Sigma-Aldrich), 2.3 g L^−1^ sodium bicarbonate (Sigma-Aldrich), 370 μM hypoxanthine (Sigma-Aldrich), 25 mM HEPES, and 20 mg L^−1^ gentamicin (Gibco Life Technologies). The parasites were kept at 37 °C under reduced-oxygen conditions (5% CO_2_, 5% O_2_, and 90% N_2_).

### 4.3 Growth inhibition and metabolic rescue by isopentenyl diphosphate (IPP) supplementation assays

The effect of antimalarial candidates was evaluated against asexual blood stages of *P. falciparum* parasites using the SYBR green I assay as described previously.^8^ Antimalarial activity was first evaluated against asexual parasites using four-point dilutions ranging from 10 μM to 1.25 μM. For active compounds, the half maximal effective concentration (EC_50_) was determined using ten-point dilutions at concentrations ranging from 5–10 μM to 0.005–0.01 μM in constant 0.1% DMSO (vehicle). MMV008138 (1) was used as control. The percentage of growth was normalized to that of untreated control parasites in the presence of 0.1% DMSO. Background determinations were made using uninfected erythrocytes. Two or more independent experiments in duplicate were performed. The EC_50_ values were calculated with GraphPad Prism 9 (GraphPad Software Ltd.) using nonlinear regression curve fitting with variable slope (four parameters) and represent the average and their standard error of the mean (S.E.M.). To assess whether 20a–e specifically targeted the apicoplast, the recovery of growth in the presence of inhibitor and isopentenyl diphosphate (IPP) was performed as described previously.^8^ Briefly, parasites were grown in the presence or absence of 200 μM IPP along with a serial dilution (10 μM to 0.01 μM) or single concentration (10 or 20 μM) of drug. MMV008138 (EC_50_ = 170 ± 10 nM) was used as control at a single concentration (Fig. S22). All conditions were set in 96-well half area plates using highly synchronous ring-stage parasite cultures (100 μL per well at 1% hematocrit and 1% parasitemia) and incubated for 72 h under normal culture conditions. Parasite growth was measured by the SYBR green I assay.

## List of abbreviations

ABS: Asexual blood stage
SAR: Structure–activity relationships
EC_50_: Half-maximal effective concentration

## Author contributions

MT, MBC, and PRC conceptualized the research project. MG, HL, LL, SD, and RJG carried out the chemical synthesis and/or analytical characterization, and PRC directed and oversaw these activities. JHB, GJB, RSH, and EFM carried out the *in vitro* antimalarial evaluations, and MBC directed and oversaw these activities. EFM carried out the *in vitro* metabolic stability. RMCT carried out the *in vivo* pharmacokinetic study of 20c, and JMD oversaw and directed this activity. Data analysis and interpretation were carried out by MG, MT, JMD, MBC, and PRC. All authors contributed to the writing their respective parts of the manuscript; MBC and PRC combined these contributions and produced the final manuscript. All authors approved the manuscript prior to submission.

## Conflicts of interest

The authors declare no conflicts of interest.

## Supplementary Material

MD-OLF-D5MD00439J-s001

## Data Availability

Supplementary information is available: The data supporting this article have been included as part of the SI, which includes 1) synthetic procedures, tabulations of analytical data, and NMR spectra of all tested compounds; 2) full data and procedures for mouse microsomal stability (in-house and CRO); 3) *in vivo* PK (in-house and CRO) and 4) *in vivo* mouse efficacy studies (CRO). See DOI: https://doi.org/10.1039/D5MD00439J.
