# Supplementary material for: Benzo-ring modification on Malaria Box hit MMV008138: effects on antimalarial potency and microsomal stability
Source: RSC Med Chem. 2025 Aug 15;16(10):5052–8. doi: 10.1039/d5md00439j (PMC12378621; doi:10.1039/d5md00439j)
Supplement: MD-016-D5MD00439J-s001 [file MD-016-D5MD00439J-s001.pdf]

Supplementary Information (SI) for RSC Medicinal Chemistry.
This journal is © The Royal Society of Chemistry 2025

Electronic Supporting information for the manuscript entitled

Benzo-ring modification on Malaria Box hit MMV008138: effects on antimalarial potency and microsomal stability

Maryam Ghavami[a], Haibo Li[a], Lixuan Liu[a], Joshua H. Butler[b], Sha Ding[a], Grant J. Butschek[b], Reagan S. Haney[b], McAlister Council-Troche[c], R. Justin Grams[a], Emilio F. Merino[b], Jennifer Davis[c], Maxim Totrov[d], Maria Belen Cassera[b], Paul R. Carlier[a,e,*]

*corresponding author: pcarlier@uic.edu

[a]Department of Chemistry and Virginia Tech Center for Drug Discovery, Virginia Tech, 1040 Drillfield Drive, Blacksburg, VA 24061. [b]Department of Biochemistry and Molecular Biology and Center for Tropical and Emerging Global Diseases, University of Georgia, 120 E. Green St., Athens, GA 30602. [c]Department of Biomedical Sciences & Pathobiology, Virginia-Maryland College of Veterinary Medicine, Blacksburg, VA, 24061. [d]Molsoft LLC, 11999 Sorrento Valley Road, San Diego CA, 92121. [e]Department of Pharmaceutical Sciences, University of Illinois at Chicago, 833 S. Wood St, Chicago, IL 60612.

Table of Contents

Section	Description	Page
1.	Synthesis and analytical characterization of tested compounds	S1
2.	*In vivo* efficacy studies of **1** in *P. berghei*-infected mice (NYU Antiinfectives Core Facility)	S34
	A. Oral administration (Figure S1, Table S1)	
	B. IV administration (Figures S2-S3, Table S2)	
3.	PK studies of PRC1080 (Pharmaron, Tables S3-S4, Figure S4)	S36
4.	Mouse microsomal stability of **1** (Pharmaron, Table S5.)	S38
5.	Mouse microsomal stability of benzo-ring fluorinated analogs **20a**, **20c**, **20d** (Tables S6, S7)	S39
6.	PK studies of **20c** (Virginia Tech, Tables S8, S9, Figure S5)	S40
7.	*In vivo* efficacy studies of **20c** in *P. berghei*-infected mice (NYU Antiinfectives Core Facility, Table S10, Figure S6)	S42
8.	References	S44
9.	NMR Spectra of tested compounds	S45

1. Synthesis and analytical characterization of tested compounds

Nuclear magnetic resonance (NMR) spectroscopy (^{1}H, ^{13}C, ^{19}F), and high resolution mass spectrometry (HRMS-ESI) confirmed the proposed structure in each case. NMR spectra were obtained at ^{1}H-resonant frequencies of 400 and 500 MHz. ^{19}F NMR spectra were ^{1}H-coupled. All tested compounds were > 95% pure.

General Methods A1 &A2: Synthesis of racemic *N*-acetyl tryptophans

General Method A1: *Adapted from Konda-Yamada et al*.[1] To a round-bottom flask was added the required substituted indole (5.13 mmol) and *dl*-Serine (10.3 mmol). The flask was purged with nitrogen and AcOH (12 mL) and Ac_2O (4 mL) were added to this mixture. The mixture was allowed to stir at room temperature under nitrogen for 10 minutes and then heated to 75 °C for 2-5 h. The mixture was then cooled down and diluted with Et_2O (100 mL). 30% NaOH (40 mL) was added until the pH was basic. The water layer was further washed with diethyl ether (3×100 mL). The mixed organic extracts were further extracted with 1 M NaOH (2×30 mL). This aqueous basic extract was mixed with the previous basic extract mixture, a small amount of $Na_2S_2O_4$ were added and the mixture was cooled down in an ice bath. Subsequently, conc. HCl, concentrated were added until the pH was acidic. The organic layer was extracted with EtOAc (4×150 mL). The combined organic extracts were washed with brine (2×20 mL), dried over sodium sulfate and concentrated *in vacuo*. The resulting residue was purified by a short silica flash chromatography [(90:10): DCM: [MeOH:AcOH (1-2%)]] to afford the substituted *N*-acetyl tryptophan (est. purity 85-90%), which was taken directly to the next step without further purification.

General Method A2: *Adapted from Yokoyama et al.*[2] To a round bottom flask was added *dl*-Serine (20.75 mmol). The flask was purged with nitrogen and AcOH (34 mL) and Ac_2O (5.6 mL) were added to this mixture. The mixture was allowed to stir at room temperature under nitrogen for 10 minutes and then heated to 45 °C for 5 h. The requisite substituted indole (10.0 mmol) was added to the clear solution and the mixture was heated to 80 °C for 1.5-3 h. The mixture was then cooled down and diluted with Et_2O (100 mL). 30% NaOH (40 ml) was added until the pH was completely basic. The water layer was further washed with diethyl ether (3×100 mL). The mixed organic extracts were further extracted with 1 M NaOH (2×30 mL). This aqueous basic extract was mixed with the previous basic extract mixture, a small amount of $Na_2S_2O_4$ were added and the mixture was cooled down in an ice bath. Subsequently, conc. HCl, concentrated were added until the pH was acidic. The organic layer was extracted with EtOAc (4×150 mL). The combined organic extracts were washed with brine (2×20 mL), dried over sodium sulfate and concentrated *in vacuo*. The resulting residue were purified by silica flash chromatography [(90:10): DCM: [MeOH:AcOH (1-2%)]] to afford the substituted *N*-acetyl tryptophan (est. purity 85-90%), which was taken directly to the next step without further purification.

General Method B: Synthesis of tryptophan methyl esters

To a 0.2 M methanol solution of the substituted *N*-acetyltryptophan (0.28 mmol) at 0 °C, was added thionyl chloride (1.54 mmol, 6 equiv) dropwise. The mixture was warmed up to room temperature and then heated to 70 °C under reflux for 18 h. The mixture was cooled down and concentrated in vacuo. Et_2O (30 mL) was added and the mixture were stirred for 30 min. The suspension was filtered and washed numerous times with Et_2O until the solid was not sticky. The solid was air dried to afford the substituted tryptophan methyl ester hydrochloride salt.

General Method C1: Synthesis of *trans*-Pictet-Spengler methyl esters.[3, 4]
To a mixture of the requisite tryptophan methyl ester hydrochloride, (5 mmol), 4 Å molecular sieves (2.5 g, powder form) and 2,4-dichlorobenzaldehyde (5 mmol), DCM (18 mL) was added under a nitrogen atmosphere. The resulting mixture was stirred for 24-48 hours at room temperature until TLC analysis revealed the aldehyde was substantially consumed. TFA (10.0 mmol) was then added dropwise, and the reaction mixture was stirred at room temperature for an additional 4-14 days. An aqueous solution of $NaHCO_3$ (1.2 g, 14.3 mmol, in 10 mL H_2O) was added dropwise at 0 °C, followed by an addition of EtOAc (40 mL). After vigorous stirring for 15 min, the phases were separated and the aqueous layer was extracted with EtOAc (3×40 mL). The combined organic layers were washed with brine (15 mL), dried over $MgSO_4$, concentrated *in vacuo*, and purified by flash chromatography (5:5:1 hexane / DCM / EtOAc) to give *cis*- and *trans*- products. The *trans*-isomer is uniformly second-eluting, and the relative stereochemistry is unambiguously assigned by ^1H NMR.[5]

General Method C2: $Ti(O\text{-}iPr)_4$ assisted synthesis of *trans*-Pictet-Spengler isopropyl esters
Adapted from Horiguchi et al.[6] Tryptophan methyl ester (free-based from the commercial hydrochloride, 1.75 mmol) was mixed with 2,4-dichlorobenzaldehyde (2.45 mmol) and $Ti(O\text{-}iPr)_4$ (1 mL) under N_2, and the mixture was heated at 70 °C for overnight. To the reaction mixture was added trifluoroacetic anhydride (0.18 mmol) and TFA (1.75 mmol) at 0°C, then the mixture was heated at 70 °C for 1 day. The reaction was quenched by adding methanol and TiO_2 was removed by filtration. The filtrate was neutralized by 1 M NaOH solution and extracted with DCM. The organic layer was dried over $MgSO_4$ and concentrated *in vacuo*. The crude product was purified by column chromatography (Hexane: DCM: EtOAc =10:10:1).

General Method D: Catch and Release Hydrolysis of Pictet-Spengler Methyl Esters.[7]
To a solution of the *trans*-Pictet-Spengler methyl ester (0.2 mmol) in THF / MeOH / H_2O (2 mL / 2 mL / 2 mL) was added Amberlyst hydroxide resin (1.0 g, 4.2 mmol, Aldrich, loading: 4.2 mmol/g) at room temperature. *Note that the THF was freshly distilled from Na/benzophenone. We have found that THF peroxidation products, if present, give inseparable impurities in this protocol.* The reaction mixture was stirred for 24 hours, after which the resin was filtered and washed with MeOH and DCM alternatively (4 × 2 mL), to remove any starting material or any non-carboxylic acid impurities. An aqueous solution of AcOH (50%, 4 mL) was added to release the acid product from the resin, and the solution containing the product was collected by filtration. The resin beads were rinsed another 4 times with aq. AcOH (50%, 4 mL). The combined filtrates were concentrated *in vacuo*. The residue was suspended in a minimum amount of MeOH, and the desired product was precipitated by the addition of Et_2O and hexane. The suspension was stirred for 30 min and filtered. The solid was washed with hexane to afford the desired zwitterionic product.

General Method E: Synthesis of substituted *N*-Boc-3-iodoindoles.
Adapted from Liu et al.[8] To a solution of substituted indole (20 mmol) in DMF (40 mL) was added iodine (20.2 mmol) and KOH powder (50 mmol). The mixture was stirred at room temperature for 2h. The reaction mixture was then poured on an ice-cooled mixture of water (250 mL), ammonium hydroxide (13 mL), and sodium disulfite (10 g). The organic layer was

extracted with EtOAc (3×150 mL). The combined organic extracts were washed with 5% LiCl (3×25 mL), brine (30 mL), dried over sodium sulfate, and concentrated *in vacuo* to afford a residue which was immediately taken to the next step. The substituted 3-iodoindole was dissolved in DCM (50 mL) 4-Dimethylaminopyridine (DMAP) (10 mol %) and di-*tert*-butyl dicarbonate (25.5 mmol) were added and the mixture was stirred at room temperature overnight. At that point 1 M HCl was carefully added to the mixture until the pH was acidic, and the organic layer was extracted with DCM (3×100 mL), The combined organic extracts were washed with brine, dried over sodium sulfate and concentrated *in vacuo* to afford a residue. The residue was purified by silica flash chromatography to afford the desired substituted *N*-Boc-3-iodoindole.

General Method F: Ni-cat. reductive coupling route to enantiopure tryptophan methyl esters *Adapted from Liu et al.*[8] To a Schlenk tube were added $NiCl_2$ (10 mol%), 4,7-diphenyl-1,10-phenanthroline (BPhen) (10 mol%) and Mn powder (3 equiv). The Schlenk tube was attached to a vacuum line. The flask was evacuated and back-filled with nitrogen for at least 4 times. 1-methyl-2-pyrrolidinone (NMP) (10 mL) was added under nitrogen atmosphere and the was stirred at 80 °C for 45-60 min. Subsequently, the reaction was cooled down to room temperature. Simultaneously, a solution of the substituted *N*-Boc-3-iodoindole **25** (7 mmol, 1. equiv) in anhydrous and degassed NMP (5 mL), and a solution of Boc- β-iodo-Ala-OMe (8.4 mmol, 2 equiv) in anhydrous and degassed NMP (5 mL) were added to the Schlenk tube under a nitrogen flow. The reaction mixture was stirred at 25 °C for 24 h. Then, the reaction mixture was poured into ice water (250 mL) and the resulting mixture was extracted with EtOAc (4×100 mL). The combined organic extracts were washed with brine, dried over sodium sulfate and concentrated *in vacuo* to give a residue which was purified by silica flash chromatography to afford substituted Boc-protected tryptophan methyl esters. Removal of the Boc group was achieved by dropwise addition of 1 M HCl in EtOAc (31.9 mmol) to 3 mmol of the compound with stirring under nitrogen. After stirring for 24 h, EtOAc was evaporated *in vacuo*. Et_2O (50 mL) was added and the mixture was stirred for 30 min. The emulsion was filtered and washed numerous times with Et_2O until the solid was not sticky. The solid was air dried to afford the desired (*S*)-tryptophan methyl ester. hydrochloride salt.

General Method G: Negishi coupling route to enantiopure tryptophan methyl esters *Adapted from Ross et al.*[9] To an oven dried Schlenk tube was added zinc powder (17 mmol, 2.9 equiv) and 4 mL of anhydrous DMF under N_2. Iodine (0.58 mmol, 10 mol%) was added. When the solution turned back to colorless, Boc-β-iodo-Ala-OMe **25** (7.5 mmol, 1.3 equiv) was added followed by another portion of iodine (0.6 mol). The solution was stirred at room temperature for 5 mins. Substituted indole (5.8 mol, 1 equiv), $Pd_2(dba)_3$ (2 mol%) and SPhos (4 mol%) were added. The reaction mixture was stirred at 25 °C for 24 h. The reaction was quenched by adding water and EtOAc. The residual zinc powder was removed by filtration. The aqueous layer was extracted with EtOAc for 3 times. The combined organic layer was dried over by anhydrous $MgSO_4$ and concentrated *in vacuo*. The crude product was purified by column chromatography (Hexane: DCM: EtOAc =5:5:1) to afford the pure di-*N*-Boc-tryptophan methyl esters.

General Method H: The Boc groups of the di-*N*-Boc-tryptophan methyl esters were cleaved by dissolving the products in 1M HCl in EtOAc solution for 1 to 5 days. Over time a precipitate formed and was collected by filtration. The solid was air-dried to afford the desired tryptophan methyl ester hydrochloride salt. It was converted to the free base by the addition of DCM and sat. $NaHCO_3$. The organic layer was separated, dried over $MgSO_4$ and concentrated *in vacuo* to afford the free base tryptophan methyl esters.

Individual Procedures

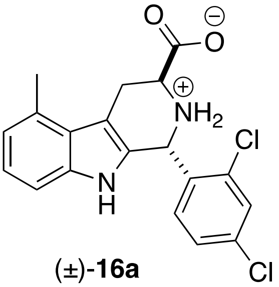

(±)-**16a**

(1*RS*,3*SR*)-1-(2,4-dichlorophenyl)-5-methyl-2,3,4,9-tetrahydro-1*H*-pyrido[3,4-*b*]indole-3-carboxylic acid (±)-16a (PRC1443)

<u>Isolation of (±)-4-methyltryptophan methyl ester</u>
Using General Method A1, 4-methylindole (1.1 g, 7.75 mmol, 95%), *dl*-Serine (1.63 g, 15.5 mmol), AcOH (9 mL), and Ac_2O (6 mL) were reacted and purified to afford (±)-*N*-acetyl-4-methyltryptophan (1.2 g, 62%, ~95% pure). Using General Method B, this material was converted to (±)-4-methyltryptophan methyl ester hydrochloride (1.0 g, 84%) as a beige brown solid.

<u>Isolation of *trans*-Pictet-Spengler ester</u>
Using General Method C, (±)-4-methyltryptophan methyl ester (988 mg, 3.5 mmol), 4 Å molecular sieves (1.7 g, powder form), 2,4-dichlorobenzaldehyde (612 mg, 3.5 mmol), DCM (26 mL) and DIPEA (0.59 mL, 3.43 mmol) were reacted. After 2 days, TFA (0.86 mL, 11.2 mmol) was then added dropwise, and the reaction mixture was stirred at room temperature for an additional 6 days and purified by column chromatography (5:5:0.5 hexane / DCM / EtOAc) affording the *cis*-ester (675 mg, 50% yield) as a white solid, and the desired *trans*-ester (330 mg, 24% yield) as an off-white solid.

<u>Ester hydrolysis</u>
Using General Method D, the *trans*-ester (97 mg, 0.25 mmol) in THF / MeOH / H_2O (2.5 mL / 2.5 mL / 2.5 mL) was treated with Amberlyst hydroxide resin (0.89 g, 3.75 mmol, Aldrich, loading: 4.2 mmol/g) at room temperature. Work up afforded a residue which was purified by preparative reverse phase HPLC. HPLC solvent A is 0.1% formic acid in filtered 17 MΩ H_2O and solvent B is 0.1% formic acid in HPLC grade acetonitrile. A non-linear gradient of 5-95%B over

30 minutes was used. Collected samples were frozen over dry ice and lyophilized to afford (±)-**16a** as a pale yellow solid (52 mg, 56%).
^{1}H NMR (400 MHz, CD_3OD) δ 7.71 (d, *J* = 2.1 Hz, 1H), 7.37 (ddd, *J* = 8.4, 2.2, 0.7 Hz, 1H), 7.12 – 7.03 (m, 2H), 7.00 (dd, *J* = 8.2, 7.0 Hz, 1H), 6.80 (dt, *J* = 7.0, 1.0 Hz, 1H), 6.38 (s, 1H), 3.95 (dd, *J* = 8.6, 5.4 Hz, 1H), 3.83 – 3.69 (m, 1H), 3.51 (ddd, *J* = 16.6, 8.7, 1.5 Hz, 1H), 2.69 (s, 3H).
^{13}C NMR (101 MHz, CD_3OD) δ 172.1, 137.4, 136.3, 135.8, 132.3, 131.8, 130.1, 129.6, 127.7, 125.4, 124.8, 122.4, 120.3, 108.8, 108.6, 53.6, 51.0, 24.9, 18.5.
HRMS (ESI) $[M+NH_4]^+$ calculated for $C_{19}H_{20}Cl_2N_3O_2$: 392.0927. Found: 392.0910

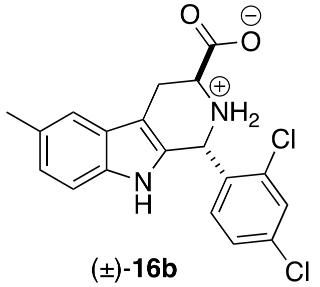

(±)-**16b**

(1*RS*,3*SR*)-1-(2,4-dichlorophenyl)-6-methyl-2,3,4,9-tetrahydro-1*H*-pyrido[3,4-*b*]indol-2-ium-3-carboxylate (±)-16b (PRC1449)

<u>Isolation of (±)-5-methyltryptophan methyl ester</u>

Using General Method A1, 5-methylindole (1.1 g, 7.75 mmol, 95%), *dl*-Serine (1.63 g, 15.5 mmol), AcOH (9 mL), and Ac_2O (6 mL) were reacted and purified afforded a residue which was purified by silica flash chromatography [(90:10): DCM: (MeOH:AcOH (1%), 0.95 g, 48%, 90% pure). Using General Method B, this material was dissolved in MeOH and treated with thionyl chloride (1.3 mL, 18.0 mmol), affording 5-methyltryptophan methyl ester hydrochloride (847 mg, 96%) as a dark purple solid.

<u>Isolation of *trans*-Pictet-Spengler ester</u>

Using General Method C, 5-methyltryptophan methyl ester hydrochloride (737 mg, 2.74 mmol), 4 Å molecular sieves (1.38 g, powder form) and 2,4-dichlorobenzaldehyde (480 mg, 2.74 mmol), DCE (20 mL) and DIPEA (0.47 mL, 2.68 mmol) were combined under nitrogen. After three days TFA (0.86 mL, 11.2 mmol) was then added dropwise, and the reaction mixture was stirred at room temperature for an additional 6 days. Aqueous work up followed by silica gel flash chromatography (5:5:0.5 hexane / DCM / EtOAc) afforded the *cis*-ester (253 mg, 24% yield) as a yellow solid, and the *trans*-ester (174 mg, 16% yield) as a light beige solid.

<u>Ester hydrolysis</u>

Using General Method D, the *trans*-ester (117 mg, 0.3 mmol) was dissolved in THF / MeOH / H_2O (3 mL / 3 mL / 3 mL) and Amberlyst hydroxide resin (1.07 g, 4.5 mmol, Aldrich, loading: 4.2 mmol/g) was added. Work up afforded a residue which was purified by preparative reverse phase HPLC. HPLC solvent A is 0.1% formic acid in filtered 17 MΩ H_2O and solvent B is 0.1% formic acid in HPLC grade acetonitrile. A non-linear gradient of 5-95%B over 30 minutes was used. Collected samples were frozen over dry ice and lyophilized to afford (±)-**16b** as a pale yellow solid (40 mg, 36%).

^{1}H NMR (400 MHz, CD_3OD) δ 7.70 (d, *J* = 2.1 Hz, 1H), 7.39 – 7.32 (m, 2H), 7.15 (dd, *J* = 8.2, 0.7 Hz, 1H), 7.02 (d, *J* = 8.5 Hz, 1H), 6.99 (ddd, *J* = 8.3, 1.7, 0.6 Hz, 1H), 6.36 (s, 1H), 3.94 (dd, *J* = 8.6, 5.4 Hz, 1H), 3.44 (dd, *J* = 16.4, 5.5 Hz, 1H), 3.20 (dd, *J* = 16.2, 8.6 Hz, 1H), 2.42 (s, 3H).
^{13}C NMR (101 MHz, CD_3OD) δ 172.3, 136.2, 135.8, 135.6, 132.2, 132.0, 129.6, 128.4, 127.6, 126.2, 126.0, 123.9, 117.5, 110.6, 107.8, 53.6, 51.1, 22.6, 20.1.
HRMS (ESI) $[M+H]^+$ calculated for $C_{19}H_{17}Cl_2N_2O_2$: 375.0662. Found: 375.0647.

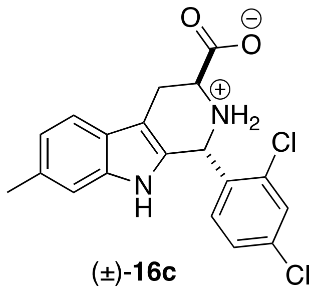

(±)-**16c**

(1*RS*,3*SR*)-1-(2,4-dichlorophenyl)-7-methyl-2,3,4,9-tetrahydro-1*H*-pyrido[3,4-*b*]indol-2-ium-3-carboxylate (±)-16c (PRC1467)

<u>Isolation of (±)-6-methyltryptophan methyl ester</u>

Using General Method A2, *dl*-Serine (2.2 g, 20.7 mmol), AcOH (34 mL), and Ac_2O (5.6 mL) wre combined and heated to 45 °C for 5 h. 6-methylindole (1.3 g, 10.0 mmol) was added and the reaction was heated to 80 °C for 1.5 h. Aqueous work up afforded a residue which was purified by silica flash chromatography [(90:10): DCM: [MeOH:AcOH (1%)]] to afford *N*-acetyl-6-methyltryptophan (1.6 g, 61%, 95% pure) as a beige solid. Using General Method B, this material (1.56 g, 6.0 mmol), was combined with thionyl chloride (2.41 mL, 33.0 mmol) in MeOH. Heating, workup, and precipitation afforded 6-methyltryptophan methyl ester hydrochloride (910 mg, 57%) as a beige-brown solid.

<u>Isolation of *trans*-Pictet-Spengler ester</u>

Using General Method C, 6-methyltryptophan methyl ester hydrochloride (894 mg, 3.0 mmol), 4 Å molecular sieves (1.5 g, powder form) and 2,4-dichlorobenzaldehyde (525 mg, 3.0 mmol), DCM (22 mL) and DIPEA (0.51 mL, 2.9 mmol) were were combined and stirred for 4 days at room temperature. TFA (0.74 mL, 9.6 mmol) was then added dropwise, and the reaction mixture was stirred at room temperature for an additional 2 weeks. Aqueous work up followed by silica gel flash chromatography (5:5:1 hexane / DCM / EtOAc) afforded *cis*-ester (304 mg, 26% yield) as a white solid, and *trans*-ester (448 mg, 38% yield) as a white solid.

<u>Ester hydrolysis</u>

Using General Method D, *trans*-ester (117 mg, 0.3 mmol) was dissolved in THF / MeOH / H_2O (3 mL / 3 mL / 3 mL) and Amberlyst hydroxide resin (1.1 g, 4.5 mmol, Aldrich, loading: 4.2 mmol/g) was added. Work up afforded a residue which was purified by preparative reverse phase HPLC. HPLC solvent A is 0.1% formic acid in filtered 17 MΩ H_2O and solvent B is 0.1% formic acid in HPLC grade acetonitrile. A non-linear gradient of 5-95%B over 30 minutes was used. Collected

samples were frozen over dry ice and lyophilized to afford (±)-**16c** as a bright yellow solid (48 mg, 43%).
^{1}H NMR (400 MHz, CD_3OD) δ 7.70 (d, *J* = 2.1 Hz, 1H), 7.42 (d, *J* = 8.1 Hz, 1H), 7.35 (dd, *J* = 8.4, 2.1 Hz, 1H), 7.17 – 7.00 (m, 2H), 6.92 (dd, *J* = 8.1, 1.4 Hz, 1H), 6.39 (s, 1H), 3.95 (br s, 1H), 3.45 (dd, *J* = 16.4, 5.1 Hz, 1H), 3.22 (dd, *J* = 16.2, 8.4 Hz, 1H), 2.40 (s, 3H).
^{13}C NMR (101 MHz, , CD_3OD) δ 172.1, 137.8, 136.3, 135.9, 132.3, 132.2, 131.8, 129.6, 127.7, 125.2, 123.7, 120.9, 117.6, 110.8, 108.1, 53.7, 51.1, 22.5, 20.4.
HRMS (ESI) $[M+H]^+$ calculated for $C_{19}H_{17}Cl_2N_2O_2$: 375.0662. Found: 375.0633.

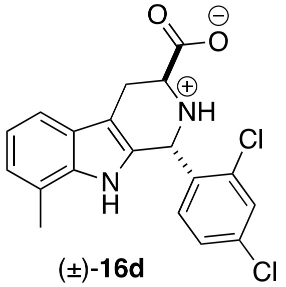

(±)-**16d**

(1*RS*,3*SR*)-1-(2,4-dichlorophenyl)-8-methyl-2,3,4,9-tetrahydro-1*H*-pyrido[3,4-*b*]indol-2-ium-3-carboxylate (±)-16d (PRC1464)

Isolation of (±)-7-methyltryptophan methyl ester

Using General Method A1, 7-methylindole (1.3 g, 10.0 mmol, 95%), *dl*-Serine (2.2 g, 20.75 mmol), AcOH (34 mL), and Ac_2O (5.6 mL) were combined. Heating and aqueous workup afforded *N*-acetyl-7-methyltryptophan (2.57 g, 99%, 90-95% pure) as a beige solid which was taken directly to the next step. Using General Method B, this material (2.3 g, 8.0 mmol, 90-95% pure), and thionyl chloride (3.21 mL, 44.0 mmol) were combined in MeOH. Heating, workup, and precipitation afforded 7-methyltryptophan hydrochloride (1.0 g, 50%) as an off-white beige solid.

Isolation of *trans*-Pictet-Spengler ester

Using General Method C, 7-methyltryptophan hydrochloride (1.0 g, 3.73 mmol), 4 Å molecular sieves (1.8 g, powder form) and 2,4-dichlorobenzaldehyde (653 mg, 3.73 mmol), DCM (28 mL) and DIPEA (0.64 mL, 3.66 mmol) were combined and stirred for 48 h at room temperature. TFA (0.91 mL, 11.9 mmol) was then added dropwise, and the reaction mixture was stirred at room temperature for an additional 1 week. Aqueous work up followed by silica gel flash chromatography (5:5:1 hexane / DCM / EtOAc) afforded *cis*-ester (702 mg, 48% yield) as an off-white solid and *trans*-ester (448 mg, 31% yield) as an off-white solid.

Ester hydrolysis

Using General Method D, *trans*-ester (101 mg, 0.26 mmol) was dissolved in THF / MeOH / H_2O (2.5 mL / 2.5 mL / 2.5 mL) and Amberlyst hydroxide resin (0.92 g, 3.9 mmol, Aldrich, loading: 4.2 mmol/g) was added. Work up afforded a residue which was purified by preparative reverse phase HPLC. HPLC solvent A is 0.1% formic acid in filtered 17 MΩ H_2O and solvent B is 0.1% formic acid in HPLC grade acetonitrile. A non-linear gradient of 5-95%B over 30 minutes was

used. Collected samples were frozen over dry ice and lyophilized to afford (±)-**16d** as a bright yellow solid (64 mg, 66%).
^{1}H NMR (400 MHz, CD_3OD) δ 7.71 (d, *J* = 2.1 Hz, 1H), 7.39 (d, *J* = 7.6 Hz, 1H), 7.36 (dd, *J* = 8.4, 2.1 Hz, 1H), 7.02 – 6.93 (m, 3H), 6.37 (s, 1H), 3.93 (dd, *J* = 9.2, 5.3 Hz, 1H), 3.54 – 3.44 (m, 1H), 3.20 (ddd, *J* = 16.4, 9.2, 1.5 Hz, 1H), 2.37 (s, 3H).
^{13}C NMR (126 MHz, CD_3OD) δ 172.1, 137.8, 136.3, 135.9, 132.3, 132.2, 131.8, 129.6, 127.7, 125.2, 123.7, 120.9, 117.6, 110.8, 108.1, 53.7, 51.1, 22.5, 20.4.
HRMS (ESI) $[M+H]^+$ calculated for $C_{19}H_{17}Cl_2N_2O_2$: 377.0612. Found: 377.0636.

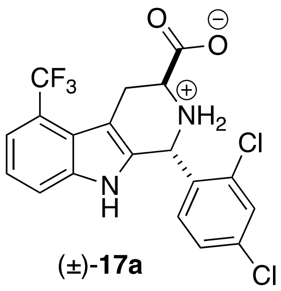

(±)-**17a**

(1*RS*,3*SR*)-1-(2,4-dichlorophenyl)-5-trifluoromethyl-2,3,4,9-tetrahydro-1*H*-pyrido[3,4-*b*]indol-2-ium-3-carboxylate (±)-17a (PRC1476)

Isolation of (±)-4-trifluoromethyltryptophan methyl ester

Using General Method A1, 4-trifluoromethylindole (1.1 g, 6.0 mmol), *dl*-Serine (1.73 g, 16.5 mmol), AcOH (20 mL), and Ac_2O (3.4 mL) were combined and heated to 75 °C for 12 h. Aqueous work up afforded a residue which was purified by silica flash chromatography [(90:10): DCM: [MeOH:AcOH (1%)]] to afford *N*-acetyl-4-trifluoromethyltryptophan (1.17 g, 62%, 80-85% pure) as an orange-red oil. Using the General Method B, this material (1.3 g, 3.7 mmol), and thionyl chloride (1.5 mL, 20.5 mmol) were combined in MeOH. Reflux, precipitation, and trituration afforded 4-trifluoromethyltryptophan methyl ester hydrochloride (800 mg, 74%) as an off-white solid.

Isolation of *trans*-Pictet-Spengler ester

Using General Method C, 4-trifluoromethyltryptophan methyl ester hydrochloride (650 mg, 2.0 mmol), 4 Å molecular sieves (1.0 g, powder form) and 2,4-dichlorobenzaldehyde (350 mg, 2.0 mmol), DCM (15 mL) and DIPEA (0.34 mL, 1.96 mmol) were stirred for 4 days at room temperature. TFA (0.5 mL, 6.4 mmol) was then added dropwise, and the reaction mixture was stirred at room temperature for an additional 3 weeks. Aqueous work up followed by silica gel flash chromatography (5:2:1:1 hexane / DCM /$CHCl_3$/ EtOAc) afforded *cis*-ester (123 mg, 14% yield) as a white solid and *trans*-ester (76 mg, 9% yield) as a white solid.

Ester hydrolysis

Using General Method D, *trans*-ester (76 mg, 0.17 mmol) was dissolved in THF / MeOH / H_2O (2.0 mL / 2.0 mL / 2.0 mL) and Amberlyst hydroxide resin (0.61 g, 2.6 mmol, Aldrich, loading: 4.2 mmol/g) was added. Work up afforded (±)-**17a** as a bright yellow solid (70 mg, 96%).

^{1}H NMR (400 MHz, CD_3OD) δ 7.71 (d, J = 2.1 Hz, 1H), 7.54 (d, J = 8.2 Hz, 1H), 7.45 (d, J = 7.4 Hz, 1H), 7.36 (dd, J = 8.4, 2.1 Hz, 1H), 7.26 (t, J = 7.8 Hz, 1H), 7.00 (d, J = 8.4 Hz, 1H), 6.38 (s, 1H), 3.96 (br s, 1H), 3.56 (br d, J = 14.2 Hz, 1H), 3.30 (br s, 1H).
^{13}C NMR (101 MHz, CD_3OD) δ 139.5, 137.6, 137.2, 133.8, 133.4, 131.1, 129.1, 126.3 (q, $^1J_{CF}$ = 270.7 Hz), 122.7, 122.5, 121.6 (q, $^2J_{CF}$ = 32.3 Hz), 118.5 (q, $^3J_{CF}$ = 6.0 Hz), 116.8, 108.5, 54.8, 52.3, 26.0; note: missing 2 resonances, one of which is the carbonyl carbon.
^{19}F NMR (376 MHz, CD_3OD) δ -59.3.
HRMS (ESI) $[M+H]^+$ calculated for $C_{19}H_{14}Cl_2F_3N_2O_2$: 429.0379. Found: 429.0373.

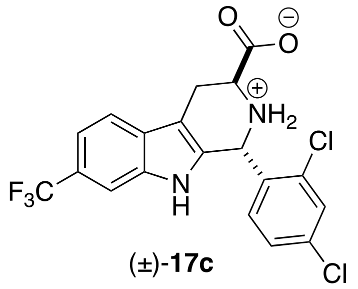

(±)-**17c**

(1*RS*,3*SR*)-1-(2,4-dichlorophenyl)-7-trifluoromethyl-2,3,4,9-tetrahydro-1*H*-pyrido[3,4-*b*]indol-2-ium-3-carboxylate (±)-17c (PRC1491)

<u>Isolation of (±)-6-trifluoromethyltryptophan methyl ester</u>

Using General Method A1, 6-(trifluoromethyl)-1*H*-indole (1.3 g, 10.0 mmol), *dl*-Serine (1.7 g, 16.5 mmol), AcOH (20.1 mL), and Ac_2O (3.4 mL) were heated to 80 °C for 2 h. Aqueous work up afforded a residue which was purified by silica flash chromatography [(90:10): DCM: [MeOH:AcOH (1%)]] to afford *N*-acetyl-6-trifluoromethyl tryptophan (1.34 g, 71%, 85-90% pure) as a bright yellow solid. Using General Method B, this material (1.3 g, 3.7 mmol), and thionyl chloride (1.5 mL, 20.5 mmol) were combined in MeOH. The mixture was refluxed at 70 °C for 18 h. Work up followed by precipitation afforded 6-trifluoromethyl tryptophan methyl ester hydrochloride (925 mg, 78%) as an off-white beige solid.
^{1}H NMR (400 MHz, CD_3OD) δ 7.72 (s, 1H), 7.69 (s, 1H), 7.45 – 7.40 (m, 1H), 7.32 (dd, J = 8.4, 1.7 Hz, 1H), 4.36 (dd, J = 7.1, 5.8 Hz, 1H), 3.79 (s, 3H), 3.53 – 3.37 (m, 2H). ^{19}F NMR (376 MHz, CD3OD) δ -62.0. 13C NMR (101 MHz, CD_3OD) δ 170.68, 137.06, 130.63, 128.89, 125.42 (q, $^1J_{CF}$ = 271.2 Hz), 124.73 (q, $^2J_{CF}$ = 31.5 Hz), 119.64, 116.68 (d, $^3J_{CF}$ = 3.6 Hz), 110.17 (d, $^3J_{CF}$ = 4.2 Hz), 108.19, 54.55, 53.70, 27.24. HRMS (ESI) [M+H]+ calculated for $C_{13}H_{14}F_3N_2O_2$: 287.1002. Found: 287.1004.

<u>Isolation of *trans*-Pictet Spengler ester</u>

Using General Method C, 6-trifluoromethyl tryptophan methyl ester hydrochloride (840 mg, 2.0 mmol), 4 Å molecular sieves (1.3 g, powder form) 2,4-dichlorobenzaldehyde (350 mg, 2.6 mmol), EtOH (9.5 mL) and DIPEA (0.44 mL, 2.55 mmol) were combined in a sealed tube were under nitrogen. The resulting mixture was stirred for 3 days at room temperature. TFA (0.2 mL, 2.6 mmol) was then added dropwise, and the reaction mixture was stirred at room temperature for an additional 3 days. Since TLC analysis indicated conversion was low, *p*-toluene sulfonic acid (447 mg) was added and the mixture was heated to 100 °C for 48 h when additional 2,4-dichlorobenzaldehyde (390 mg, 2.2 mmol) was added. Aqueous work up

followed by silica gel flash chromatography (2:1.5:0.5:0.5 hexane/DCM /$CHCl_3$/EtOAc) afforded cis-ethyl ester (135 mg, 12% yield) as a light beige solid, and *trans*-ethyl ester (225 mg, 20% yield) as a beige solid.

Ethyl ester hydrolysis

Using General Method D, *trans*-ester (116 mg, 0.23 mmol) was dissolved in THF/MeOH/H_2O (2.5 mL / 2.5 mL / 2.5 mL) and Amberlyst hydroxide resin (0.83 g, 3.5 mmol, Aldrich, loading: 4.2 mmol/g) was added at room temperature. Work up afforded **(±)-17c** as an off-white solid (64 mg, 65%, 91% pure).

^{1}H NMR (400 MHz, CD_3OD) δ 7.75 – 7.64 (m, 2H), 7.58 (s, 1H), 7.32 (ddd, *J* = 8.4, 6.5, 1.8 Hz, 2H), 6.98 (d, *J* = 8.4 Hz, 1H), 6.40 (s, 1H), 4.01 (d, *J* = 6.7 Hz, 1H), 3.48 (dd, *J* = 16.3, 5.4 Hz, 1H), 3.30 – 3.21 (m, 1H).

^{19}F NMR (376 MHz, CD_3OD) δ -61.9.

^{13}C NMR (101 MHz, CD_3OD) δ 175.1, 137.5, 137.2, 136.9, 133.1, 131.2, 130.9, 130.4, 129.3, 128.9, 127.7 (q, $^1J_{CF}$ = 271.2 Hz), 125.7 (q, $^2J_{CF}$ = 31.7 Hz), 119.8, 116.9 (q, $^3J_{CF}$ = 3.4 Hz), 109.8, 109.7 (q, $^3J_{CF}$ = 4.7 Hz), 54.4, 52.2, 23.7.

HRMS (ESI) [M+H]+ calculated for $C_{19}H_{14}Cl_2F_3N_2O_2$: 429.0379. Found: 429.0396.

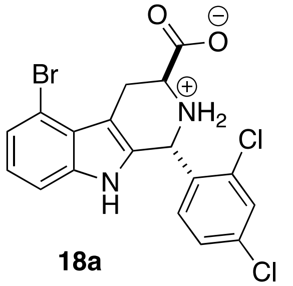

18a

(1*R*,3*S*)-1-(2,4-dichlorophenyl)-5-bromo-2,3,4,9-tetrahydro-1*H*-pyrido[3,4-*b*]indol-2-ium-3-carboxylate 18a (PRC1469, LL)

Synthesis of *N*-Boc-4-bromo-3-iodoindole **22a**

Using General Method E, 4-bromoindole (1.9 mL, 15.0 mmol), iodine (5.71 g, 22.5 mmol) and potassium hydroxide (2.10 g, 37.5 mmol) were combined in DMF (15 mL). After 4 h the reaction was poured onto ice and upon warming the substituted 3-iodoindole was isolated by filtration. This material was combined with 4-dimethylaminopyridine (183 mg, 1.5 mmol, 10 mol%) and di-*tert*-butyl dicarbonate (4.91 g, 22.5 mmol) in DCM (50 mL). After stirring for for 12 hours aqueous workup afforded a residue that was purified by flash chromatography (1 : 1 CH_2Cl_2/ hexanes) to give **22a** (4.19 g, 66% over two steps) as a white solid, mp: 125 °C (decomp).

^{1}H NMR (400 MHz, Chloroform-*d*) δ 8.26 (d, *J* = 8.3 Hz, 1H), 7.81 (br s, 1H), 7.43 (dd, *J* = 7.8, 0.9 Hz, 1H), 7.16 (dd, *J* = 8.3, 7.8 Hz, 1H), 1.66 (s, 9H).

^{13}C NMR (101 MHz, Chloroform-*d*) δ 148.2, 135.8, 133.4, 128.5, 126.7, 125.9, 115.4, 114.9, 85.1, 61.3, 28.2.

HRMS (ESI) $[M+H]^+$ calculated for $C_8H_6BrIN^+$ ($C_{13}H_{14}BrINO_2^+$ - CO_2, - $CH_2{=}C(CH_3)_2$): 321.8723, found 321.8720.

Synthesis of (*S*)-4-bromotryptophan methyl ester hydrochloride **27a**

Using General Method F, $NiCl_2$ (65 mg, 0.5 mmol, 10 mol%), BPhen (166 mg, 0.5 mmol, 10 mol%) and Mn powder (830 mg, 13.8 mmol) were added to a Schlenk tube equipped with a stir bar. The vessel was evacuated and filled with nitrogen (three cycles). To these solids, NMP (10 mL) was added under nitrogen atmosphere. The reaction mixture was stirred at 80 °C for 45 minutes. After cooling the reaction mixture to room temperature, **22a** (2.11 g, 5.0 mmol) and Boc-β-iodo-Ala-OMe (1.98 g, 6.0 mmol) in 1-methyl-2-pyrrolidinone (5 mL) was added under a positive flow of nitrogen. After 20 h, aqueous workup afforded a residue that was purified by column chromatography (5 : 5 : 1 hexanes/ CH_2Cl_2/ EtOAc) to give the di-Boc tryptophan methyl ester as a white solid. This material (1.67 g, 3.4 mmol) was then dissolved in 1 M HCl in EtOAc (30 mL) and stirred at room temperature for 36 hours. The precipitate was filtered and recrystallized with MeOH/ EtOAc to give **27a** (1.05 g, 69%) as a white solid, mp 126-127 °C (decomp). $[\alpha]_D^{23}$ = -16.3 (c = 0.59, MeOH).

^{1}H NMR (400 MHz, Methanol-*d*$_4$) δ 7.41 (dd, *J* = 8.0, 0.8 Hz, 1H), 7.27 (s, 1H), 7.24 (dd, *J* = 7.6, 0.8 Hz, 1H), 7.03 (t, *J* = 8.0, 7.6 Hz, 1H), 4.44 (dd, *J* = 9.8, 5.7 Hz, 1H), 3.88 (ddd, *J* = 14.8, 5.7, 0.7 Hz, 1H), 3.81 (s, 3H), 3.25 (ddd, *J* = 14.8, 9.8, 0.7 Hz, 1H).

^{13}C NMR (101 MHz, Methanol-*d*$_4$) δ 170.9, 140.0, 128.1, 126.1, 124.7, 124.0, 114.0, 112.4, 108.6, 56.0, 53.6, 28.8.

HRMS (ESI) $[M+H]^+$ calculated for $C_{12}H_{14}BrN_2O_2^+$: 297.0233, found 297.0232.

Isolation of *trans*-Pictet-Spengler ester

Using General Method C, **27a** (530 mg, 1.6 mmol), 4 Å molecular sieves (1.00 g), 2,4 dichlorobenzaldehyde (278 mg, 1.6 mmol) and DIPEA (276 µL, 1.6 mmol) were combined in CH_2Cl_2 (6 mL). After 24 hours, trifluoroacetic acid (244 µL, 3.2 mmol) was then added dropwise. After 48 h, aqueous workup afforded a residue that was purified by flash chromatography (3 : 1 hexanes/ EtOAc) to give the *trans*-ester (192 mg, 27%) as a white solid.

Ester hydrolysis

Using General Method D, the *trans*-ester (192 mg, 0.4 mmol) was dissolved in THF / MeOH / H_2O (2.0 mL / 1.0 mL / 1.0 mL) and Amberlyst hydroxide resin (3.00 g, Aldrich, loading: 4.2 mmol/g) was added. After 24 h, workup afforded a residue to which was added MeOH (0.5 mL), Et_2O (10 mL) and hexane (25 mL). The mixture was stirred for 15 minutes in an ice bath and then filtered. The solid was washed with hexane to afford **18a** (29 mg, 16%) as a pale yellow solid, m.p. decomp at 170 °C. $[\alpha]_D^{23}$ = -95.0 (c = 0.66, MeOH).

^{1}H NMR (400 MHz, Methanol-*d*$_4$) δ 7.71 (d, *J* = 2.1 Hz, 1H), 7.38 (dd, *J* = 8.4, 2.1 Hz, 1H), 7.25 (dd, *J* = 8.1, 0.8 Hz, 1H), 7.22 (dd, *J* = 7.7, 0.8 Hz, 1H), 7.07 – 6.94 (m, 2H), 6.34 (s, 1H), 4.00 – 3.87 (m, 1H), 3.63 – 3.52 (m, 1H).

^{13}C NMR (101 MHz, Methanol-*d*$_4$) δ 173.6, 139.7, 137.7, 137.2, 133.5, 133.5, 131.1, 129.6, 129.1, 126.1, 124.6, 124.4, 114.7, 111.8, 110.4, 54.6, 52.3, 26.2.

HRMS (ESI) $[M+H]^+$ calculated for $C_{18}H_{14}BrCl_2N_2O_2^+$: 438.9610, found 438.9581.

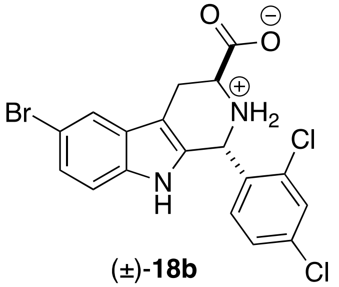

(±)-**18b**

(1*RS*,3*SR*)-1-(2,4-dichlorophenyl)-6-bromo-2,3,4,9-tetrahydro-1*H*-pyrido[3,4-*b*]indol-2-ium-3-carboxylate (±)-18b (PRC1431)

Isolation of (±)-5-bromotryptophan methyl ester

Using General Method A1, 5-bromoindole (1.0 g, 5.13 mmol), *dl*-Serine (1.1 g, 10.3 mmol), AcOH (12 mL), and Ac_2O (4.0 mL) were combined and heated. Aqueous work up afforded a residue which was purified by silica flash chromatography [(90:10): DCM: [MeOH:AcOH (1%)]] giving *N*-acetyl-5-bromotryptophan (1.04 g, 63%, 90% pure). Using General Method B, this material (894 mg, 2.47 mmol), and thionyl chloride (1.0 mL, 13.6 mmol) were combined in MeOH. Reflux, aqueous workup and precipitation afforded 5-bromotryptophan methyl ester hydrochloride (0.82 g, 99%, 85% pure) as a red-brown solid which was used without further purification.

Isolation of *trans*-Pictet-Spengler ester

Using General Method C, 5-bromotryptophan methyl ester hydrochloride (917 mg, 2.33 mmol,85% pure), 4 Å molecular sieves (1.15 g, powder form) and 2,4-dichlorobenzaldehyde (408 mg, 3.73 mmol), DCM (8 mL) were stirred for 48 h at room temperature. TFA (0.98 mL, 12.8 mmol) was then added dropwise, and the reaction mixture was stirred at room temperature for an additional 11 days. Aqueous work up followed by silica gel flash chromatography (5:5:1 hexane / DCM / EtOAc) afforded *cis*-ester (327 mg, 31% yield) as a light beige solid and *trans*-ester (182 mg, 17% yield) as a light beige solid.

Ester hydrolysis

Using General Method D, *trans*-ester (113 mg, 0.3 mmol) was dissolved in THF / MeOH / H_2O (2.5 mL / 2.5 mL / 2.5 mL) and Amberlyst hydroxide resin (0.89 g, 3.75 mmol, Aldrich, loading: 4.2 mmol/g) was added. Work up afforded a residue which was purified by preparative reverse phase HPLC. HPLC solvent A is 0.1% formic acid in filtered 17 MΩ H_2O and solvent B is 0.1% formic acid in HPLC grade acetonitrile. A non-linear gradient of 5-95%B over 30 minutes was used. Collected samples were frozen over dry ice and lyophilized to afford (±)-**18b** as an off white-bright yellow solid (34 mg, 31%).

^{1}H NMR (400 MHz, CD_3OD) δ 7.68 (dd, *J* = 6.8, 2.0 Hz, 2H), 7.33 (dd, *J* = 8.4, 2.1 Hz, 1H), 7.26 – 7.13 (m, 2H), 6.97 (d, *J* = 8.4 Hz, 1H), 6.31 (s, 1H), 3.91 (dd, *J* = 8.2, 5.4 Hz, 1H), 3.38 (dd, *J* = 16.2, 5.4 Hz, 1H), 3.17 (dd, *J* = 16.3, 8.2 Hz, 1H).

^{13}C NMR (101 MHz, CD_3OD) δ 172.6, 136.0, 135.8, 135.6, 132.6, 131.9, 129.6, 128.8, 127.7, 127.5, 124.9, 120.5, 112.5, 112.1, 108.3, 53.3, 51.1, 22.7.

HRMS (ESI) $[M+H]^+$ calculated for $C_{18}H_{14}BrCl_2N_2O_2$: 440.9587. Found: 440.9591.

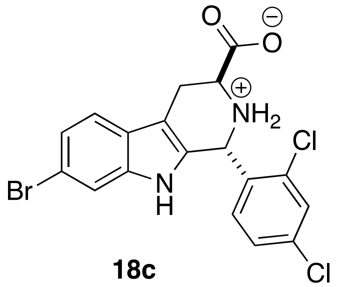

18c

(1*R*,3*S*)-1-(2,4-dichlorophenyl)-7-bromo-2,3,4,9-tetrahydro-1*H*-pyrido[3,4-*b*]indol-2-ium-3-carboxylate 18c (PRC1465, LL)

Synthesis of *N*-Boc-6-bromo-3-iodoindole **22c**

Using General Method E, 6-bromoindole (2.94 g, 15.0 mmol) was combined with iodine (5.71 g, 22.5 mmol) and potassium hydroxide (2.10 g, 37.5 mmol) in DMF (15 mL). The reaction mixture was stirred at room temperature for 4 hours. The reaction mixture was then poured into 400 mL of ice water containing 0.5% ammonia and 0.1% sodium thiosulfate. The mixture was placed in an ice bath for 2 hours to ensure the complete precipitation. The precipitate was filtered, washed with ice water and dried *in vacuo* to obtain crude product as a yellow solid. This material was combined with 4-dimethylaminopyridine (183 mg, 1.5 mmol, 10 mol%) and di-*tert*-butyl dicarbonate (4.91 g, 22.5 mmol) in CH_2Cl_2 (20 mL). After stirring for for 12 hours, aqueous workup afforded a residue was purified by flash chromatography (1 : 3 CH_2Cl_2/ hexanes) to give **22c** (4.76 g, 75% over two steps) as a white solid, mp 149-152 °C.

^{1}H NMR (400 MHz, Chloroform-*d*) δ 8.35 (br s, 1H), 7.68 (br s, 1H), 7.42 (dd, *J* = 8.4, 1.7 Hz, 1H), 7.25 (dd, *J* = 8.4, 0.6 Hz, 2H), 1.66 (s, 9H).

^{13}C NMR (101 MHz, Chloroform-*d*) δ 148.4, 135.5, 131.2, 130.7, 126.7, 122.8, 119.5, 118.4, 85.1, 65.0, 28.2 (^{1}H NMR and ^{13}C NMR match literature data)[10].

HRMS (ESI) $[M-H]^-$ calculated for $C_8H_4BrIN^-$ ($C_{13}H_{12}BrINO_2^-$ - CO_2, - $CH_2=C(CH_3)_2$): 319.8577, found 319.8556.

Synthesis of (*S*)-6-bromotryptophan methyl ester hydrochloride **27c**

Using General Method F, $NiCl_2$ (65 mg, 0.5 mmol, 10 mol%), Bphen (166 mg, 0.5 mmol, 10 mol%) and Mn powder (830 mg, 13.8 mmol) were added to a Schlenk tube equipped with a stir bar. The vessel was evacuated and filled with nitrogen (three cycles). To these solids, NMP (10 mL) was added under nitrogen atmosphere. The reaction mixture was stirred at 80 °C for 45 minutes. After cooling the reaction mixture to room temperature, **22c** (2.11 g, 5.0 mmol) and Boc-β-iodo-Ala-OMe (1.98 g, 6.0 mmol) in 1-methyl-2-pyrrolidinone (5 mL) was added under a positive flow of nitrogen. The reaction mixture was stirred at room temperature for 20 hours. Aqueous workup afforded a residue that was purified by column chromatography (5 : 5 : 1 hexanes/ CH_2Cl_2/ EtOAc) to give the di-Boc tryptophan methyl ester (1.32 g) as a white solid. This material (1.10 g, 2.2 mmol) was then dissolved in 1 M HCl in EtOAc (30 mL) and stirred at room temperature for 36 hours. The precipitate was filtered and recrystallized with MeOH/ EtOAc to give **27c** (707 mg, 51%) as a white solid. $[\alpha]_D^{23}$ = +13.9 (c = 1.20, MeOH)

^{1}H NMR (400 MHz, Methanol-d_4) δ 7.56 (dd, J = 1.8, 0.5 Hz, 1H), 7.45 (dd, J = 8.5, 0.5 Hz, 1H), 7.22 (s, 1H), 7.18 (dd, J = 8.5, 1.8 Hz, 1H), 4.33 (dd, J = 7.3, 5.6 Hz, 1H), 3.79 (s, 3H), 3.43 (ddd, J = 15.2, 5.6, 0.8 Hz, 1H), 3.36 (ddd, J = 15.2, 7.3, 0.8 Hz, 1H).
^{13}C NMR (101 MHz, Methanol-d_4) δ 170.7, 139.1, 127.2, 126.6, 123.5, 120.4, 116.4, 115.54, 108.0, 54.5, 53.7, 27.3.
HRMS (ESI) [M+H]$^+$ calculated for $C_{12}H_{14}BrN_2O_2^+$: 297.0233, found 297.0231.

<u>Isolation of *trans*-Pictet-Spengler ester</u>
Using General Method C, **27c** (500 mg, 1.5 mmol), 4 Å molecular sieves (1.00 g), 2,4 dichlorobenzaldehyde (263 mg, 1.5 mmol) and DIPEA (260 µL, 1.5 mmol) were combined in CH_2Cl_2 (6 mL). After 24 hours, trifluoroacetic acid (230 µL, 3.0 mmol) was then added dropwise. After 48 h, aqueous workup afforded a residue that was purified by flash chromatography (8 : 8 : 1 hexanes/ CH_2Cl_2/ EtOAc) to give the *trans*-ester (186 mg, 27%) as a white solid.

<u>Ester hydrolysis</u>
Using General Method D, *trans*-ester (154 mg, 0.3 mmol) was dissolved in THF / MeOH / H_2O (2.0 mL / 1.0 mL / 1.0 mL) and Amberlyst hydroxide resin (3.00 g, Aldrich, loading: 4.2 mmol/g) was added. After 24 h, workup afforded a residue to which was added MeOH (0.5 mL), followed by addition of Et_2O (10 mL) and hexane (25 mL). The mixture was stirred for 15 minutes in ice bath and then filtered. The solid was washed with hexane to afford **18c** (50 mg, 40%) as a light yellow solid, mp 170 °C (decomp). $[\alpha]_D^{23}$ = +0.4 (c = 1.59, MeOH).
^{1}H NMR (400 MHz, Methanol-d_4) δ 7.72 (d, J = 2.1 Hz, 1H), 7.49 (dd, J = 8.4, 0.5 Hz, 1H), 7.44 (dd, J = 1.7, 0.5 Hz, 1H), 7.38 (dd, J = 8.4, 2.1 Hz, 1H), 7.20 (dd, J = 8.4, 1.7 Hz, 1H), 7.03 (d, J = 8.4 Hz, 1H), 6.38 (s, 1H), 3.97 (dd, J = 8.1, 5.5 Hz, 1H), 3.45 (ddd, J = 16.2, 5.5, 1.3 Hz, 1H), 3.24 (ddd, J = 16.2, 8.1, 1.3 Hz, 1H).
^{13}C NMR (101 MHz, Methanol-d_4) δ 173.4, 139.4, 137.8, 137.3, 133.5, 133.3, 131.1, 129.1, 129.0, 126.2, 123.8, 120.8, 117.1, 115.2, 110.0, 54.9, 52.3, 23.8.
HRMS (ESI) [M+H]$^+$ calculated for $C_{18}H_{14}BrCl_2N_2O_2^+$: 438.9610, found 438.9621.

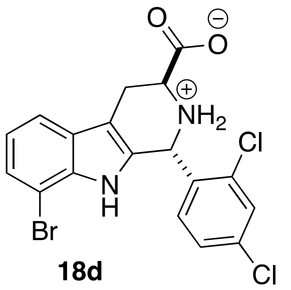

18d

(1*R*,3*S*)-1-(2,4-dichlorophenyl)-8-bromo-2,3,4,9-tetrahydro-1*H*-pyrido[3,4-*b*]indol-2-ium-3-carboxylate 18d (PRC1483, LL)
<u>Synthesis of *N*-Boc-7-bromo-3-iodoindole **22d**</u>
Using General Method E, 7-bromoindole (2.94 g, 15.0 mmol) was combined with iodine (5.71 g, 22.5 mmol) and potassium hydroxide (2.10 g, 37.5 mmol) in in DMF (15 mL). The reaction mixture was stirred at room temperature for 4 hours. The reaction mixture was then poured

into 400 mL of ice water containing 0.5% ammonia and 0.1% sodium thiosulfate. The mixture was placed in an ice bath for 2 hours to ensure the complete precipitation. The precipitate was filtered, washed with ice water and dried *in vacuo* to obtain crude product as a yellow solid. This material was combined with 4-dimethylaminopyridine (183 mg, 1.5 mmol, 10 mol%) and di-*tert*-butyl dicarbonate (4.91 g, 22.5 mmol) in CH_2Cl_2 (20 mL). After stirring for 12 hours, aqueous workup afforded a residue that was purified by flash chromatography (1 : 4 CH_2Cl_2/ hexanes) to give **22d** (1.61 g, 26% over two steps) as a white solid.
^{1}H NMR (400 MHz, Chloroform-*d*) δ 7.65 (br s, 1H), 7.60 (ddd, *J* = 7.7, 1.1, 0.4 Hz, 1H), 7.39 (dd, *J* = 7.7, 1.1 Hz, 1H), 7.19 (t, *J* = 7.7 Hz, 1H), 1.66 (s, 9H).
^{13}C NMR (101 MHz, Chloroform-*d*) δ 147.6, 135.5, 133.6, 133.5, 131.0, 124.71, 121.1, 107.6, 85.2, 64.4, 28.1.

<u>Synthesis of (*S*)-7-bromotryptophan methyl ester hydrochloride **27d**</u>
Using General Method F, $NiCl_2$ (48 mg, 0.4 mmol, 10 mol%), BPhen (123 mg, 0.4 mmol, 10 mol%) and Mn powder (610 mg, 11.1 mmol) were added to a Schlenk tube equipped with a stir bar. The vessel was evacuated and filled with nitrogen (three cycles). To these solids, NMP (10 mL) was added under nitrogen atmosphere. The reaction mixture was stirred at 80 °C for 45 minutes. After cooling the reaction mixture to room temperature, **22d** (1.56 g, 3.7 mmol) and Boc-β-iodo-Ala-OMe (1.46 g, 4.4 mmol) in 1-methyl-2-pyrrolidinone (5 mL) was added under a positive flow of nitrogen. After 20 h, aqueous workup afforded a residue that was purified by column chromatography (3 : 1 hexanes/ EtOAc) to give the di-Boc tryptophan methyl ester (713 mg) as a white solid. This material (1.217 g, 2.5 mmol) was then dissolved in 1 M HCl in EtOAc (30 mL) and stirred at room temperature for 36 hours. The precipitate was filtered and recrystallized with MeOH/ EtOAc to give **27d** (704 mg, 34%) as a white solid. $[\alpha]_D^{23}$ = +14.6 (c = 1.00, MeOH).
^{1}H NMR (400 MHz, Methanol-*d*$_4$) δ 7.54 (d, *J* = 7.8 Hz, 1H), 7.33 (d, *J* = 7.8 Hz, 1H), 7.29 (s, 1H), 7.00 (t, *J* = 7.8 Hz, 1H), 4.34 (dd, *J* = 7.2, 5.6 Hz, 1H), 3.79 (s, 3H), 3.48 – 3.32 (m, 2H).
^{13}C NMR (101 MHz, Methanol-*d*$_4$) δ 170.7, 136.7, 129.8, 126.8, 125.6, 121.6, 118.4, 109.0, 105.9, 54.5, 53.7, 27.5.
HRMS (ESI) $[M+H]^+$ calculated for $C_{12}H_{14}BrN_2O_2^+$: 297.0233, found 297.0231.

<u>Isolation of *trans*-Pictet-Spengler ester</u>
Using General Method C, **27d** (500 mg, 1.5 mmol) was combined with 4 Å molecular sieves (1.00 g), 2,4 dichlorobenzaldehyde (263 mg, 1.5 mmol) and DIPEA (260 µL, 1.5 mmol) in CH_2Cl_2 (6 mL). After 24 h, trifluoroacetic acid (230 µL, 3.0 mmol) was then added dropwise. After 48 h, aqueous workup afforded a residue that was purified by flash chromatography (8 : 8 : 1 hexanes/ CH_2Cl_2/ EtOAc) to give *trans*-ester (79 mg, 12%) as a white solid.

<u>Ester hydrolysis</u>
Using General Method D, *trans*-ester (79 mg, 0.2 mmol) was dissolved in THF / MeOH / H_2O (1.0 mL / 0.5 mL / 0.5 mL) and Amberlyst hydroxide resin (1.50 g, Aldrich, loading: 4.2 mmol/g) was added. After 24 h, workup afforded a residue to which was added MeOH (0.5 mL), followed by addition of Et_2O (10 mL) and hexane (25 mL). The mixture was stirred for 15 minutes in ice bath

and then filtered. The solid was washed with hexane to afford **18d** (30 mg, 39%) as an off-white solid, mp 160 °C (decomp). $[\alpha]_D^{23}$ = +61.6 (c = 0.88, MeOH).
^{1}H NMR (400 MHz, Methanol-d_4) δ 7.71 (d, *J* = 2.1 Hz, 1H), 7.57 (d, *J* = 7.7 Hz, 1H), 7.35 (dd, *J* = 8.4, 2.1 Hz, 1H), 7.34 (d, *J* = 7.7 Hz, 1H), 7.02 (t, *J* = 7.7 Hz, 1H), 6.96 (d, *J* = 8.4 Hz, 1H), 6.33 (s, 1H), 3.91 (s, 1H), 3.56 – 3.42 (m, 1H), 3.25 – 3.09 (m, 1H).
^{13}C NMR (101 MHz, Methanol-d_4) δ 168.7, 137.5, 137.3, 137.3, 133.4, 131.0, 129.3, 128.9, 128.8, 126.4, 121.9, 118.8, 113.5, 111.3, 105.6, 52.7, 32.8, 23.7.
HRMS (ESI) $[M+H]^+$ calculated for $C_{18}H_{14}BrCl_2N_2O_2^+$: 438.9610, found 438.9609.

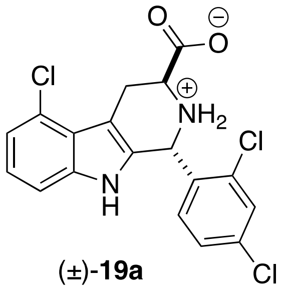

(±)-**19a**

(1*RS*,3*SR*)-1-(2,4-dichlorophenyl)-5-chloro-2,3,4,9-tetrahydro-1*H*-pyrido[3,4-*b*]indol-2-ium-3-carboxylate (±)-19a (PRC1466)

<u>Isolation of (±)-4-chlorotryptophan methyl ester</u>

Using General Method A1, 4-chloroindole (1.5 g, 10.0 mmol), *dl*-Serine (2.2 g, 20.7 mmol), AcOH (34 mL), and Ac_2O (5.6 mL) were combined and heated. Aqueous workup and afforded a residue which was purified by silica flash chromatography [(90:10): DCM: [MeOH:AcOH (1%)]] to giving *N*-acetyl4-chlorotryptophan (2.13 g, 76%) as an orange-red oil. Using General Method B, this material (2.0 g, 6.91 mmol, 95% pure) and thionyl chloride (2.78 mL, 38.0 mmol) were combined in MeOH. Heating, workup and precipitation afforded 4-chlorotryptophan methyl ester hydrochloride (1.6 g, 80%) as a beige solid.

<u>Isolation of *trans*-Pictet-Spengler ester</u>

Using General Method C, 4-chlorotryptophan methyl ester hydrochloride (1.15 g, 4.0 mmol), 4 Å molecular sieves (2.0 g, powder form) and 2,4-dichlorobenzaldehyde (700 mg, 3.73 mmol), DCM (30 mL) and DIPEA (0.68 mL, 3.9 mmol) were stirred for 48 h at room temperature. TFA (0.98 mL, 12.8 mmol) was then added dropwise, and the reaction mixture was stirred at room temperature for an additional 11 days. Aqueous work up followed by silica gel flash chromatography (5:5:1 hexane / DCM / EtOAc) afforded *cis*-ester (675 mg, 41% yield) as an off-white solid and *trans*-ester (491 mg, 30% yield) as a white solid.

<u>Ester hydrolysis</u>

Using General Method D, *trans*-ester (123 mg, 0.3 mmol) was dissolved in THF / MeOH / H_2O (3.0 mL / 3.0 mL / 3.0 mL) and Amberlyst hydroxide resin (1.1 g, 4.5 mmol, Aldrich, loading: 4.2 mmol/g) was added. Work up afforded a residue which was purified by preparative reverse phase HPLC. HPLC solvent A is 0.1% formic acid in filtered 17 MΩ H_2O and solvent B is 0.1% formic acid in HPLC grade acetonitrile. A non-linear gradient of 5-95%B over 30 minutes was

used. Collected samples were frozen over dry ice and lyophilized to afford (±)-**19a** as a bright yellow solid (44 mg, 37%).
^{1}H NMR (400 MHz, CD_3OD) δ 7.71 (d, *J* = 2.1 Hz, 1H), 7.37 (dd, *J* = 8.4, 2.1 Hz, 1H), 7.20 (dd, *J* = 7.8, 1.1 Hz, 1H), 7.12 – 6.99 (m, 3H), 6.37 (s, 1H), 3.96 (s, 1H), 3.85 (dd, *J* = 16.3, 4.9 Hz, 1H), 3.53 (dd, *J* = 16.6, 7.9 Hz, 1H).
^{13}C NMR (101 MHz, CD_3OD) δ 175.1, 137.5, 137.2, 136.9, 133.1, 131.2, 130.9, 130.4, 129.3, 128.9, 127.7 (q, $^1J_{CF}$ = 271.2 Hz), 125.7 (q, $^2J_{CF}$ = 31.7 Hz), 119.8, 116.9 (q, $^3J_{CF}$ = 3.4 Hz), 109.8, 109.7 (q, $^3J_{CF}$ = 4.7 Hz), 54.4, 52.2, 23.7.
HRMS (ESI) $[M+H]^+$ calculated for $C_{18}H_{14}Cl_3N_2O_2$: 396.0147. Found: 396.0124.

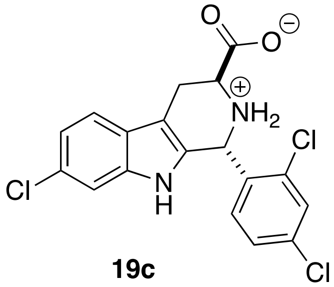

19c

(1*R*,3*S*)-1-(2,4-dichlorophenyl)-7-chloro-2,3,4,9-tetrahydro-1*H*-pyrido[3,4-*b*]indol-2-ium-3-carboxylate 19c (PRC1471)

Synthesis of (*S*)-6-chlorotryptophan methyl ester hydrochloride **28c**

Using the General Method B, commercial (*S*)-2-amino-3-(6-chloro-1*H*-indol-3-yl)propanoic acid **26** (954 mg, 3.8 mmol, 95%), and thionyl chloride (0.35 mL, 4.75 mmol) were combined in MeOH. Work up followed by precipitation afforded (*S*)-6-chlorotryptophan methyl ester hydrochloride **28c** (1.05g, 96%) as a beige-cream solid.
^{1}H NMR (400 MHz, CD_3OD) δ 7.50 (dd, *J* = 8.5, 0.6 Hz, 1H), 7.40 (dd, *J* = 1.9, 0.6 Hz, 1H), 7.23 (t, *J* = 0.7 Hz, 1H), 7.05 (dd, *J* = 8.5, 1.9 Hz, 1H), 4.33 (dd, *J* = 7.2, 5.6 Hz, 1H), 3.79 (s, 3H), 3.48 – 3.32 (m, 2H).
^{13}C NMR (101 MHz, CD_3OD) δ 169.3, 137.2, 127.4, 125.5, 125.2, 119.4, 118.6, 111.0, 106.5, 53.1, 52.3, 25.9.
HRMS (ESI) $[M+H]^+$ calculated for $C_{12}H_{14}ClN_2O_2$: 253.0738. Found: 253.0714.

Isolation of *trans*-Pictet-Spengler ester

Using General Method A1, **28c** (925 mg, 3.2 mmol), 4 Å molecular sieves (1.6 g, powder form) and 2,4-dichlorobenzaldehyde (560 mg, 3.2 mmol), were combined in DCM:MeOH (10:5 mL) After stirring for 48 h at room temperature. TFA (0.5 mL, 6.4 mmol) was then added dropwise, and the reaction mixture was stirred at room temperature for an additional 10 days. Aqueous work up followed by silica gel flash chromatography (5:5:0.5 hexane / DCM / EtOAc) afforded *cis*-ester (285 mg, 22% yield) as a yellow solid and *trans*-ester (166 mg, 13% yield) as an off-white solid.

Ester hydrolysis

Using General Method D, *trans*-ester (94 mg, 0.23 mmol) was dissolved in THF / MeOH / H_2O (2.5 mL / 2.5 mL / 2.5 mL) and Amberlyst hydroxide resin (0.82 g, 3.45 mmol, Aldrich, loading: 4.2 mmol/g) was added. Work up afforded a residue, to which was added MeOH (0.35 mL), and the product was precipitated by the addition of Et_2O (3.5 mL), and hexane (15 mL). The suspension was stirred for 30 min and filtered. The solid was washed with hexane to afford **19c** as a pale yellow solid (90 mg, 99%), $[\alpha]_D^{21}$= -8.0 (c = 0.36, MeOH).

^{1}H NMR (400 MHz, CD_3OD) δ 7.71 (d, *J* = 2.1 Hz, 1H), 7.52 (d, *J* = 8.4 Hz, 1H), 7.37 (dd, *J* = 8.4, 2.1 Hz, 1H), 7.27 (d, *J* = 1.8 Hz, 1H), 7.07 (dd, *J* = 8.4, 1.8 Hz, 1H), 7.03 (d, *J* = 8.4 Hz, 1H), 6.39 (s, 1H), 4.00 (dd, *J* = 8.0, 5.5 Hz, 1H), 3.45 (dd, *J* = 16.0, 4.5 Hz, 1H), 3.23 (dd, *J* = 16.2, 7.6 Hz, 1H).

^{13}C NMR (101 MHz, CD_3OD) δ 173.4, 139.0, 137.8, 137.3, 133.5, 133.2, 131.1, 129.6, 129.1, 128.9, 126.0, 121.2, 120.5, 112.2, 109.9, 54.8, 52.4, 23.8.

HRMS (ESI) $[M+H]^+$ calculated for $C_{19}H_{16}Cl_3N_2O_2$: 409.0272. Found: 409.0257.

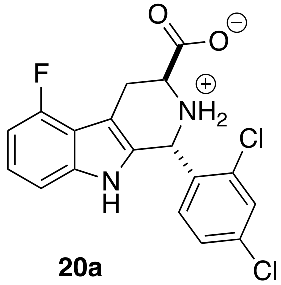

20a

(1*R*,3*S*)-1-(2,4-dichlorophenyl)-5-fluoro-2,3,4,9-tetrahydro-1*H*-pyrido[3,4-*b*]indol-2-ium-3-carboxylate 20a (PRC1474)

Synthesis of *N*-Boc-4-fluoro-3-iodoindole **23a**

Using General Method E, 4-fluoroindole (2.7 g, 20 mmol) was combined with iodine (5.14 g, 20.2 mmol) and KOH powder (2.8 g, 50 mmol) in DMF (40 mL). After 2 h, the reaction was subjected to workup and the residue was combined with DMAP (207 mg, 1.7 mmol) and di-*tert*-butyl dicarbonate (5.56 g, 25.5 mmol) in DCM (50 mL). After stirring overnight, aqueous workup and chromatography (20% DCM in hexane) afforded **23a** (6.0 g, 98%) as an off-white solid.

^{1}H NMR (400 MHz, $CDCl_3$) δ 7.99 (d, *J* = 8.4 Hz, 1H), 7.67 (s, 1H), 7.33 – 7.19 (m, 1H), 6.94 (ddd, *J* = 10.6, 8.1, 0.8 Hz, 1H), 1.66 (s, 9H).

^{13}C NMR (101 MHz, $CDCl_3$) δ 155.8 (d, $^1J_{CF}$ = 251.0 Hz), 148.3, 137.4 (d, $^3J_{CF}$ = 8.0 Hz), 131.1, 125.8 (d, $^3J_{CF}$ = 7.7 Hz), 119.8 (d, $^2J_{CF}$ = 16.9 Hz), 111.2 (d, $^4J_{CF}$ = 4.2 Hz), 109.0 (d, $^2J_{CF}$ = 18.7 Hz), 84.8, 55.6, 28.1.

^{19}F NMR (376 MHz, $CDCl_3$) δ -126.28 (dd, *J* = 10.6, 5.1 Hz).

Synthesis of (*S*)-4-fluorotryptophan methyl ester hydrochloride **29a**

Using General Method F, $NiCl_2$ (91 mg, 0.7 mmol), BPhen (232 mg, 0.7 mmol) and Mn powder (1.15 g, 21 mmol) were combined in NMP (10 mL) were stirred at 80 °C for 50 min and then cooled to room temperature. Simultaneously, a solution of **23a** (2.53 g, 7 mmol) in anhydrous, degassed NMP (5 mL), and a solution of Boc-β-iodo-Ala-OMe (2.76 g, 8.4 mmol) in anhydrous degassed NMP (5 mL) were added to the Schlenk tube under a nitrogen flow and stirred at

25 °C for 24 h. Aqueous work up afford a residue which was purified by silica flash chromatography (5% to 20% EtOAc in hexane) to afford the di-Boc protected amino acid ester (1.54 g) as a red oil. A portion of this material (1.4 g, 3.19 mmol), was subjected to 1 M HCl in EtOAc (32 mL, 32 mmol) at room temperature for 24 h. Work up followed by precipitation afforded **29a** (766 mg, 44% overall yield) as a beige solid.
^{1}H NMR (400 MHz, CD_3OD) δ 7.20 (dt, *J* = 8.2, 0.8 Hz, 1H), 7.16 (s, 1H), 7.12 – 7.03 (m, 1H), 6.73 (ddd, *J* = 11.6, 7.8, 0.8 Hz, 1H), 4.29 (dd, *J* = 8.7, 5.6 Hz, 1H), 3.81 (s, 3H), 3.56 (ddd, *J* = 14.9, 5.6, 0.9 Hz, 1H), 3.27 (ddd, *J* = 14.7, 8.8, 0.6 Hz, 1H).
^{13}C NMR (101 MHz, CD_3OD) δ 170.8, 158.1 (d, $^{1}J_{CF}$ = 242.0 Hz), 141.4 (d, $^{3}J_{CF}$ = 11.7 Hz), 126.2, 123.5 (d, $^{3}J_{CF}$ = 8.0 Hz), 116.6 (d, $^{2}J_{CF}$ = 20.1 Hz), 109.2 (d, $^{3}J_{CF}$ = 3.6 Hz), 106.3 (d, $^{4}J_{CF}$ = 2.5 Hz), 105.0 (d, $^{2}J_{CF}$ = 19.6 Hz), 55.2, 53.58, 29.0.
^{19}F NMR (376 MHz, CD_3OD) δ -127.30 (dd, *J* = 11.6, 5.2 Hz).
HRMS (ESI) $[M+H]^+$ calculated for $C_{12}H_{14}FN_2O_2$: 237.1034. Found: 237.1037.

Isolation of *trans*-Pictet-Spengler ester

Using General Method C, **29a** (763 mg, 2.8 mmol), 4 Å molecular sieves (1.35 g, powder form) and 2,4-dichlorobenzaldehyde (490 mg, 2.8 mmol), and DIPEA (0.48 mL, 2.75 mmol) were combined in DCM (21 mL). After stirring for 3 days at room temperature. TFA (0.7 mL, 9.0 mmol) was then added dropwise, and the reaction mixture was stirred at room temperature for an additional 4 days. Aqueous work up followed by silica gel flash chromatography (5:5:1 hexane / DCM / EtOAc) afforded *cis*-ester (413 mg, 38% yield) as a yellow solid, and *trans*-ester (296 mg, 27% yield) as an off-white solid.

Ester hydrolysis

Using General Method D, *trans*-ester (100 mg, 0.27 mmol) was dissolved in THF / MeOH / H_2O (2.5 mL / 2.5 mL / 2.5 mL) and Amberlyst hydroxide resin (0.95 g, 4.0 mmol, Aldrich, loading: 4.2 mmol/g) was added. Work up afforded a residue, to which was added MeOH (0.35 mL), and the product was precipitated by the addition of Et_2O (3.5 mL), and hexane (15 mL). The suspension was stirred for 30 min and filtered. The solid was washed with hexane to yield **20a** as a pale-yellow residue (90 mg, 94%), $[\alpha]_D^{21}$= -48 (c = 0.86, MeOH).
^{1}H NMR (400 MHz, CD_3OD) δ 7.68 (d, *J* = 2.0 Hz, 1H), 7.35 (dd, *J* = 8.3, 2.0 Hz, 1H), 7.10 – 6.96 (m, 3H), 6.76 – 6.65 (m, 1H), 6.34 (s, 1H), 4.02 – 3.87 (m, 1H), 3.68 – 3.51 (m, 1H), 3.46 – 3.32 (m, 1H).
^{13}C NMR (101 MHz, $CD_3OD/CDCl_3$) δ 173.6, 157.6 (d, $^{1}J_{CF}$ = 245.5 Hz), 140.4 (d, $^{3}J_{CF}$ = 11.3 Hz), 136.8, 136.1, 133.7, 132.6, 130.5, 128.3, 128.1, 123.6 (d, $^{3}J_{CF}$ = 7.3 Hz), 115.6 (d, $^{2}J_{CF}$ = 20.3 Hz), 108.2 (d, $^{4}J_{CF}$ = 3.6 Hz), 107.7, 104.9 (d, $^{2}J_{CF}$ = 18.7 Hz), 53.6, 51.9, 25.3.
^{19}F NMR (376 MHz, CD_3OD) δ -127.12 (d, *J* = 9.1 Hz).
HRMS (ESI) $[M+H]^+$ calculated for $C_{18}H_{14}Cl_2FN_2O_2$: 379.0411. Found: 379.0397.

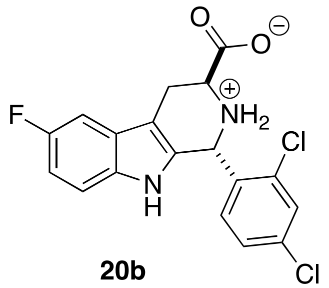

20b

(1*R*,3*S*)-1-(2,4-dichlorophenyl)-6-fluoro-2,3,4,9-tetrahydro-1*H*-pyrido[3,4-*b*]indol-2-ium-3-carboxylate 20b (PRC1659, HL)

Synthesis of *N*-Boc-5-fluoro-3-iodoindole **23b**

Using General Method E, 5-fluoroindole (1 g, 7.4 mmol) was combined with iodine (2.8 g, 11 mmol) and KOH powder (0.83 g, 15 mmol) in DMF (15 mL). After overnight reaction, the reaction was subjected to workup and the residue was combined with DMAP (45 mg, 5 mol%) and di-*tert*-butyl dicarbonate (1.9 g, 8.7 mmol) in DCM (15 mL). After stirring overnight, aqueous workup and chromatography (Hexane: EtOAc=20:1) afforded **23b** (1.47 g, 55%).

^{1}H NMR (400 MHz, Chloroform-*d*) δ 8.09 (s, 1H), 7.75 (s, 1H), 7.14 – 7.02 (m, 2H), 1.66 (d, *J* = 0.4 Hz, 9H).

^{13}C NMR (126 MHz, Chloroform-*d*) δ 159.9 (d, $^{1}J_{CF}$ = 240.6 Hz), 148.6, 133.6 (d $^{3}J_{CF}$ = 10.6 Hz), 131.8, 131.4, 116.4 (d, $^{3}J_{CF}$ = 9.0 Hz), 113.4 (d, $^{2}J_{CF}$ = 25.1 Hz), 107.4 (d, $^{2}J_{CF}$ = 25.0 Hz), 84.8, 64.5 (d, *J* = 4.1 Hz), 28.3.

^{19}F NMR (376 MHz, Chloroform-*d*) δ -119.05 – -120.00 (br).

Synthesis of (*S*)-5-fluorotryptophan methyl ester hydrochloride **29b**

Using General Method G, **23b** (1.47 g, 4.07 mmol), Boc-β-iodo-Ala-OMe (1.22 g, 3.7 mmol), Zinc power (0.7 g, 11 mmol), I_2 (46 mg+46 mg, 0.18 mmol+ 0.18 mmol), $Pd_2(dba)_3$ (84 mg, 2.5 mol%) and SPhos (76 mg, 5 mol%) were dissolved in 9 mL of dry DMF. After 16 hours, the reaction was subjected to workup. The crude product was purified by silica gel chromatography (Hexane:EtOAc=20:1), afforded the di-*N*-Boc-tryptophan methyl ester (0.84 g, 52%).

The Boc group was removed by using acetyl chloride in MeOH: To 15 mL of MeOH, 2.6 mL of acetyl chloride was added dropwise at 0 °C. It was stirred at room temperature for 1 hour, and then 1.05 g of Boc-protected tryptophan methyl ester was added. After 3 days, the solvent was removed *in vacuo*, affording the desired product **29b** (611 mg, 93%) without any purification.

$[\alpha]_D^{23}$=38.4 (c=5.7, MeOH).

^{1}H NMR (400 MHz, Methanol-*d*$_4$) δ 7.33 (dd, *J* = 8.8, 4.4 Hz, 1H), 7.23 (s, 1H), 7.19 (dd, *J* = 9.8, 2.3 Hz, 1H), 6.89 (td, *J* = 9.1, 2.3 Hz, 1H), 4.29 (t, *J* = 6.3 Hz, 1H), 3.78 (s, 3H), 3.28-3.38 (m, 2H).

^{13}C NMR (126 MHz, CD_3OD) δ 170.7, 159.1 (d, $^{1}J_{F-C}$ = 233.3 Hz), 134.7, 128.5 (d, $^{3}J_{F-C}$ = 9.8 Hz), 127.6, 113.5 (d, $^{3}J_{F-C}$ = 9.6 Hz), 111.1 (d, $^{2}J_{F-C}$ = 26.5 Hz), 107.7 (d, $^{4}J_{F-C}$ = 4.9 Hz), 103.5 (d, $^{2}J_{F-C}$ =24.0 Hz), 54.5, 53.8, 27.5.

^{19}F NMR (376 MHz, CD_3OD) δ -126.93 (td, *J* = 9.6, 4.6 Hz).

HRMS (ESI) calcd for $C_{12}H_{14}FN_2O_2$ [MH]+ 237.1034, found 237.1028.

Isolation of *trans*-Pictet-Spengler ester

Using General Method C, **29b** (611 mg, 2.2 mmol), 3,4-dichlorobenzaldehyde (432 mg, 2.46 mmol) and 4 Å molecular sieves (1.1 g, powder form) were dissolved in 7 mL of dry DCM under N_2. After overnight reaction, TFA (610 mg, 5.4 mmol) was added, and the reaction was stirred for another 2 days. Workup and silica gel chromatography (Hexane: DCM: EtOAc= 10:10:1) afforded the desired *trans*-product (120 mg, 12%). $[\alpha]_D^{23}$= -17.3 (c=0.11, MeOH)

^{1}H NMR (400 MHz, Chloroform-*d*) δ 7.62 (s, 1H), 7.48 (d, *J* = 2.1 Hz, 1H), 7.22 – 7.11 (m, 3H), 6.96 (d, *J* = 8.4 Hz, 1H), 6.92 (td, *J* = 9.1, 2.5 Hz, 1H), 5.86 (s, 1H), 3.87 (dd, *J* = 7.3, 5.1 Hz, 1H), 3.74 (s, 3H), 3.21 (ddd, *J* = 15.3, 5.1, 1.2 Hz, 1H), 3.07 (ddd, *J* = 15.3, 7.3, 1.5 Hz, 1H), 2.79 (s, 1H)

^{13}C NMR (151 MHz, $CDCl_3$) δ 173.7, 158.1 (d, $^{1}J_{C-F}$ = 235.4 Hz), 137.6, 134.6, 134.4, 133.6, 132.8, 131.0, 129.9, 127.4, 127.3 (d, $^{3}J_{C-F}$ = 9.8 Hz), 111.7 (d, $^{3}J_{C-F}$ = 9.5 Hz), 110.6 (d, $^{2}J_{C-F}$ = 26.3 Hz), 109.8 (d, $^{4}J_{C-F}$ = 4.4 Hz), 103.6 (d, $^{2}J_{C-F}$ = 23.5 Hz), 52.4, 52.3, 51.3, 24.8.

^{19}F NMR (565 MHz, $CDCl_3$) δ -124.20 (td, *J* = 9.4, 4.6 Hz).

HRMS (ESI) calcd for $C_{19}H_{16}Cl_2FN_2O_2$ [MH]+ 393.0567, found 393.0566

Ester hydrolysis

Using General Method D, *trans*-ester (120 mg, 0.31 mmol) was dissolved in THF / MeOH / H_2O (1.5 mL / 1.5 mL / 1.5 mL) and Amberlyst hydroxide resin (2 g) was added. Work up afforded a residue, to which was added MeOH (0.35 mL), and the product was precipitated by the addition of Et_2O (3.5 mL), and hexane (15 mL). The suspension was stirred for 3 days and filtered. The solid was washed with hexane to yield **20b** as a pale-yellow residue (95 mg, 82%). $[\alpha]_D^{23}$=-45.3 (c=0.95, MeOH).

^{1}H NMR (400 MHz, CD_3OD) δ 7.70 (d, *J* = 2.1 Hz, 1H), 7.36 (dd, *J* = 8.4, 2.1 Hz, 1H), 7.24 (d, *J* = 9.2 Hz, 1H), 7.23 (dd, *J* = 9.1, 1.9 Hz, 1H), 7.02 (d, *J* = 8.4 Hz, 1H), 6.92 (td, *J* = 9.3, 2.5 Hz, 1H), 6.38 (s, 1H), 3.97 (dd, *J* = 8.2, 5.5 Hz, 1H), 3.42 (ddd, *J* = 16.2, 5.5, 1.0 Hz, 1H), 3.21 (ddd, *J* = 16.1, 8.2, 1.5 Hz, 1H).

^{13}C NMR (101 MHz, CD_3OD) δ 173.6, 159.2 (d, $^{1}J_{C-F}$ = 233.8 Hz), 137.7, 137.2, 135.2, 133.6, 133.5, 131.1, 130.1, 129.1, 127.6 (d, $^{3}J_{C-F}$ = 10.1 Hz), 113.2 (d, $^{3}J_{C-F}$ = 9.6 Hz), 111.8 (d, $^{2}J_{C-F}$ = 26.6 Hz), 109.9 (d, $^{4}J_{C-F}$ = 4.7 Hz), 104.2 (d, $^{2}J_{C-F}$ = 23.9 Hz), 54.9, 52.5, 23.9.

^{19}F NMR (376 MHz, Methanol-d_4) δ -126.52 (td, *J* = 9.5, 4.3 Hz).

HRMS (ESI) calcd for $C_{18}H_{14}Cl_2FN_2O_2$ [MH]+ 379.0411, found379.0418.

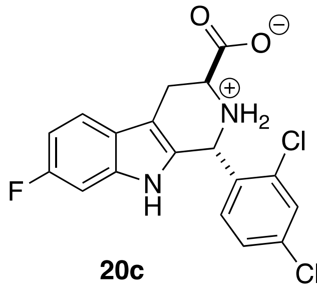

20c

(1*R*,3*S*)-1-(2,4-dichlorophenyl)-7-fluoro-2,3,4,9-tetrahydro-1*H*-pyrido[3,4-*b*]indol-2-ium-3-carboxylate 20c (PRC1479)

<u>Synthesis of *N*-Boc-6-fluoro-3-iodoindole **23c**</u>

Using General Method E, 6-fluoroindole (2.7 g, 20 mmol), iodine (5.14 g, 20.2 mmol) and KOH powder (2.8 g, 50 mmol) were combined in DMF (40 mL). The mixture was stirred at room temperature for 4 h. Aqueous work up afford 6-fluoro-3-iodoindole as an orange solid (5.0 g) This material was combined with 4-DMAP (232 mg, 1.9 mmol) and di-*tert*-butyl dicarbonate (6.22 g, 28.5 mmol) in DCM (50 mL) and stirred at room temperature overnight. Acidification followed by aqueous work up afford a residue that was purified by silica flash chromatography (20% DCM in hexane) to afford **23c** (5.9 g, 83% overall yield) as a white solid.

^{1}H NMR (400 MHz, $CDCl_3$) δ 7.87 (d, *J* = 10.2 Hz, 1H), 7.69 (s, 1H), 7.32 (ddd, *J* = 8.6, 5.3, 0.5 Hz, 1H), 7.06 (ddd, *J* = 9.1, 8.6, 2.4 Hz, 1H), 1.66 (s, 9H).

^{13}C NMR (101 MHz, $CDCl_3$) δ 161.6 (d, $^{1}J_{CF}$ = 241.8 Hz), 148.4, 134.8, 130.4 (d, $^{3}J_{CF}$ = 3.8 Hz), 128.4, 122.4 (d, $^{3}J_{CF}$ = 10.2 Hz), 111.7 (d, $^{2}J_{CF}$ = 24.6 Hz), 102.4 (d, $^{2}J_{CF}$ = 29.0 Hz), 84.8, 64.8 (d, $^{7}J_{CF}$ = 1.1 Hz), 28.1.

^{19}F NMR (376 MHz, $CDCl_3$) δ -116.21 (q, *J* = 9.5, 9.5, 4.7 Hz).

<u>Synthesis of (*S*)-6-fluorotryptophan methyl ester hydrochloride **29c**</u>

Using General Method F, $NiCl_2$ (91 mg, 0.7 mmol), BPhen (232 mg, 0.7 mmol) and Mn powder (1.15 g, 21 mmol) were combined in NMP (10 mL) were stirred at 80 °C for 40 min. After cooling to room temperature, solution of **23c** (2.53 g, 7 mmol) in anhydrous and degassed NMP (5 mL), and a solution of Boc- β-iodo-Ala-OMe (2.76 g, 8.4 mmol) in anhydrous and degassed NMP (5 mL) were added simultaneously to the Schlenk tube under a nitrogen flow and stirred at 25 °C for 24 h. Aqueous work up afford a residue which was purified by silica flash chromatography (5% to 20% EtOAc in hexane) to afford the di-Boc protected amino acid ester (1.55 g) as a red oil. A portion of this material (1.5 g, 3.5 mmol) was subjected to 1 M HCl in EtOAc (35 mL, 35 mmol) for 24 h. Work up followed by precipitation afforded **29c** (943 mg, 51% overall yield) as a beige solid.

^{1}H NMR (400 MHz, CD_3OD) δ 7.48 (dd, *J* = 8.7, 5.2 Hz, 1H), 7.19 (s, 1H), 7.08 (dd, *J* = 9.9, 2.2 Hz, 1H), 6.85 (ddd, *J* = 9.7, 8.7, 2.3 Hz, 1H), 4.32 (dd, *J* = 7.3, 5.5 Hz, 1H), 3.79 (s, 3H), 3.47 – 3.32 (m, 2H).

^{13}C NMR (101 MHz, CD_3OD) δ 169.3, 159.9 (d, $^{1}J_{CF}$ = 236.0 Hz), 136.8 (d, $^{3}J_{CF}$ = 12.6 Hz), 124.7 (d, $^{4}J_{CF}$ = 3.3 Hz), 123.5, 118.4 (d, $^{3}J_{CF}$ = 10.3 Hz), 107.4 (d, $^{2}J_{CF}$ = 25.0 Hz), 106.4, 97.1 (d, $^{2}J_{CF}$ = 26.0 Hz), 53.1, 52.2, 26.0.

^{19}F NMR (376 MHz, CD_3OD) δ -123.77 (td, *J* = 9.9, 5.3 Hz).

HRMS (ESI) $[M+H]^+$ calculated for $C_{12}H_{14}FN_2O_2$: 237.1034. Found: 237.1032.

Isolation of *trans*-Pictet-Spengler ester

Using General Method C, **29c** (570 mg, 3.25 mmol), 4 Å molecular sieves (1.6 g, powder form) and 2,4-dichlorobenzaldehyde (525 mg, 3.25 mmol), and DIPEA (0.55 mL, 3.2 mmol) were combined in DCE (12 mL). The resulting mixture was stirred for 48 h at room temperature and heated to 60 °C for 24 h. After cooling to room temperature, TFA (0.8 mL, 10.4 mmol) was then added dropwise, and the reaction mixture was stirred at room temperature for an additional 8 days. Aqueous work up followed by silica gel flash chromatography (5:5:1 hexane / DCM / EtOAc) afforded *cis*-ester (687 mg, 54% yield) as an off white solid and *trans*-ester (322 mg, 25% yield) as a beige solid.

Ester hydrolysis

Using General Method D, *trans*-ester (110 mg, 0.28 mmol) was dissolved in THF / MeOH / H_2O (3 mL / 3 mL / 3 mL) and Amberlyst hydroxide resin (1.0 g, 4.2 mmol, Aldrich, loading: 4.2 mmol/g) was added. Work up afforded a residue, to which was added MeOH (0.4 mL), and the product was precipitated by the addition of Et_2O (4.0 mL), and hexane (12 mL). The suspension was stirred for 30 min and filtered. The solid was washed with hexane to yield **20c** as an off-white solid (92 mg, 87%), $[\alpha]_D^{21}$= -32 (c = 0.83, MeOH).

^{1}H NMR (400 MHz, CD_3OD) δ 7.70 (d, *J* = 2.1 Hz, 1H), 7.52 (dd, *J* = 8.7, 5.3 Hz, 1H), 7.36 (dd, *J* = 8.4, 2.1 Hz, 1H), 7.02 (d, *J* = 8.4 Hz, 1H), 6.97 (dd, *J* = 9.8, 2.3 Hz, 1H), 6.86 (ddd, *J* = 9.7, 8.6, 2.3 Hz, 1H), 6.35 (s, 1H), 3.94 (dd, *J* = 8.2, 5.3 Hz, 1H), 3.44 (dd, *J* = 16.4, 5.3 Hz, 1H), 3.21 (dd, *J* = 16.2, 8.2 Hz, 1H).

^{13}C NMR (101 MHz, CD_3OD) δ 172.1, 160.3 (d, $^1J_{CF}$ = 236.9 Hz), 137.3 (d, $^3J_{CF}$ = 12.7 Hz), 136.3, 135.8, 132.1, 131.9, 129.6, 127.7, 126.93 (d, $^4J_{CF}$ = 3.1 Hz), 122.6, 118.9 (d, $^3J_{CF}$ = 10.3 Hz), 108.5, 107.6 (d, $^2J_{CF}$ = 24.8 Hz), 97.0 (d, $^2J_{CF}$ = 26.4 Hz), 53.5, 51.0, 22.5.

^{19}F NMR (376 MHz, CD_3OD) δ -122.30 (td, *J* = 9.7, 5.2 Hz).

HRMS (ESI) $[M+H]^+$ calculated for $C_{18}H_{14}Cl_2FN_2O_2$: 379.0411. Found 379.0397.

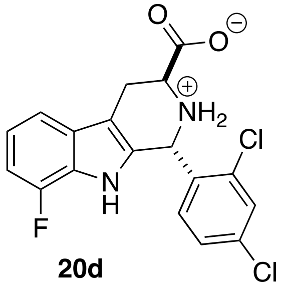

20d

(1*R*,3*S*)-1-(2,4-dichlorophenyl)-8-fluoro-2,3,4,9-tetrahydro-1*H*-pyrido[3,4-*b*]indol-2-ium-3-carboxylate 20d (PRC1490)

Synthesis of *N*-Boc-7-fluoro-3-iodoindole **23d**

Using General Method E, 7-fluoroindole (2.47 g, 18.25 mmol), iodine (4.68 g, 18.43 mmol) and KOH powder (2.56 g, 45.6 mmol) were combined in DMF (35 mL). After stirring for 4 h and aqueous workup, 7-fluoro-3-iodoindole was obtained as an orange oil (4.63 g) which was immediately taken to the next step. This material was combined with DMAP (216 mg, 1.8

mmol) and di-*tert*-butyl dicarbonate (5.78 g, 26.5 mmol) in DCM (45 mL). After stirring at room temperature overnight, the mixture was heated to reflux for 4.5 h. Acidification followed by aqueous work up afford a residue. The residue was purified by silica flash chromatography (20% DCM in hexane) to afford **23d** (6.1 g, 92% overall yield) as a clear liquid that became pink upon sitting.
^{1}H NMR (400 MHz, $CDCl_3$) δ 7.76 (d, *J* = 0.4 Hz, 1H), 7.29 – 7.22 (m, 1H), 7.20 (dd, *J* = 7.8, 1.3 Hz, 1H), 7.10 (dddd, *J* = 12.5, 7.6, 1.4, 0.4 Hz, 1H), 1.65 (s, 9H).
^{13}C NMR (101 MHz, $CDCl_3$) δ 149.5 (d, $^1J_{CF}$ = 254.0 Hz), 147.8, 136.0 (d, $^4J_{CF}$ = 3.4 Hz), 132.4, 124.0 (d, $^3J_{CF}$ = 7.2 Hz), 121.7 (d, $^2J_{CF}$ = 10.6 Hz), 117.6 (d, $^4J_{CF}$ = 3.8 Hz), 112.6 (d, $^2J_{CF}$ = 22.2 Hz), 84.8, 65.0 (d, $^6J_{CF}$ = 2.4 Hz), 27.9.
^{19}F NMR (376 MHz, $CDCl_3$) δ -115.86 (dd, *J* = 12.5, 3.8 Hz).

Synthesis of (*S*)-7-fluorotryptophan methyl ester hydrochloride **29d**
Using General Method F, $NiCl_2$ (91 mg, 0.7 mmol), BPhen (232 mg, 0.7 mmol) and Mn powder (1.15 g, 21 mmol) were combined in NMP (10 mL) were stirred at 80 °C for 45 min. After cooling to room temperature, a solution of **23d** (2.53 g, 7 mmol) in anhydrous and degassed NMP (5 mL), and a solution of Boc- β-iodo-Ala-OMe (2.76 g, 8.4 mmol) in anhydrous and degassed NMP (5 mL) were added to the Schlenk tube under a nitrogen flow and stirred at 25 °C for 24 h. Aqueous work up afford a residue which was purified by silica flash chromatography (5% to 20% EtOAc in hexane) to afford the di-Boc amino acid ester (2.03 g) as a red oil. A portion of this material (1.6 g, 3.7 mmol), was subjected to 1 M HCl in EtOAc (36 mL, 36 mmol) for 24 h. Work up followed by precipitation afforded **29d** (1.0 g, 66% overall yield) as an off-white solid.
^{1}H NMR (400 MHz, CD_3OD) δ 7.34 (dt, *J* = 8.0, 0.7 Hz, 1H), 7.24 (s, 1H), 7.02 (td, *J* = 7.9, 4.7 Hz, 1H), 6.88 (ddd, *J* = 11.4, 7.8, 0.8 Hz, 1H), 4.33 (dd, *J* = 7.4, 5.6 Hz, 1H), 3.79 (s, 3H), 3.45 (ddd, *J* = 15.2, 5.6, 0.8 Hz, 1H), 3.36 (ddd, *J* = 14.8, 7.4, 0.7 Hz, 1H).
^{13}C NMR (101 MHz, CD_3OD) δ 169.3, 149.8 (d, $^1J_{CF}$ = 243.4 Hz), 130.7 (d, $^3J_{CF}$ = 5.8 Hz), 125.2, 124.9 (d, $^2J_{CF}$ = 13.6 Hz), 119.2 (d, $^3J_{CF}$ = 6.3 Hz), 113.5 (d, $^4J_{CF}$ = 3.5 Hz), 107.2 (d, $^4J_{CF}$ = 2.3 Hz), 106.1 (d, $^2J_{CF}$ = 16.4 Hz), 53.1, 52.2, 26.1.
^{19}F NMR (376 MHz, CD_3OD) δ -136.87 (dd, *J* = 11.1, 4.5 Hz).
HRMS (ESI) $[M+H]^+$ calculated for $C_{12}H_{14}FN_2O_2$: 237.1034. Found: 237.1030.

Isolation of *trans*-Pictet-Spengler ester
Using General Method C, **29d** (886 mg, 3.25 mmol), 4 Å molecular sieves (1.6 g, powder form) 2,4-dichlorobenzaldehyde (570 mg, 3.25 mmol), and DIPEA (0.55 mL, 3.2 mmol) were combined in DCM (12 mL). After stirring for 48 h at room temperature. TFA (0.8 mL, 10.4 mmol) was added dropwise, and the reaction mixture was stirred at room temperature for an additional 10 days. Aqueous work up followed by silica gel flash chromatography (3:2:0.5 hexane / DCM / EtOAc) afforded *cis*-ester (706 mg, 63% yield) as a white solid and *trans*-ester (372 mg, 30% yield) as a white solid.

Ester hydrolysis
Using General Method D, *trans*-ester (110 mg, 0.28 mmol) was dissolved in THF / MeOH / H_2O (3 mL / 3 mL / 3 mL) and Amberlyst hydroxide resin (1.0 g, 4.2 mmol, Aldrich, loading: 4.2 mmol/g) was added. Work up afforded a residue, to which was added MeOH (0.35 mL), and the

product was precipitated by the addition of Et_2O (4.0 mL), and hexane (12 mL). The suspension was stirred for 30 min and filtered. The solid was washed with hexane to afford **20d** as a tan solid (78 mg, 74%), $[\alpha]_D^{21}$= -32 (c = 0.85, MeOH).
^{1}H NMR (400 MHz, CD_3OD) δ 7.70 (d, *J* = 2.1 Hz, 1H), 7.42 – 7.31 (m, 2H), 7.08 – 6.97 (m, 2H), 6.90 (ddd, *J* = 11.3, 7.8, 0.8 Hz, 1H), 6.36 (s, 1H), 3.94 (dd, *J* = 8.7, 5.4 Hz, 1H), 3.47 (ddd, *J* = 16.3, 5.4, 0.9 Hz, 1H), 3.21 (ddd, *J* = 16.3, 8.7, 1.5 Hz, 1H).
^{13}C NMR (126 MHz, CD_3OD) δ 173.6, 150.9 (d, $^1J_{CF}$ = 243.5 Hz), 137.7, 137.2, 133.4, 131.1, 131.1, 129.2, 129.0, 126.6 (d, $^2J_{CF}$ = 13.5 Hz), 121.0 (d, $^3J_{CF}$ = 6.1 Hz), 115.4 (d, $^4J_{CF}$ = 3.2 Hz), 110.7, 108.5 (d, $^2J_{CF}$ = 16.4 Hz), 54.6, 52.5, 24.2; note: missing one resonance.
^{19}F NMR (376 MHz, CD_3OD) δ -136.72 (dd, *J* = 11.5, 4.6 Hz).
HRMS (ESI) $[M+H]^+$ calculated for $C_{18}H_{14}Cl_2FN_2O_2$: 379.0411. Found: 379.0399.

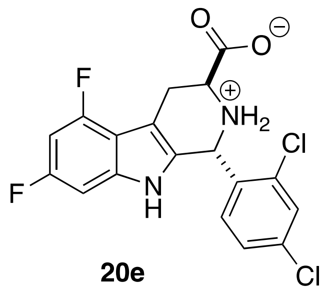

20e

(1*R*,3*S*)-1-(2,4-dichlorophenyl)-5,7-difluoro-2,3,4,9-tetrahydro-1*H*-pyrido[3,4-*b*]indol-2-ium-3-carboxylate 20e (PRC1470, LL)

Synthesis of *N*-Boc-4,6-difluoro-3-iodoindole **23e**

Using General Method E, 4,6-difluoroindole (1.00 g, 6.5 mmol) was combined with iodine (1.74 g, 6.88 mmol) and potassium hydroxide (0.92 g, 16.3 mmol) in DMF (10 mL). The reaction mixture was stirred at room temperature for 4 hours. The reaction mixture was then poured into 400 mL of ice water containing 0.5% ammonia and 0.1% sodium thiosulfate. The mixture was placed in an ice bath for 2 hours to ensure the complete precipitation. The precipitate was filtered, washed with ice water and dried *in vacuo* to obtain crude product as a yellow solid. It was used in the next step without further purification. This material was combined with 4-dimethylaminopyridine (80 mg, 0.7 mmol, 10 mol%) and di-*tert*-butyl dicarbonate (1.32 g, 6.5 mmol) in CH_2Cl_2 (20 mL). After 12 h, aqueous workup afforded a residue that was purified by flash chromatography (1 : 4 CH_2Cl_2/ hexanes) to give **23e** (1.91 g, 77% over two steps) as a white solid, mp 140-146 °C.
^{1}H NMR (400 MHz, Chloroform-*d*) δ 7.73 (br d, $^3J_{HF}$ = 9.4 Hz, 1H), 7.61 (s, 1H), 6.73 (ddd, $^3J_{HF}$ = 10.6, $^3J_{HF}$ = 9.4, $^4J_{HH}$ = 2.2 Hz, 1H), 1.66 (s, 9H).
^{13}C NMR (101 MHz, Chloroform-*d*) δ 160.8 (dd, $^1J_{CF}$= 242.7, $^3J_{CF}$ = 11.0 Hz), 155.4 (dd, $^1J_{CF}$ = 255.1, $^3J_{CF}$ = 14.2 Hz), 148.1, 137.0 (dd, $^3J_{CF}$ = 13.9, 8.5 Hz), 131.3 (d, $^3J_{CF}$ = 4.0 Hz), 116.5 (dd, $^2J_{CF}$ = 16.7, $^4J_{CF}$ = 2.9 Hz), 99.2 (dd, $^2J_{CF}$ = 28.4, 23.0 Hz), 99.0 (dd, $^2J_{CF}$ = 28.9, $^4J_{CF}$ = 5.1 Hz), 85.4, 55.4, 28.2.
^{19}F NMR (376 MHz, Chloroform-*d*) δ -112.80 (m), -122.45 (dd, $^3J_{HF}$ = 10.6 Hz, $^4J_{FF}$ = 4.5 Hz).

Synthesis of (*S*)-4,6-difluorotryptophan methyl ester hydrochloride **29e**

Using General Method F, $NiCl_2$ (46 mg, 0.4 mmol, 10 mol%), BPhen (116 mg, 0.4 mmol, 10 mol%) and Mn powder (577 mg, 10.5 mmol) were added to a Schlenk tube equipped with a stir bar. The vessel was evacuated and filled with nitrogen (three cycles). To these solids, NMP (10 mL) was added under nitrogen atmosphere. The reaction mixture was stirred at 80 °C for 45 minutes. After cooling the reaction mixture to room temperature, **23e** (1.33 g, 3.5 mmol) and Boc-β-iodo-Ala-OMe (1.38 g, 4.2 mmol) in 1-methyl-2-pyrrolidinone (5 mL) were added under a positive flow of nitrogen. After 20 h, aqueous workup afforded a residue that was purified by column chromatography (10 : 10 : 1 hexanes/ CH_2Cl_2/ EtOAc) to give the di-Boc tryptophan methyl ester (1.06 g) as a white solid. This material (1.05 g, 2.3 mmol) was then dissolved in 1 M HCl in EtOAc (30 mL) and stirred at room temperature for 36 hours. The precipitate was filtered and recrystallized with MeOH/ EtOAc to give **29e** (539 mg, 58%) as a white solid, mp 200 °C (decomp). $[\alpha]_D^{23}$ = +5.5 (c = 1.39, MeOH).
^{1}H NMR (400 MHz, Methanol-d_4) δ 7.17 (s, 1H), 6.96 (dd, $^3J_{HF}$ = 9.6, $^4J_{HH}$ = 2.1 Hz, 1H), 6.64 (ddd, $^3J_{HF}$ = 11.8, 9.6, $^4J_{HH}$ = 2.1 Hz, 1H), 4.27 (dd, J = 8.7, 5.7 Hz, 1H), 3.81 (s, 3H), 3.49 (ddd, J = 14.7, 5.7, 0.9 Hz, 1H), 3.29 – 3.24 (m, 1H).
^{13}C NMR (101 MHz, Methanol-d_4) δ 170.8, 159.5 (dd, $^1J_{CF}$ = 237.7, $^3J_{CF}$ = 11.6 Hz), 156.1 (dd, $^1J_{CF}$ = 243.5, $^3J_{CF}$ = 15.5 Hz), 140.2 (t, $^3J_{CF}$ = 13.9 Hz), 126.6 (d, $^4J_{CF}$ = 2.3 Hz), 113.3 (dd, $^2J_{CF}$ = 19.6, $^4J_{CF}$ = 1.4 Hz), 106.7 (d, $^3J_{CF}$ = 2.7 Hz), 95.6 (dd, $^2J_{CF}$ = 28.8, 23.9 Hz), 95.2 (dd, $^2J_{CF}$ = 25.9, $^4J_{CF}$ = 4.6 Hz), 55.0, 53.6, 28.8.
^{19}F NMR (376 MHz, Methanol-d_4) δ -120.99 (td, $^3J_{HF}$ = 9.6, $^4J_{FF}$ = 4.1 Hz), -123.80 (dd, $^3J_{HF}$ = 11.5, $^4J_{FF}$ = 4.1 Hz).

HRMS (ESI) $[M+H]^+$ calculated for $C_{12}H_{13}F_2N_2O_2^+$: 255.0940, found 255.0956.

<u>Isolation of *trans*-Pictet-Spengler ester</u>
Using General Method C, **29e** (290 mg, 1.0 mmol) was combined with 4 Å molecular sieves (0.50 g), 2,4 dichlorobenzaldehyde (175 mg, 1.0 mmol) and DIPEA (174 µL, 1.0 mmol) in CH_2Cl_2 (6 mL). After stirring for 24 hours, trifluoroacetic acid (153 µL, 2.0 mmol) was then added dropwise. After 48 h aqueous workup afforded a residue that was purified by flash chromatography (5 : 1 hexanes/ EtOAc) to give *trans*-ester (101 mg, 25%) as a white solid.

<u>Ester hydrolysis</u>
Using General Method D, *trans*-ester (100 mg, 0.2 mmol) was dissolved in THF / MeOH / H_2O (1.0 mL / 0.5 mL / 0.5 mL) and Amberlyst hydroxide resin (2.00 g, Aldrich, loading: 4.2 mmol/g) was added. After 24 h, workup afforded a residue to which was added MeOH (0.5 mL), followed by addition of Et_2O (10 mL) and hexane (25 mL). The mixture was stirred for 15 minutes in ice bath and then filtered. The solid was washed with hexane to afford **20e** (49 mg, 51%) as an off-white solid, m.p. 170 °C (decomp). $[\alpha]_D^{23}$ = -38.8 (c = 1.18, MeOH).
^{1}H NMR (400 MHz, Methanol-d_4) δ 7.71 (d, J = 2.1 Hz, 1H), 7.38 (dd, J = 8.4, 2.1 Hz, 1H), 7.05 (d, J = 8.4 Hz, 1H), 6.83 (dd, J = 9.3, 2.0 Hz, 1H), 6.64 (ddd, J = 10.8, 10.1, 2.0 Hz, 1H), 6.33 (s, 1H), 3.96 (s, 1H), 3.64 – 3.48 (m, 1H), 3.42 – 3.34 (m, 1H).
^{13}C NMR (101 MHz, Methanol-d_4) δ 173.4, 161.0 (dd, $^1J_{CF}$ = 238.4, $^3J_{CF}$ = 11.8 Hz), 157.5 (dd, $^1J_{CF}$ = 247.2, $^3J_{CF}$ = 15.0 Hz), 140.2 (t, $^3J_{CF}$ = 14.4 Hz), 137.7, 137.2, 133.4, 131.1, 129.1, 128.8 (d, $^4J_{CF}$

=2.8 Hz), 112.8 (d, $^2J_{CF}$ =20.3 Hz), 108.0, 95.8 (dd, $^2J_{CF}$ =29.2, 23.4 Hz), 95.0 (dd, $^2J_{CF}$ =26.4, $^4J_{CF}$ =4.4 Hz), 54.9, 52.2, 25.1 (two Cl-bearing carbons in D ring are accidentally equivalent).
^{19}F NMR (376 MHz, Methanol-d_4) δ -119.82 (td, $^3J_{HF}$ = 9.7, $^4J_{FF}$ = 3.8 Hz), -123.72 (dd, $^3J_{HF}$ = 10.7, $^4J_{FF}$ = 3.8).
HRMS (ESI) $[M+H]^+$ calculated for $C_{18}H_{13}Cl_2 F_2N_2O_2^+$: 397.0317, found 397.0283.

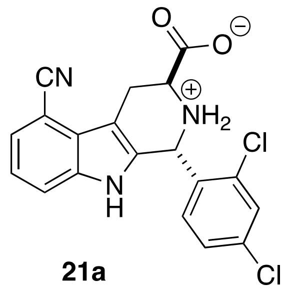

21a

(1*R*,3*S*)-1-(2,4-dichlorophenyl)-5-cyano-2,3,4,9-tetrahydro-1*H*-pyrido[3,4-*b*]indol-2-ium-3-carboxylate 21a (PRC1599, HL)

Synthesis of *N*-Boc-4-cyano-3-iodoindole **24a**

Using General Method E, 1*H*-indole-4-carbonitrile (2 g, 14 mmol), iodine (6.1 g, 24 mmol) and KOH powder (2.36 g, 42 mmol) were combined in DMF (18 mL). After stirring for 12 h and aqueous workup, *tert*-butyl 4-cyano-3-iodo-1H-indole-1-carboxylate was obtained, which was immediately taken to the next step. This material was combined with DMAP (90 mg, 0.75 mmol) and di-*tert*-butyl dicarbonate (4.8 g, 22 mmol) in DCM (35 mL). After stirring at room temperature overnight, imidazole (0.8 g, 11.8 mmol) was added and stirred for 15 min. Acidification followed by aqueous work up afford a residue 24a (5.07 g, 98% overall yield).
^{1}H NMR (400 MHz, $CDCl_3$) δ 8.49 (d, J = 8.5 Hz, 1H), 7.88 (s, 1H), 7.65 (dd, J = 7.5, 0.9 Hz, 1H), 7.50 – 7.39 (m, 1H), 1.68 (s, 9H).
^{13}C NMR (101 MHz, $CDCl_3$) δ 148.0, 135.3, 134.1, 130.2, 129.6, 125.0, 120.04, 116.6, 105.0, 85.7, 60.7, 28.2.
HRMS (ESI) calcd for $C_{14}H_{14}IN_2O_2$ [MH]+ 369.0094, found 369.0092.

Synthesis of (*S*)-4-cyanotryptophan methyl ester hydrochloride **30a**

Using General Method G, Zinc powder (2.46 g, 37 mmol) was added in 8 mL of dry DMF under N_2, I_2 (480, 313 µmol), methyl (*R*)-2-((*tert*-butoxycarbonyl)amino)-3-iodopropanoate (4.12 g, 12.5 mmol) and another portion of I_2 (480, 313 µmol) were sequentially added. When the solution turned back to colorless, $Pd_2(dba)_3$ (286 mg, 313 µmol), SPhos (257 mg, 626 µmol) and **24a** (4.12 g, 12.52 mmol) were added. It was stirred at room temperature for 20 hours. Workup and purification by silica chromatography (Hexane: EtOAc=9:1 to 1:1) afforded *tert*-butyl (*S*)-3-(2-((*tert*-butoxycarbonyl)amino)-3-methoxy-3-oxopropyl)-4-cyano-1*H*-indole-1-carboxylate (2.16 g, 39%). Following general method H, *tert*-butyl (S)-3-(2-((*tert*-butoxycarbonyl)amino)-3-methoxy-3-oxopropyl)-4-cyano-1*H*-indole-1-carboxylate (2.16, 4.9 mmol) was dissolved in 30mL of 1M HCl in EtOAc. It was stirred at RT for 48 hours. The precipitate was filtered out and washed with 10 mL DCM and 10 mL of sat. $NaHCO_3$ solution. The organic layer was separated, dried over $MgSO_4$, and concentrated *in vacuo* to give free base **30a** (0.65 g, 45%). $[\alpha]_D^{23}$=6.27 (c=2.55, MeOH)

^{1}H NMR (500 MHz, CD_3OD) δ 7.74 (d, *J* = 8.2 Hz, 1H), 7.51 (d, *J* = 7.4 Hz, 1H), 7.45 (s, 1H), 7.28 (t, *J* = 7.8 Hz, 1H), 4.41 (dd, *J* = 9.0, 6.4 Hz, 1H), 3.74 (dd, *J* = 15.3, 6.3 Hz, 1H), 3.42 (dd, *J* = 15.2, 9.0 Hz, 1H).
^{13}C NMR (126 MHz, CD_3OD) δ 169.2, 137.3, 128.3, 126.0, 125.8, 121.3, 119.3, 116.9, 106.5, 100.4, 53.6, 52.2, 26.1.
HRMS (ESI) calcd for $C_{13}H_{14}N_3O_2$ [MH]+ 244.1081, found 244.1071.

Isolation of *trans*-Pictet-Spengler ester

30a (200 mg, 0.71 mmol) and 2,4-dichlorobenzaldehyde (188 mg, 1.1 mmol), and $MgSO_4$ (172 mg, 1.43 mmol) were dissolved in 1.5 mL DCM. After 16 hours, the $MgSO_4$ was removed by filtration, and the filtrate was concentrated *in vacuo*. The residue was redissolved in TFA (407 mg, 3.57 mmol), TFAA (30 mg, 0.13 mmol) and Ti(O*i*Pr)$_4$ (203 mg, 715 mmol) and it was heated to 70 ºC for 1 day. The reaction was quenched by adding sat. $NaHCO_3$ solution to pH=8. The precipitate was filtered, and the filtrate was extracted with EtOAc 15 mL for 3 times. The organic portions were combined, dried over $MgSO_4$ and concentrated *in vacuo*. The crude product was purified by silica chromatography (Hexane: DCM: EtOAc=10:10:1) affording *trans*-ester (45 mg 16%). $[\alpha]_D^{23}$=-87.8 (c=2.05, MeOH).
^{1}H NMR (400 MHz, $CDCl_3$) δ 8.11 (s, 1H), 7.51 – 7.42 (m, 3H), 7.19 (dd, *J* = 8.1, 7.4 Hz, 1H), 7.16 (dd, *J* = 8.4, 2.2 Hz, 1H), 7.01 (dd, *J* = 8.6, 2.2 Hz, 1H), 5.95 (s, 1H), 3.93 (dd, *J* = 6.9, 5.3 Hz, 1H), 3.74 (d, *J* = 0.6 Hz, 3H), 3.58 (ddd, *J* = 16.1, 5.3, 1.2 Hz, 1H), 3.43 (ddd, *J* = 15.8, 6.8, 1.5 Hz, 1H).

^{13}C NMR (126 MHz, $CDCl_3$) δ 173.6, 137.1, 136.2, 135.0, 134.4, 131.0, 130.0, 127.6, 127.0, 126.0, 122.0, 119.2, 115.9, 109.1, 101.8, 60.6, 52.5, 52.4, 51.1, 24.6.
HRMS (ESI) calcd for $C_{20}H_{16}Cl_2N_3O_2$ [MH]+ 400.0614, found 400.0621.

Ester hydrolysis

Using General Method D, trans-ester (295mg, 0.27 mmol) was dissolved in THF / MeOH / H2O (0.7 mL / 0.7 mL / 0.7 mL) and Amberlyst hydroxide resin (3.10 g, Aldrich, loading: 4.2 mmol/g) was added. After 24 h, workup afforded a residue to which was added MeOH (0.5 mL), followed by addition of Et_2O and hexane. The mixture was stirred for 15 minutes in ice bath and then filtered. The solid was washed with hexane to afford desired product (131 mg, 46%), $[\alpha]_D^{23}$=-46.1 (c=5.8, MeOH).
^{1}H NMR (500 MHz, CD_3OD) δ 7.68 (d, *J* = 2.1 Hz, 1H), 7.57 (dd, *J* = 8.2, 0.8 Hz, 1H), 7.46 (d, *J* = 7.4 Hz, 1H), 7.34 (dd, *J* = 8.4, 2.1 Hz, 1H), 7.24 (t, *J* = 7.8 Hz, 1H), 6.98 (dd, *J* = 8.4, 2.3 Hz, 1H), 6.32 (d, *J* = 3.6 Hz, 1H), 3.94 (dd, *J* = 7.9, 5.2 Hz, 1H), 3.70 (dd, *J* = 16.3, 5.3 Hz, 1H), 3.47 (dd, *J* = 16.6, 8.3 Hz, 1H).
^{13}C NMR (126 MHz, CD_3OD) δ 176.2, 138.5, 136.9, 136.7, 135.4, 133.8, 133.1, 131.0, 128.7, 127.4, 126.5, 123.0, 120.0, 117.6, 109.9, 102.2, 54.5, 52.6, 25.1.
HRMS (ESI) calcd for $C_{19}H_{14}Cl_2N_3O_2$ [MH]+ 386.0458, found 386.0441.

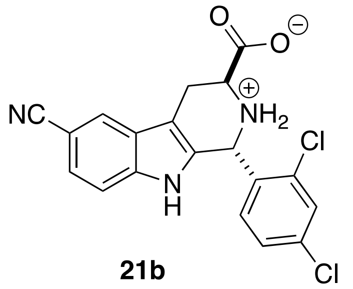

21b

(1*R*,3*S*)-1-(2,4-dichlorophenyl)-6-cyano-2,3,4,9-tetrahydro-1*H*-pyrido[3,4-*b*]indol-2-ium-3-carboxylate 21b (PRC1598, HL)

Synthesis of *N*-Boc-5-cyano-3-iodoindole **24b**

Using General Method E, 1*H*-indole-5-carbonitrile (1 g, 7.03 mmol), iodine (2.7 g, 10.6 mmol) and KOH (789 mg, 14.1 mmol.) were added in 15 mL of DMF. It was stirred at room temperature for 16 hours. When TLC shows no starting material left, 10 mL sat. $Na_2S_2O_3$ solution and 15 mL of water were added at 0 °C. It was stirred at room temperature for 15 min, the precipitate was collected by filtration. Without any purification, the crude product was re-dissolved in 15 mL DCM, di-*tert*-butyl decarbonate (1.69 g, 7.74 mmol) and *N,N*-dimethylaminopyridine (4-DMAP) (43 mg, 5 mol%) were added. The reaction was stirred at room temperature for 16 h. When TLC shows no starting material left, 10 mL 1M HCl solution was added. The separated organic layer was dried over $MgSO_4$ and concentra ted *in vacuo*. The residue was purified by silica chromatography (Hexane: EtOAc=6:1) to give 2.2 g of the desired product. (85%)

^{1}H NMR (500 MHz, $CDCl_3$) δ 8.24 (d, *J* = 8.6 Hz, 1H), 7.82 (s, 1H), 7.75 (d, *J* = 1.6 Hz, 1H), 7.61 (dd, *J* = 8.6, 1.6 Hz, 1H), 1.67 (s, 9H).

^{13}C NMR (126 MHz, $CDCl_3$) δ 148.1, 137.1, 132.6, 128.5, 126.7, 119.4, 116.2, 107.1, 85.8, 64.5, 28.2, 21.2.

HRMS (ESI) calcd for $C_{14}H_{14}IN_2O_2$ [MH]+ 369.0094, found 369.0079.

Synthesis of (*S*)-5-cyanotryptophan methyl ester hydrochloride **30b**

Using General Method G, to an oven dried Schlenk flask was charged with Zinc powder (400 mg, 6.1 mmol). The air was removed *in vacuo* and N_2 was purged for 2 times. Under N_2 protection, 2 mL of dry DMF was added. Under positive N_2 pressure, a portion of I_2 (26 mg, 0.1 mmol) was added. When the yellow color disappears, methyl (*R*)-2-((*tert*-butoxycarbonyl)amino)-3-iodopropanoate (670 mg, 2.04 mmol, 1 equiv.) was added followed by another portion of I_2 (26 mg, 0.2 mmol). The mixture was stirred at room temperature for 10 min, then *tert*-butyl 5-cyano-3-iodo-1*H*-indole-1-carboxylate (970 g, 2.63 mmol, 1.3 equiv.), $Pd_2(dba)_3$ (47 mg, 0.051 mmol) and SPhos (50 mg, 0.12 mmol) were sequentially added. It was stirred at room temperature for 20 hours. The reaction mixture was filtered to remove the Zinc residue and the filtrate was diluted with 15 mL of water and extracted with 10 mL of EtOAc for 3 times. The combined EtOAc layer were dried over $MgSO_4$ and concentrated *in vacuo*. The residue was purified by column with Hexane:EtOAc=20:1 to give Boc protected tryptophan methyl ester (0.33 g, 0.73 mmol, 36%). Using General Method H, the tryptophan methyl ester (40 mg, 0.09 mmol) was dissolved in 2 mL of 1M HCl in EtOAc. It was stirred at room temperature for 36

hours. The precipitate was collected by filtration to give **30b** (18 mg, 82%). $[\alpha]_D^{23}$=40.2 (c=5.2, MeOH)
^{1}H NMR (500 MHz, CD_3OD) δ 8.02 (dd, *J* = 1.6, 0.7 Hz, 1H), 7.55 (dd, *J* = 8.5, 0.7 Hz, 1H), 7.45 (dd, *J* = 8.5, 1.5 Hz, 1H), 7.40 (s, 1H), 4.38 (dd, *J* = 7.1, 5.9 Hz, 1H), 3.81 (s, 3H), 3.47 (ddd, *J* = 15.3, 5.9, 0.8 Hz, 1H), 3.40 (ddd, *J* = 15.3, 7.2, 0.7 Hz, 1H).
^{13}C NMR (126 MHz, CD_3OD) δ 170.6, 140.1, 128.4, 128.3, 125.7, 125.0, 121.6, 113.9, 109.0, 103.2, 54.5, 53.7, 27.1.
HRMS (ESI) calcd for $C_{13}H_{14}N_3O_2$ [MH]+ 244.1081, found 244.1095.

<u>Isolation of *trans*-Pictet-Spengler ester</u>
Using General Method C2, the **30b** (250 mg, 0.89 mmol) was basified by 10 mL sat. $NaHCO_3$ solution and extracted with DCM. The organic portion was dried over $MgSO_4$ concentrated *in vacuo* to obtain the tryptophan methyl ester free base. This free base and 2,4-dichlorobenzaldehyde (200 mg, 1.16 mmol) were added in a round bottle flask. Under N_2 protection, 0.5 mL of Ti(O*i*Pr)$_4$ were added and the reaction was stirred at 70°C for 16 hours. The reaction mixture was cooled down to 0°C then TFA (5mL, 35.5 mmol), TFAA (0.5mL, 6.62mmol) were added. It was stirred at 70 °C for two more days. The reaction was cooled down to room temperature and pulled into a 50 mL beaker. The pH was adjusted to pH=8 by the addition of sat. $NaHCO_3$ solution. 20 mL of DCM and 10 mL of water were added. The precipitate was removed by filtration and the organic portion was separated, and the water portion was extracted with DCM 10 mL for 3 times. The organic portions were combined, dried over $MgSO_4$ and concentrated *in vacuo*. The *trans* product was isolated by silica chromatography (Hexane; DCM: EtOAc=10:10:1) affording 90 mg (24%). $[\alpha]_D^{23}$=-17.2 (c=1.8, MeOH)

^{1}H NMR (400 MHz, $CDCl_3$) δ 8.42 (s, 1H), 7.93 – 7.86 (m, 1H), 7.47 (d, *J* = 2.1 Hz, 1H), 7.40 (dd, *J* = 8.4, 1.6 Hz, 1H), 7.30 (dd, *J* = 8.4, 0.7 Hz, 1H), 7.14 (dd, *J* = 8.4, 2.1 Hz, 1H), 6.93 (d, *J* = 8.3 Hz, 1H), 5.89 (s, 1H), 5.04 (p, *J* = 6.3 Hz, 1H), 3.85 – 3.81 (m, 1H), 3.23 (dd, *J* = 15.3, 5.2 Hz, 1H), 3.09 (dd, *J* = 15.5, 7.1 Hz, 1H), 1.23 (dd, *J* = 13.0, 6.2 Hz, 6H).
^{13}C NMR (126 MHz, $CDCl_3$) δ 172.3, 137.9, 137.1, 134.8, 134.3, 134.2, 130.8, 129.9, 127.5, 126.8, 125.4, 123.9, 120.6, 111.8, 110.3, 103.0, 69.1, 52.4, 50.9, 24.2, 21.8.
HRMS (ESI) calcd for $C_{22}H_{20}Cl_2N_3O_2$ [MH]+ 428.0927, found 428.0927.

<u>Ester hydrolysis</u>
Using General Method D, *trans*-ester (82 mg, 0.21 mmol) was dissolved in THF / MeOH / H2O (1 mL / 1 mL / 1 mL) and Amberlyst hydroxide resin (830 mg, Aldrich, loading: 4.2 mmol/g) was added. After 24 h, workup afforded a residue to which was added MeOH (0.5 mL), followed by addition of Et_2O and hexane. The mixture was stirred for 15 minutes in ice bath and then filtered. The solid was washed with hexane to afford desired product (16 mg, 20%), $[\alpha]_D^{23}$=22.3 (c=4.3, MeOH).
^{1}H NMR (500 MHz, CD_3OD) δ 8.03 (s, 1H), 7.70 (d, *J* = 2.0 Hz, 1H), 7.47 – 7.37 (m, 2H), 7.35 (dd, *J* = 8.4, 2.1 Hz, 1H), 7.00 (d, *J* = 8.3 Hz, 1H), 6.35 (s, 1H), 3.97 (t, *J* = 6.6 Hz, 1H), 3.45 (dd, *J* = 16.2, 5.1 Hz, 1H), 3.25 (dd, *J* = 16.7, 8.3 Hz, 1H).
^{13}C NMR (126 MHz, CD_3OD) δ 173.8, 140.4, 137.5, 137.1, 133.9, 133.3, 131.8, 131.1, 129.0, 127.4, 126.5, 125.2, 121.5, 113.5, 111.0, 103.3, 54.6, 52.3, 24.0.

HRMS (ESI) calcd for $C_{19}H_{14}Cl_2N_3O_2$ [MH]+ 386.0458, found 386.0456.

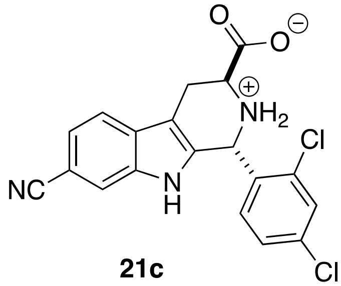

21c

(1*R*,3*S*)-1-(2,4-dichlorophenyl)-7-cyano-2,3,4,9-tetrahydro-1*H*-pyrido[3,4-*b*]indol-2-ium-3-carboxylate 21c (PRC1558, HL)

Synthesis of *N*-Boc-6-cyano-3-iodoindole **24c**

Using General Method E, 1*H*-indole-6-carbonitrile (1.1 g, 7.74 mmol), iodine (2.95 g, 11.6 mmol) and KOH (870 mg, 15.5 mmol.) were added in 15 mL of DMF. It was stirred at room temperature for 16 hours. When TLC indicated no starting material was left, 10 mL sat. $Na_2S_2O_3$ solution and 15 mL of water were added at 0 °C. It was stirred at room temperature for 15 min, the precipitate was collected by filtration. Without any purification, the crude product was re-dissolved in 15 mL DCM, di-*tert*-butyl decarbonate (1.86 g, 8.51 mmol) and *N,N*-dimethylpyridin-4-amine (4-DMAP) (47 mg, 5 mol%) were added. The reaction was stirred at room temperature for 16 h. When TLC indicated no starting was material left, 10 mL 1M HCl solution was added. The separated organic layer was dried over $MgSO_4$ and concentrated *in vacuo*. The residue was purified by silica chromatography (Hexane: EtOAc=6:1) to give 2.33 g of **24c**, 82%.

^{1}H NMR (400 MHz, $CDCl_3$) δ 8.54 – 8.48 (s, 1H), 7.89 (s, 1H), 7.56 (dd, *J* = 8.2, 1.4 Hz, 1H), 7.49 (dd, *J* = 8.2, 0.7 Hz, 1H), 1.68 (s, 9H).

^{13}C NMR (101 MHz, $CDCl_3$) δ 148.1, 135.5, 134.1, 133.5, 126.5, 122.6, 119.8, 119.7, 108.6, 85.9, 64.8, 28.2.

HRMS (ESI) calcd for $C_{14}H_{14}IN_2O_2$ [MH]+ 369.0094, found 369.0102.

Synthesis of (*S*)-6-cyanotryptophan methyl ester hydrochloride **30c**

Using General Method G, to an oven dried Schlenk flask was charged with Zinc powder (1.24 g, 19 mmol). The air was removed *in vacuo* and N_2 was purged for 2 times. Under N_2 protection, 6 mL of dry DMF was added. Under positive N_2 pressure, a portion of I_2 (80 mg, 0.3 mmol) was added. When the yellow color disappears, methyl (*R*)-2-((*tert*-butoxycarbonyl)amino)-3-iodopropanoate (2.08 g, 6.33 mmol, 1 equiv.) was added followed by another portion of I_2 (80 mg, 0.3 mmol). The mixture was stirred at room temperature for 10 min, then *tert*-butyl 6-cyano-3-iodo-1*H*-indole-1-carboxylate (2.33 g, 6.33 mmol), $Pd_2(dba)_3$ (145 mg, 0.16 mmol) and SPhos (156 mg, 0.38 mmol) were sequentially added. It was stirred at room temperature for 20 hours. The reaction mixture was filtered to remove the Zinc residue and the filtrate was diluted with 20 mL of water and extracted with 20 mL of EtOAc for 3 times. The combined EtOAc layer was dried over $MgSO_4$ and concentrated *in vacuo*. The residue was purified by column with Hexane:EtOAc=20:1 to give Boc protected tryptophan methyl ester (1.05 g, 2.37 mmol, 37%).

Following general method H, the tryptophan methyl ester (0.75 g, 1.69 mmol) was dissolved in 15 mL of 1M HCl in EtOAc. It was stirred at room temperature for 48 hours. The precipitate was collected by filtration to give **30c** (397 mg, 94%). $[\alpha]_D^{23}$=23.2 (c=5.6, MeOH)

^{1}H NMR (500 MHz, CD_3OD) δ 7.84 (dd, *J* = 1.4, 0.7 Hz, 1H), 7.72 (dd, *J* = 8.2, 0.8 Hz, 1H), 7.51 (s, 1H), 7.38 (dd, *J* = 8.3, 1.4 Hz, 1H), 4.38 (dd, *J* = 7.1, 5.8 Hz, 1H), 3.81 (s, 3H), 3.49 (ddd, *J* = 15.2, 5.8, 0.8 Hz, 1H), 3.42 (dd, *J* = 15.3, 7.1 Hz, 1H).
^{13}C NMR (126 MHz, CD_3OD) δ 169.2, 135.7, 130.1, 128.7, 121.7, 120.0, 118.7, 116.3, 107.5, 103.7, 53.1, 52.3, 25.7.
HRMS (ESI) calcd for $C_{13}H_{14}N_3O_2$ [MH]+ 244.1081, found 244.1078.

Isolation of *trans*-Pictet-Spengler ester
The above tryptophan methyl ester hydrochloride (68 mg, 0.24 mmol), $MgSO_4$ and TEA (49 mg, 0.49 mmol) were combined in 0.5 mL of DCM. After stirring for 30 min, the $MgSO_4$ was removed with a syringe filter and the filtrate was concentrated *in vacuo*. The residue was dissolved in TFA (1 mL, 13.2 mmol), TFAA (0.1 mL, 0.7 mmol), 2,4-dichlorobenzaldehyde (54 mg, 0.31 mmol) was added, and heated to 70°C for another 8 hours. Note that Ti(O*i*-Pr)$_4$ was not included in this reaction, since it was found to competitively reduce the aldehyde to 2,4-dichlorobenzyl alcohol (Meerwein-Pondorf-Verley reaction). The mixture was cooled to 0°C and quenched by the addition of sat. $NaHCO_3$ solution to pH = 7. It was extracted with 10 mL EtOAc for 3 times. The organic portion was dried over $MgSO_4$ and concentrated *in vacuo*. The crude product was purified by silica chromatography (Hexane: DCM: EtOAc=10:10:1) to give *trans* isomer (16 mg, 4%). $[\alpha]_D^{23}$=7.74 (c=1.55, MeOH)

^{1}H NMR (500 MHz, $CDCl_3$) δ 7.96 (s, 1H), 7.64 – 7.60 (m, 2H), 7.53 (d, *J* = 2.1 Hz, 1H), 7.41 (dd, *J* = 8.1, 1.5 Hz, 1H), 7.19 (dd, *J* = 8.4, 2.1 Hz, 1H), 7.01 (d, *J* = 8.4 Hz, 1H), 5.95 (s, 1H), 3.95 (dd, *J* = 6.7, 5.2 Hz, 1H), 3.77 (s, 3H), 3.29 (ddd, *J* = 15.4, 5.2, 1.3 Hz, 1H), 3.17 (ddd, *J* = 15.4, 6.7, 1.5 Hz, 1H).
^{13}C NMR (126 MHz, $CDCl_3$) δ 173.5, 137.2, 136.1, 135.1, 135.0, 134.4, 130.9, 130.1, 127.7, 123.2, 120.6, 119.3, 115.8, 110.3, 104.9, 60.6, 52.5, 51.1, 24.4.
HRMS (ESI) calcd for $C_{20}H_{16}Cl_2N_3O_2$ [MH]+ 400.0614, found 400.0622.

Ester hydrolysis
Using General Method D, the *trans*-ester (83mg, 0.27 mmol) was dissolved in THF / MeOH / H2O (1 mL / 1 mL / 1 mL) and Amberlyst hydroxide resin (830 mg, Aldrich, loading: 4.2 mmol/g) was added. After 24 h, workup afforded a residue to which was added MeOH (0.5 mL), followed by addition of Et_2O and hexane. The mixture was stirred for 15 minutes in ice bath and then filtered. The solid was washed with hexane to afford desired product (13 mg, 16%). $[\alpha]_D^{23}$=3.75 (c=0.8, MeOH)

^{1}H NMR (500 MHz, CD_3OD) δ 7.77 – 7.66 (m, 3H), 7.42 – 7.32 (m, 2H), 7.01 (d, *J* = 8.4 Hz, 1H), 6.39 (s, 1H), 3.99 (dd, *J* = 7.8, 5.4 Hz, 1H), 3.46 (dd, *J* = 16.1, 5.3 Hz, 1H), 3.27 (dd, *J* = 15.8, 7.5 Hz, 1H).
^{13}C NMR (126 MHz, CD_3OD) δ 173.8, 137.5, 137.4, 137.1, 134.1, 133.7, 133.3, 131.1, 130.6, 129.1, 128.0, 123.4, 121.3, 117.2, 110.9, 105.7, 54.7, 52.4, 24.0.

HRMS (ESI) calcd for $C_{19}H_{14}Cl_2N_3O_2$ [MH]+ 386.0458, found 386.0453.

2. In vivo efficacy studies of 1 (NYU) in *P. berghei*-infected mice (po and iv)

Efficacy of **1** in *P. berghei*-infected mice was assessed at the Anti-Infectives Core Facility, Department of Microbiology, Grossman School of Medicine, New York University.

A. Oral Administration Protocol

Three groups of five female Swiss Webster mice weighing 25 to 30 g were used for vehicle control, **1** treatment (PO, 45 mg/kg/d) and the positive control chloroquine (PO, 45 mg/kg/d) groups. On Day 0, mice were infected via intraperitoneal (i.p.) injection with 10^3 *Plasmodium berghei* ANKA expressing luciferase (PbGFP-Luccon) obtained from a donor infected mouse.[10] As shown below in Figure S1, mice were treated orally with **1**, chloroquine, or vehicle from day 2 to 6 and imaged on day 7.

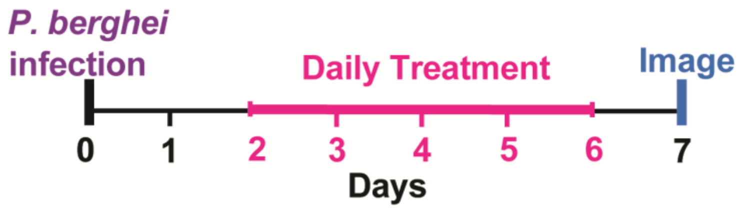

Figure S1. Dosing and imaging timeline of *P. berghei*-infected mice. Vehicle, **1**, and CQ treatments were administered orally once-daily on Days 2-6. Surviving mice were imaged on Day 7 as described in the text.

The vehicle for all groups was 0.5% (hydroxypropyl)methyl cellulose, 0.5% Tween-80. As seen in Table S1 below, by Day 7, 3 of the 5 vehicle-treated mice were dead, and 4 of the **1**-treated mice were dead. All 5 of the CQ-treated mice survived. For the surviving mice, on day 7 after infection, mice were anesthetized by inhalation of isofluorane and injected via i.p. with 150 mg/kg of luciferase substrate dissolved in PBS (D-Luciferin Potassium Salt, Gold Biotechnology). Mice were imaged 5 to 10 min after injection of luciferin using an IVIS imager (Lumina II In Vivo Imaging System; Perkin-Elmer). The data acquisition and analysis were performed with LivingImage (Xenogen). Accumulated light intensity was measured in each mouse to determine the baseline infection levels before treatment.

Table S1. Live/Dead and Luminescence Data for Oral dosing of Vehicle, **1**, and Chloroquine (CQ)

	**Vehicle**	**Cmpd 1 45 mg/kg 1 x daily – PO**	**CQ 20 mg/kg 1 x daily – PO**
	Dead by Day 7	Dead by Day 6	4.72E+03

	Dead by Day 7	Dead by Day 7	4.45E+03
	Dead by Day 7	Dead by Day 7	4.90E+03
	4.82E+07	1.33E+06	4.60E+03
	5.23E+05	Dead by Day 6	4.61E+03
**AVG**	2.44E+07	---	4.65E+03
**SD**	2.38E+07	---	1.66E+02

B. IV Administration Protocol

One group of four female Swiss Webster mice weighing 25 to 30 g were used for vehicle control, and groups of five mice were used for **1** treatment (IV, 2 x 30 mg/kg/d) and the positive control chloroquine (ip, 20 mg/kg/d). On Day 0, mice were infected via intraperitoneal (i.p.) injection with 10^3 *Plasmodium berghei* ANKA expressing luciferase (PbGFP-Luccon) obtained from a donor infected mouse.[10] As shown below in Figure S2 (and legend), mice were treated vehicle, **1**, or CQ on days 2 to 6 and imaged on day 7.

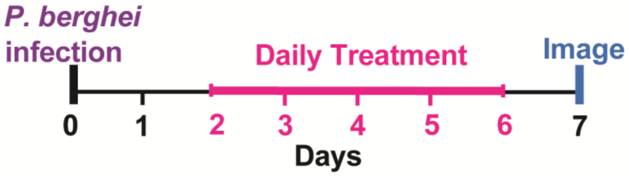

Figure S2. Dosing and imaging timeline of *P. berghei*-infected mice. Vehicle and **1** (30 mpk) treatments were administered IV twice-daily on Days 2-6. CQ (20 mpk) treatments were administered i.p. once-daily. Mice were imaged on Day 7, as described in the text.

The vehicle for this study was 30%PEG, 40% Captisol (30%w/v solution). Note that vehicle-control mouse survivorship was improved for this study, with all four vehicle-control mice surviving to Day 7. Luminescence data are given in Table S2.

Table S2. Luminescence Data after background subtraction for IV dosing of Vehicle, **1**, and IP dosing of Chloroquine (CQ). This data is plotted in Figure S3.

	**Vehicle**	**Cmpd 1 30 mg/kg 2 x daily – IV**	**CQ 20 mg/kg 1 x daily – i.p.**
	2.61E+06	9.49E+05	4.64E+03
	4.07E+06	1.50E+06	2.76E+03
	9.85E+05	1.72E+06	3.48E+03
	1.04E+06	2.00E+06	5.47E+02
		7.68E+05	0.00E+00
**AVG**	2.18E+06	1.39E+06	2.29E+03
**SD**	1.47E+06	5.18E+05	1.96E+03

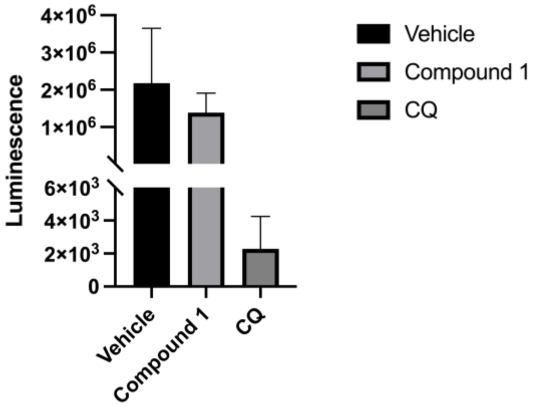

Figure S3. Luminescence after background subtraction of *P. berghei*-infected mice on Day 7, following treatment with vehicle (IV, twice daily IV, Days 2-6), and **1** (IV, twice daily IV 30 mpk, Days 2-6), CQ (IP, once daily, 20 mpk, Days 2-6)). Data was taken from Table S2.

3. Pharmacokinetics of 1 in mice

Pharmacokinetic analysis of **1** in mice was performed by Pharmaron (Beijing, China). Three mice each (23 ± 1 g) were used for oral (40 mg/kg, gavage, conscious) and for IV (10 mg/kg, lateral tail vein with anesthesia) administration. The vehicle for both routes was 20% DMSO, 60% PEG400 and 20% SBE-β-CD (30% w/v) in water. Stock concentrations were 4 mg/mL (both oral and IV respectively), and mice were dosed at 10.0 and 2.5 mL/kg (oral and IV respectively). Quantitation was performed by triple-quad LC-MS. Key pharmacokinetic parameters are shown in Table S3. Concentration vs time data are shown in Table S4 and Figure S4.

Table S3. Key noncompartmental pharmacokinetic (mouse) parameters of **1**.

Route (dose)	Parameter	Value for **1**
IV (10 mg/kg)	$t_{1/2}$ (h)[a]	9.9 ± 4
	CL (mL/min/kg)	12.1 ± 1.3
	C_{max} (μM)	---
	C_0 (μM)	86.0 ± 6.6
	t_{last} (h)	24
	C_{min} (μM)	0.164 ± 0.017
	C_{max}/C_{min}	524
	$AUC_{0\text{-}24h}$ (h•μM)	36.1 ± 3.6
	$AUC_{0\text{-}inf}$ (h•μM)	38.4 ± 3.8
	V_d (L/kg)	3.5 ± 0.43
PO (40 mg/kg)	$t_{1/2}$ (h)[a]	8.5 ± 0.24
	t_{max} (h)	0.25
	C_{max} (μM)	46.1 ± 7.3
	t_{last} (h)	24
	C_{min} (μM)	0.65 ± 0.35
	C_{max}/C_{min}	71
	$AUC_{0\text{-}24h}$ (h•μM)	79.0 ± 8.9
	$AUC_{0\text{-}inf}$ (h•μM)	88 ± 15
	%F	57 ± 10

[a]Half-life values reflect the elimination phase only (4-24 h).

Table S4. Mean concentrations of **1** in plasma following IV and PO dosing.

time (h)	IV (10 mg/kg) [**1**] (μM)[a]	PO (40 mg/kg) [**1**] (μM)[a]
0.083	58.6 ± 5.5	nd
0.25	27.1 ± 3.7	46.1 ± 8.0
0.5	14.8 ± 1.9	28.2 ± 4.1
1	4.8 ± 1.7	12.7 ± 2.7
2	1.3 ± 0.4	5.4 ± 0.31
4	0.66 ± 0.04	3.2 ± 0.25
8	0.51 ± 0.03	2.4 ± 0.45
24	0.16 ± 0.02	0.64 ± 0.35

[a]Average of three mice

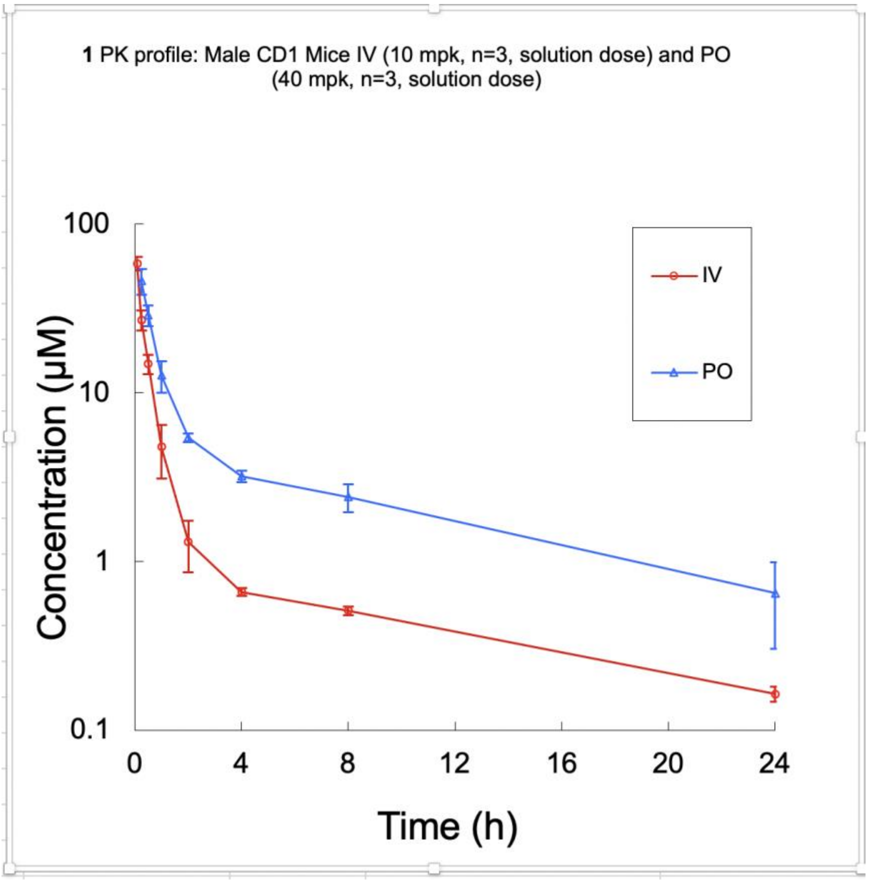

Figure S4. Plasma concentration of **1** vs time in mice, following oral (40 mg/kg) and IV (10 mg/kg dosing.

4. Mouse microsomal stability of 1 (Pharmaron)

Metabolic stability of **1** was studied in pooled male mouse liver microsomes (final concentration 0.5 mg/mL, 5 mM $MgCl_2$, 100 mM phosphate buffer). The final concentration of test compounds or verapamil in the reaction system was 1 µM. The mixture was pre-warmed 37°C for 5 min before addition of 50 µL of 10 mM NADPH solution at the final concentration of 1 mM and carried out at 37°C. 50 µL of ultra-pure H_2O was used instead of NADPH solution in the negative control. Verapamil was used as a reference in the +NADPH experiment. Drug concentrations were determined using triple quad LC-MS (Table S5).

Table S5. Mouse microsomal stability of **1** in the presence and absence of NADPH.

Drug	+ NADPH<br>% remaining at 60 min	-NADPH<br>% remaining at 60 min
**1**	2.6	103.9
Verapamil	3.7	nd

Based on these data, **1** is unstable to P450-mediated oxidation in the liver. Applying first-order kinetics with only two points (0, 60 min), we can extrapolate an approximate $t_{1/2}$ of ~10 min.

5. Mouse microsomal stability of benzo-ring fluorinated analogs 20a, 20c, 20d

Mouse microsomal stability of **20a**, **20c**, **20d** was first assessed in our laboratory. Compounds **1** and coumarin were used as controls. Data are provided in Table S6. Microsomal stability was assessed by ultra-performance liquid chromatography and high-resolution mass spectrometry analysis (UPLC/MS/MS). The pooled female mouse liver microsomes (CD-1) (Cat. # 452702), NADPH Regenerating System - Solution A (Cat. # 451220), and NADPH Regenerating System - Solution B (Cat. # 451200) were purchased from BD Biosciences - Discovery Labware (Bedford, MA). Potassium phosphate monobasic, potassium phosphate dibasic, and coumarin were purchased from Sigma-Aldrich (St. Louis, MO). Briefly, compound incubations were carried out by the addition of mouse liver microsomes (0.5 mg/ml) in 0.05 M potassium phosphate buffer (pH 7.4), solution A and B, and preincubated at 37°C for 5 min. Control experiments were also carried out in the absence of NADPH solution A and B. Coumarin reaction was used as positive control. Reactions were terminated by adding acetonitrile. Sample analysis was performed on an IonKey/MS system composed of an ACQUITY UPLC M-Class, the ionKey source, and an iKey BEH C18 130 Å 1.7 µm 150 µm x 50 mm column coupled to a Synapt G2-Si mass spectrometer (Waters Corporation, Milford, MA, USA). Separation was accomplished using a binary gradient system consisting of 0.1% formic acid in water (mobile phase A) and 0.1% formic acid in acetonitrile (mobile phase B). The linear gradient was programmed as follows: 0-3 min 1% B, 3-4 min from 1% B to 99% B, 4-5 min back to 1% B, and re-equilibrated for 9 min. The flow rate was 3 µL/min, the injection volume was 3 µL in full loop mode, and the iKey temperature was set at 40°C. MS analyses were performed acquiring in MSE mode from 80 to 1500 m/z in positive electrospray ionization mode with a capillary voltage of 3 kV. Data were collected in two channels all the time: low collision energy (6.0 V), for the molecular ions, and high collision energy (15-40 V), for product ions. The source temperature was set at 110°C. Leucine enkephalin (50 pg/mL) was used as the lock mass (m/z 556.2771) with parameters set to a 1 s scan at 20 s intervals. Infusion flow rate for lock mass was 1 µL/min. The microsomal stability was determined using MassLynx software (Waters Corporation, Milford, MA, USA). Relative amounts were calculated by comparing the peak area of each evaluated compound at time 0 minutes to its corresponding area at times 45, 90 and 180 minutes to return a ratio.

Table S6. Mouse microsomal stability of 1 and fluorinated analogs in the presence of NADPH.

Compound	% drug remaining			
	0 min	45 min	90 min	180 min
coumarin	100	27	5	-
**1**	100	3	1	-
**20a**	100	6	2	-
**20c**	100	96	95	64
**20d**	100	84	68	57

As can be seen, the stability of **1** in the presence of liver microsomes in the presence of NADPH (3% remaining at 45 min) was very similar to that reported by Pharmaon (2.6% remaining at 60 min). Note that **20c** and **20d** showed greater stability than **1** in mouse liver microsomes. For confirmation, these compounds were evaluated at Pharmaron (Table 7, below).

Table S7. Mouse microsomal stability of fluorinated analogs in the presence of NADPH.

compound		% remaining						$t_{1/2}$ (min)
		0.5 min	5 min	10 min	15 min	20 min	30 min	
Verapamil	Replicate 1	100	19.1	5.7	2.4	1.3	1.0	
	Replicate 2	100	19.6	5.8	2.8	1.4	0.7	
	Mean	100	19.4	5.8	2.6	1.4	0.9	1.9
**20c**	Replicate 1	100	105.2	105.5	100.5	100.4	95.2	
	Replicate 2	100	90.9	91.4	84.7	85.9	85.7	
	Mean	100	98.1	98.5	92.6	93.2	90.4	214
**20d**	Replicate 1	100	56.5	41.8	31.5	28.2	24.8	
	Replicate 2	100	61.1	42.6	34.3	30.3	25.1	
	Mean	100	58.8	42.2	32.9	29.3	25.0	16

6. Pharmacokinetics of 20c in mice (Virginia Tech)

Pharmacokinetic analysis of **20c** was performed at the Analytical Chemistry Research Laboratory at the Virginia-Maryland College of Veterinary Medicine. The study design included a naïve average sampling approach. Twenty-one mice (32.2 ± 1.67 g) were used for oral (40 mg/kg, gavage, conscious) and 24 mice (32.2 ± 1.91 g) were used for IV (10 mg/kg, lateral tail vein, conscious) administration. Three mice were then euthanized at each timepoint using a standard CO_2 protocol and blood was collected via cardiac puncture. Timepoints for sampling were 0.083 (IV only), 0.25, 0.5, 1, 2, 4, 8 and 24 hours. The vehicle for both routes was 40% PEG400 and 60% PBS made up to a stock concentration of 4 mg/mL. Mice were dosed at 10.0

and 2.5 mL/kg (oral and IV respectively). Quantitation was performed by UPLC-MS/MS. Key pharmacokinetic parameters for **20c** are shown in Table S8.

Table S8. Key noncompartmental pharmacokinetic (mouse) parameters of **20c**.

Route (dose)	Parameter	Value for **20c**
IV (10 mg/kg)	$t_{1/2}$ (h)[a]	4.96
	CL (mL/min/kg)	8.85
	C_{max} (µM)	---
	C_0 (µM)	34.5
	t_{last} (h)	24
	C_{min} (µM)	0.1 ± 0.1
	C_{max}/C_{min}	345
	$AUC_{0\text{-}24h}$ (h•µM)	48.7
	$AUC_{0\text{-}inf}$ (h•µM)	49.6
	V_d (L/kg)	3.8
PO (40 mg/kg)	$t_{1/2}$ (h)[a]	5.17
	t_{max} (h)	0.5
	C_{max} (µM)	19.9 ± 8.1
	t_{last} (h)	24
	C_{min} (µM)	0.6 ± 0.6
	C_{max}/C_{min}	33.2
	$AUC_{0\text{-}24h}$ (h•µM)	110.1
	$AUC_{0\text{-}inf}$ (h•µM)	114
	%F	57.8

[a]Half-life values reflect the elimination phase only (4-24 h).

Table S9. Concentration of **20c** vs time in mice.

Time (h)	IV (10 mg/kg) conc (µM)	PO (40 mg/kg) conc (µM)
0.0833	33.3	na
0.25	30.9 ± 4.0	19.8 ± 8.1
0.5	14.4 ± 4.1	19.9 ± 3.2
1	9.7 ± 2.0	13.9 ± 10.8
2	3.3 ± 1.5	8.8 ± 5.9
4	1.4 ± 0.4	10.0 ± 3.7
8	1.4 ± 0.3	3.9 ± 0.7
24	0.1 ± 0.1	0.6 ± 0.06

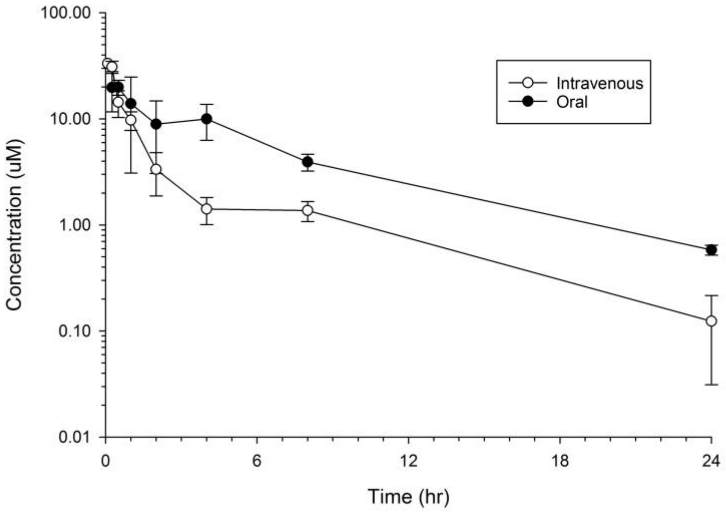

Figure S5. Plasma concentration of **20c** vs time in mice, following oral (40 mg/kg) and IV (10 mg/kg dosing.

7. Antimalarial Efficacy of 20c in *P. berghei*-infected mice (NYU)

Efficacy of **20c** in *P. berghei*-infected mice was assessed at the Anti-Infectives Core Facility, Department of Microbiology, Grossman School of Medicine, New York University.

A. Oral Administration Protocol

Three groups of five female Swiss Webster mice weighing 25 to 30 g were used for vehicle control, **20c** treatment (PO, 40 mg/kg/d) and the positive control chloroquine (PO, 40 mg/kg/d) groups. On Day 0, mice were infected via intraperitoneal (i.p.) injection with 10^3 *Plasmodium berghei* ANKA expressing luciferase (PbGFP-Luccon) obtained from a donor infected mouse.[10]

As shown below in Figure S6, mice were treated orally with **20c**, chloroquine, or vehicle from day 2 to 6 and imaged on day 7.

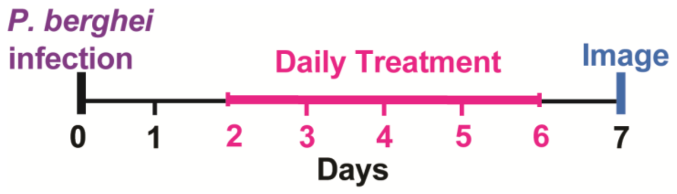

Figure S6. Dosing and imaging timeline of *P. berghei*-infected mice. Vehicle, **20c**, and CQ treatments were administered orally once-daily on Days 2-6. Surviving mice were imaged on Day 7 as described in the text.

The vehicle for all groups was 40% PEG-400 and 60% PBS. All mice survived the *P.berghei* infection and drug treatment out to 7 days after infection. At this point mice were anesthetized by inhalation of isofluorane and injected via i.p. with 150 mg/kg of luciferase substrate dissolved in PBS (D-Luciferin Potassium Salt, Gold Biotechnology). Mice were imaged 5 to 10 min after injection of luciferin using an IVIS imager (Lumina II In Vivo Imaging System; Perkin-Elmer). The data acquisition and analysis were performed with LivingImage (Xenogen). Accumulated light intensity was measured in each mouse to determine the baseline infection levels before treatment.

Table S10. Luminescence Data after background subtraction for Oral dosing of Vehicle, **20c**, and Chloroquine (CQ).

	**Vehicle**	**Cmpd 20c 40 mg/kg 1 x daily - PO**	**CQ 40 mg/kg 1 x daily – PO**
	2.56E+06	6.51E+05	4.90E+03
	3.91E+05	2.35E+06	3.85E+03
	8.88E+04	3.10E+06	4.20E+03
	4.87E+05	4.05E+05	
	7.59E+05	1.33E+06	
**AVG**	5.43E+06	9.69E+06	1.58E+04
**SD**	5.54E+06	6.48E+06	3.47E+03

As can be seen, treatment with CQ (40 mg/kg x 5 d, po) reduced luminescence 97% relative to vehicle control, from 5.4E+05 ± 5.5E+05 to 1.6E+04 ± 3.5E±03. In contrast treatment with **20c** (40 mg/kg x 4 d, po) did not reduce luminescence; in fact, luminescence increased in the treated mice, from 5.4E+05 ± 5.5E+05 to 9.7E+06 to 6.8E+06. This result, though unfavorable, is consistent with the similar plasma exposures of **20c** and **1**, and the lower *in vitro* potency of **20c** relative to **1**.

8. References

1. Konda-Yamada, Y.; Okada, C.; Yoshida, K.; Umeda, Y.; Arima, S.; Sato, N.; Kai, T.; Takayanagi, H.; Harigaya, Y. Convenient synthesis of 7′ and 6′-bromo-D-tryptophan and their derivatives by enzymatic optical resolution using D-aminoacylase. *Tetrahedron* **2002,** *58*, 7851-7861.
2. Yokoyama, Y.; Hikawa, H.; Mitsuhashi, M.; Uyama, A.; Hiroki, Y.; Murakami, Y. Total Synthesis without Protection: Three-Step Synthesis of Optically Active Clavicipitic Acids by a Biomimetic Route. *European J. Org. Chem.* **2004,** *2004*, 1244-1253.
3. Yao, Z.-K.; Krai, P. M.; Merino, E. F.; Simpson, M. E.; Slebodnick, C.; Cassera, M. B.; Carlier, P. R. Determination of the active stereoisomer of the MEP pathway-targeting antimalarial agent MMV008138, and initial structure-activity studies. *Bioorg. Med. Chem. Lett.* **2015,** *25*, 1515-1519.
4. Ghavami, M.; Merino, E. F.; Yao, Z.-K.; Elahi, R.; Simpson, M. E.; Fernández-Murga, M. L.; Butler, J. H.; Casasanta, M. A.; Krai, P. M.; Totrov, M. M.; Slade, D. J.; Carlier, P. R.; Cassera, M. B. Biological Studies and Target Engagement of the 2-C-Methyl-*D*-Erythritol 4-Phosphate Cytidylyltransferase (IspD)-Targeting Antimalarial Agent (1*R*,3*S*)-MMV008138 and Analogs. *ACS Infect. Dis.* **2018,** *4*, 549-559.
5. Cagašová, K.; Ghavami, M.; Yao, Z.-K.; Carlier, P. R. Questioning the γ-gauche effect: stereoassignment of 1,3-disubstituted-tetrahydro-β-carbolines using 1H–1H coupling constants. *Org. Biomol. Chem.* **2019,** *17*, 6687-6698.
6. Horiguchi, Y.; Nakamura, M.; Saitoh, T.; Sano, T. A Syntheis of Chiral 1,1,3-Trisubstituted 1,2,3,4-Tetrahydro-β-carbolines by the Pictet-Spengler Reaction of Tryptophan and Ketones: Conversion of (1*R*,3*S*)-Diastereomers into their (1*S*,3*S*)-Counterparts by Scission of the C(1)-N(2) bond. *Chem. Pharm. Bull.* **2003,** *51*, 1368-1373.
7. Dandapani, S.; Lan, P.; Beeler, A. B.; Beischel, S.; Abbas, A.; Roth, B. L.; Porco, J. A.; Panek, J. S. Convergent Synthesis of Complex Diketopiperazines Derived from Pipecolic Acid Scaffolds and Parallel Screening against GPCR Targets. *J. Org. Chem.* **2006,** *71*, 8934-8945.
8. Lu, X.; Yi, J.; Zhang, Z. Q.; Dai, J. J.; Liu, J. H.; Xiao, B.; Fu, Y.; Liu, L. Expedient Synthesis of Chiral α-Amino Acids through Nickel-Catalyzed Reductive Cross-Coupling. *Chemistry-A European Journal* **2014,** *20*, 15339-15343.
9. Ross, A. J.; Lang, H. L.; Jackson, R. F. W. Much Improved Conditions for the Negishi Cross-Coupling of Iodoalanine Derived Zinc Reagents with Aryl Halides. *J. Org. Chem.* **2010,** *75*, 245-248.
10. Franke-Fayard, B.; Janse, C. J.; Cunha-Rodrigues, M.; Ramesar, J.; Büscher, P.; Que, I.; Löwik, C.; Voshol, P. J.; den Boer, M. A.; van Duinen, S. G.; Febbraio, M.; Mota, M. M.; Waters, A. P. Murine malaria parasite sequestration: CD36 is the major receptor, but cerebral pathology is unlinked to sequestration. *Proc Natl Acad Sci U S A* **2005,** *102*, 11468-73.

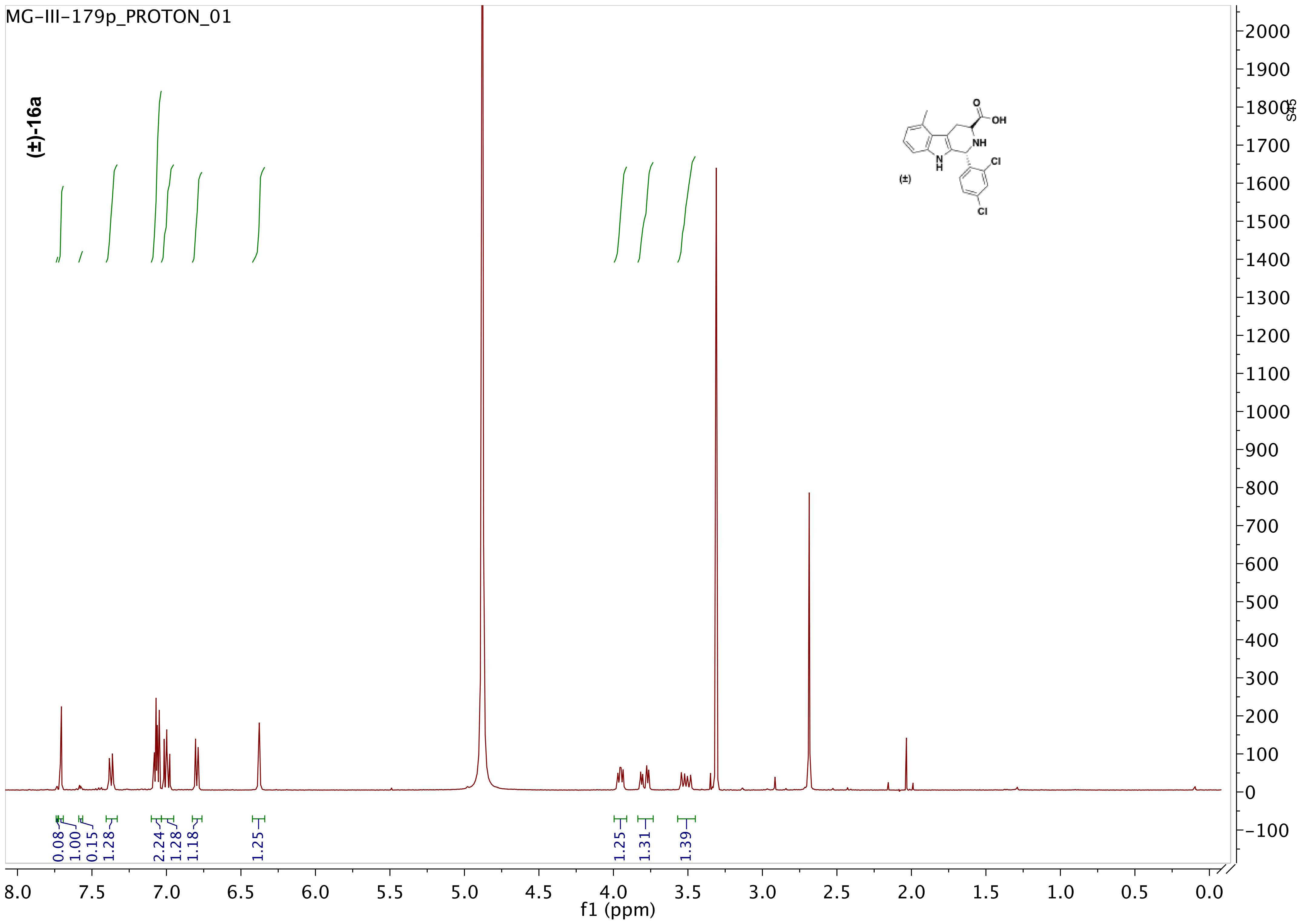
MG-III-179p_PROTON_01
(±)-16a
(±)
OH
NH
N
H
Cl
Cl
0.08
1.00
0.15
1.28
2.24
1.28
1.18
1.25
1.25
1.31
1.39
8.0
7.5
7.0
6.5
6.0
5.5
5.0
4.5
4.0
3.5
3.0
2.5
2.0
1.5
1.0
0.5
0.0
f1 (ppm)
2000
1900
1800
1700
1600
1500
1400
1300
1200
1100
1000
900
800
700
600
500
400
300
200
100
0
−100

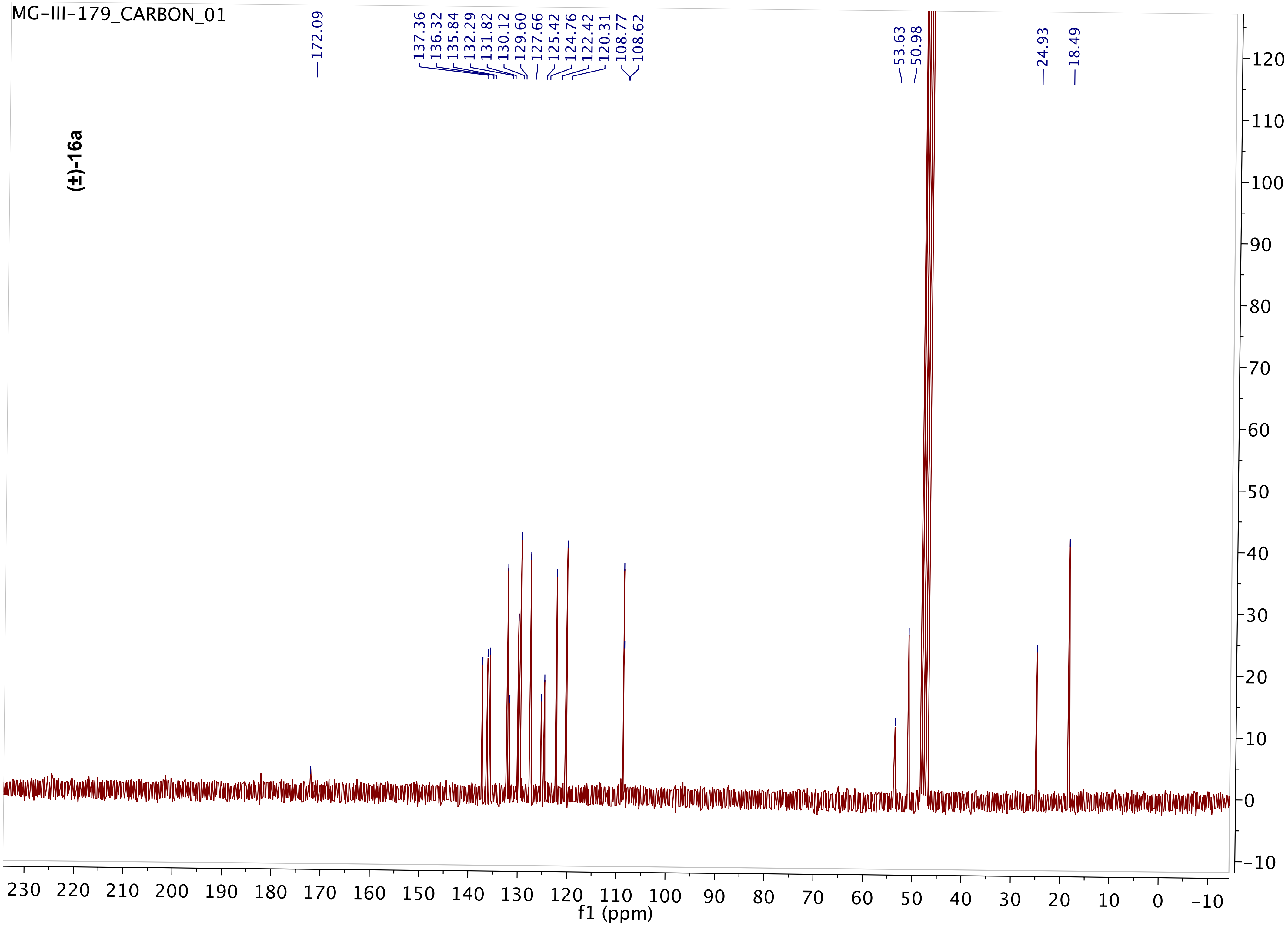

MG-III-179_CARBON_01
(±)-16a
172.09
137.36
136.32
135.84
132.29
131.82
130.12
129.60
127.66
125.42
124.76
122.42
120.31
108.77
108.62
53.63
50.98
24.93
18.49
230 220 210 200 190 180 170 160 150 140 130 120 110 100 90 80 70 60 50 40 30 20 10 0 -10
f1 (ppm)
-10 0 10 20 30 40 50 60 70 80 90 100 110 120

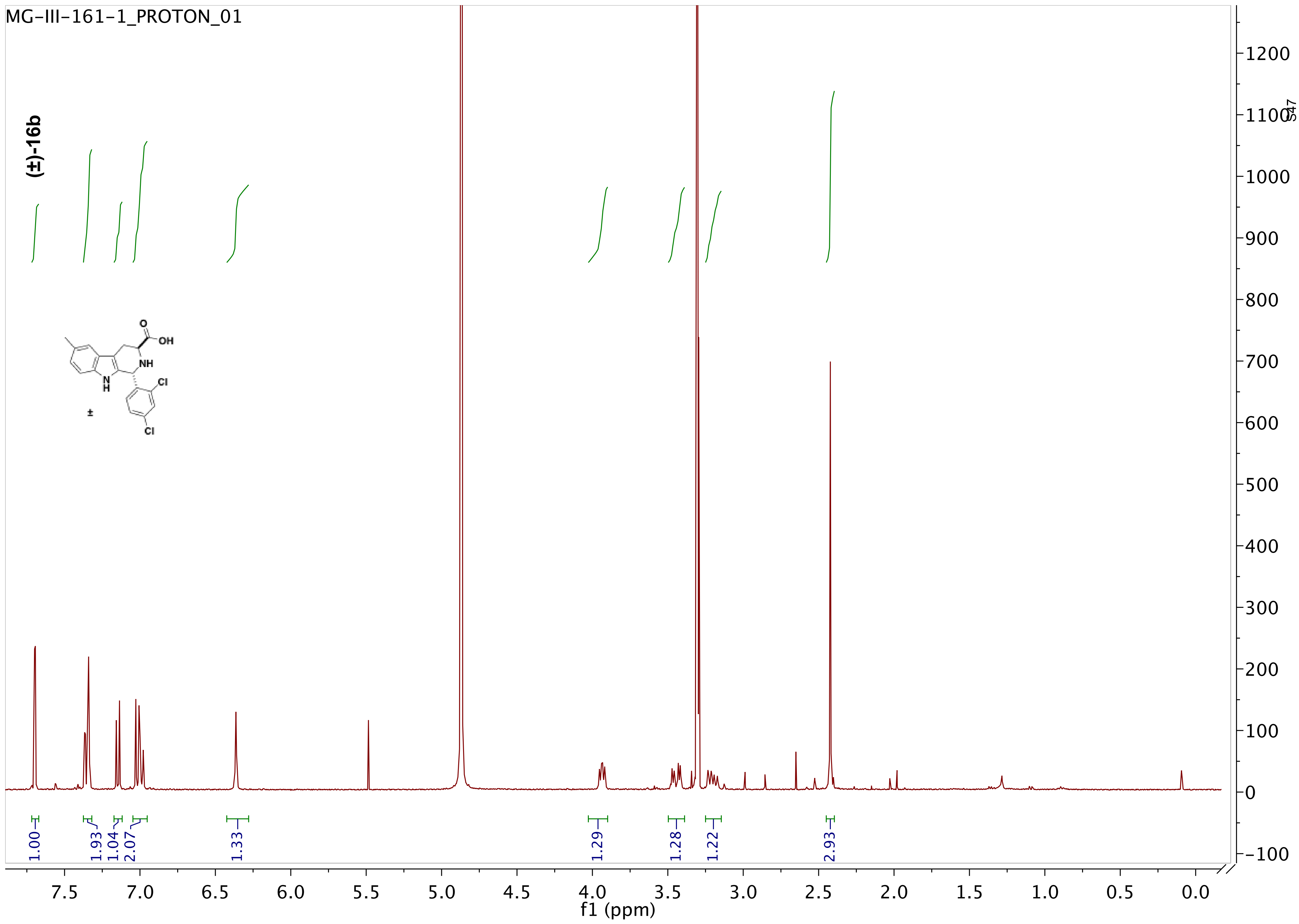
MG–III–161–1_PROTON_01
(±)-16b
O
OH
NH
N
H
Cl
Cl
±
1.00
1.93
1.04
2.07
1.33
1.29
1.28
1.22
2.93
7.5
7.0
6.5
6.0
5.5
5.0
4.5
4.0
3.5
3.0
2.5
2.0
1.5
1.0
0.5
0.0
f1 (ppm)
1200
1100
1000
900
800
700
600
500
400
300
200
100
0
–100

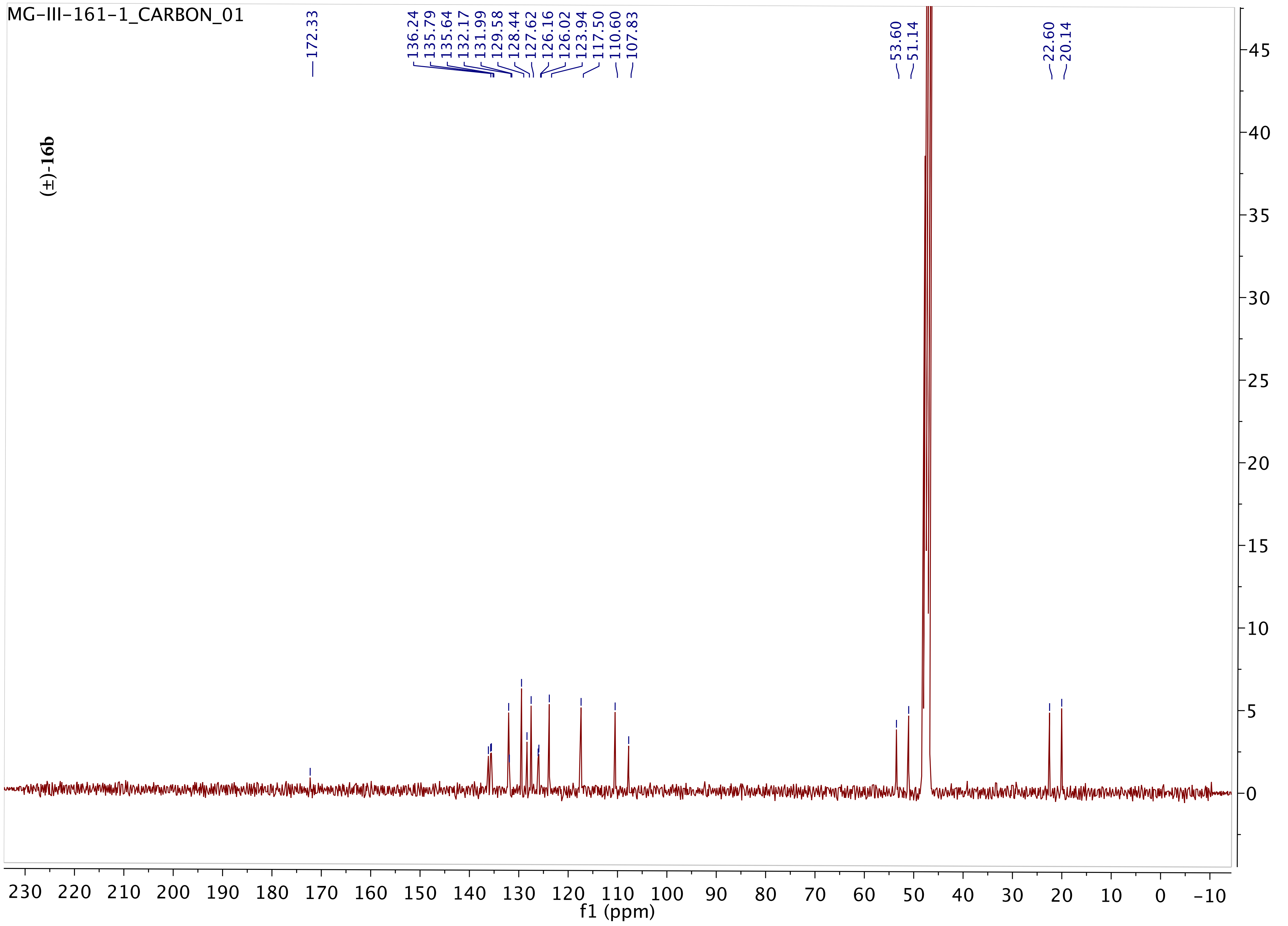
MG-III-161-1_CARBON_01
(±)-16b
172.33
136.24
135.79
135.64
132.17
131.99
129.58
128.44
127.62
126.16
126.02
123.94
117.50
110.60
107.83
53.60
51.14
22.60
20.14
f1 (ppm)

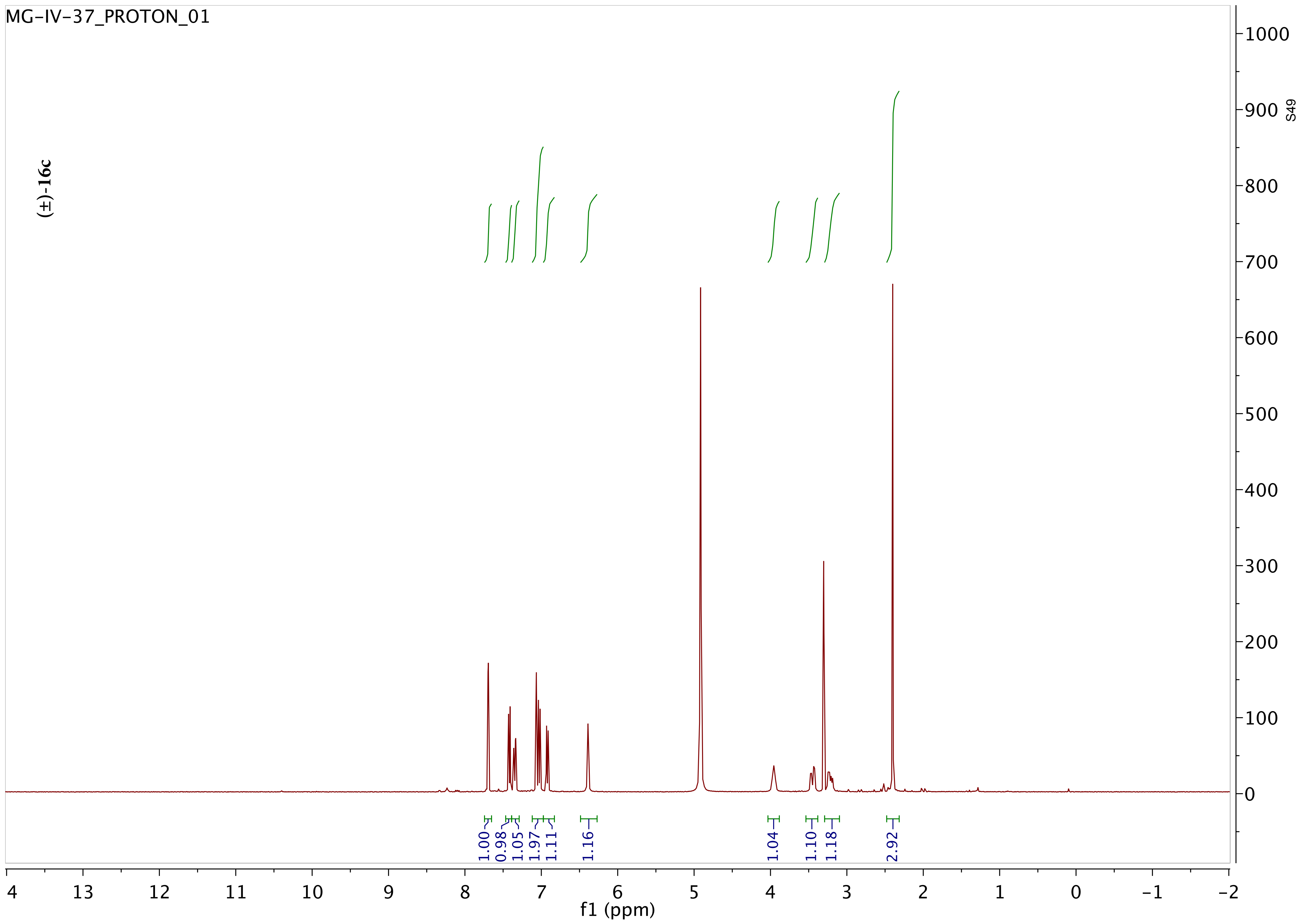
MG–IV–37_PROTON_01
(±)-16c
1.00
0.98
1.05
1.97
1.11
1.16
1.04
1.10
1.18
2.92
f1 (ppm)

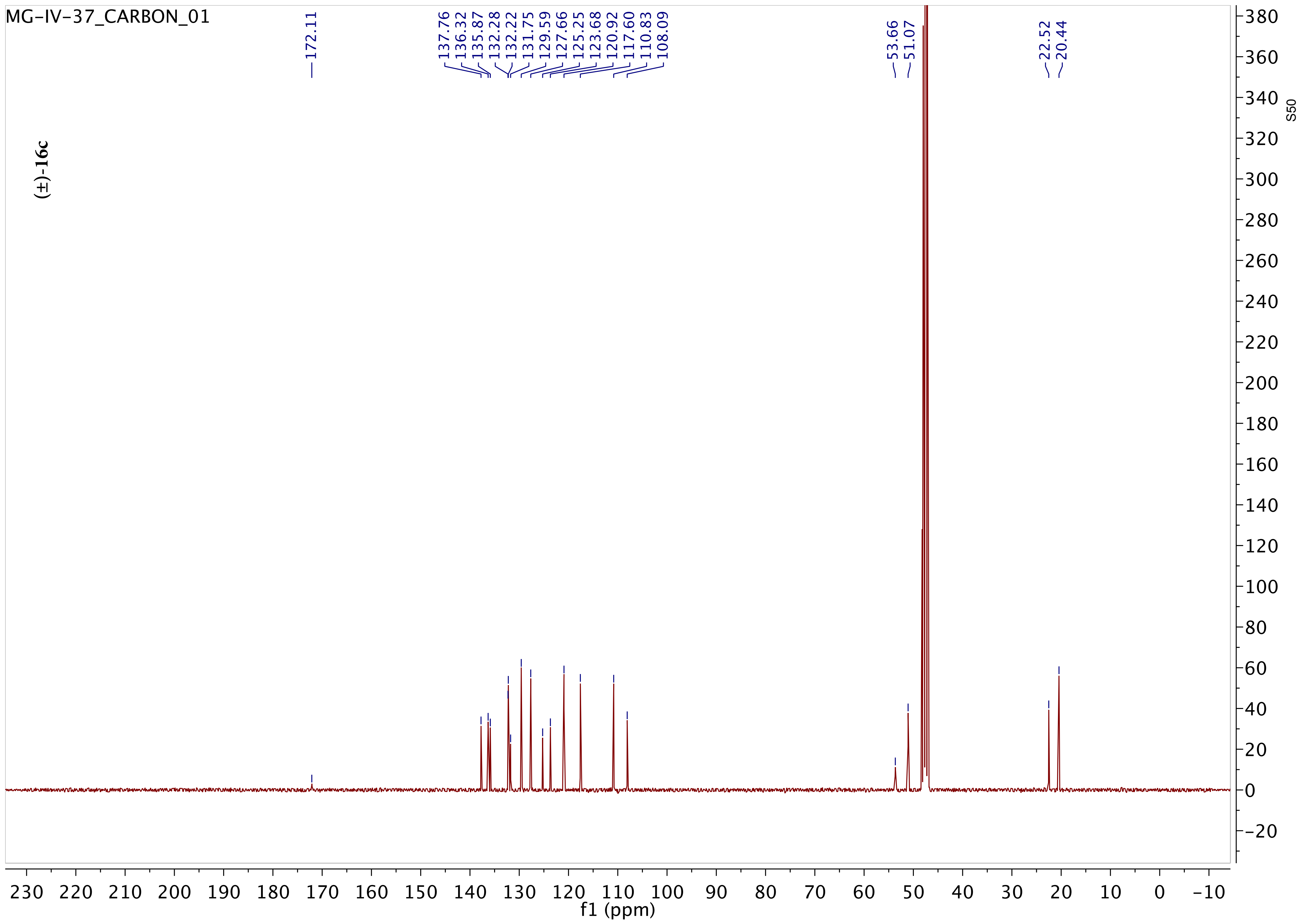

MG-IV-37_CARBON_01
(±)-16c
172.11
137.76
136.32
135.87
132.28
132.22
131.75
129.59
127.66
125.25
123.68
120.92
117.60
110.83
108.09
53.66
51.07
22.52
20.44
f1 (ppm)

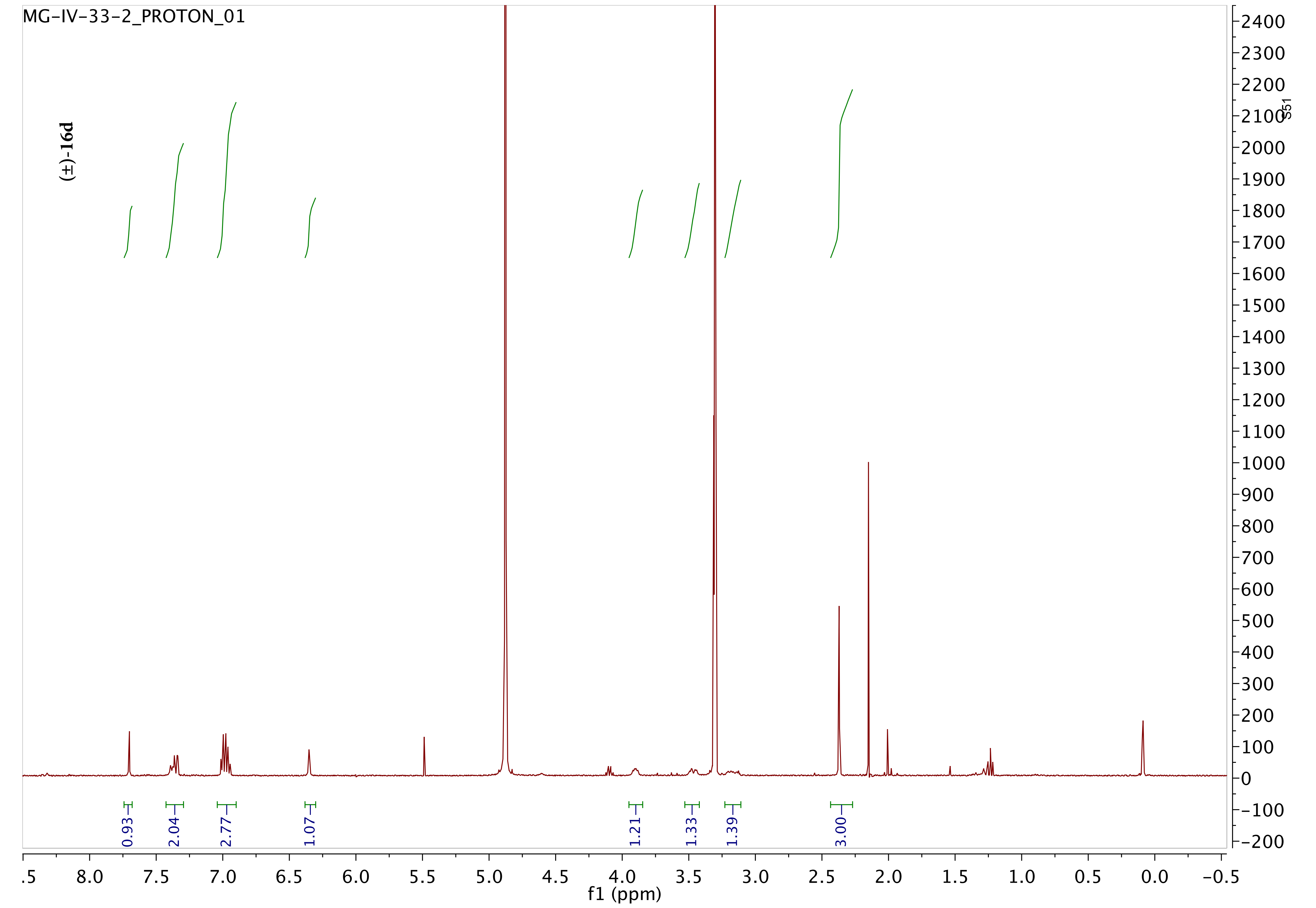
MG-IV-33-2_PROTON_01
(±)-16d
0.93
2.04
2.77
1.07
1.21
1.33
1.39
3.00
f1 (ppm)

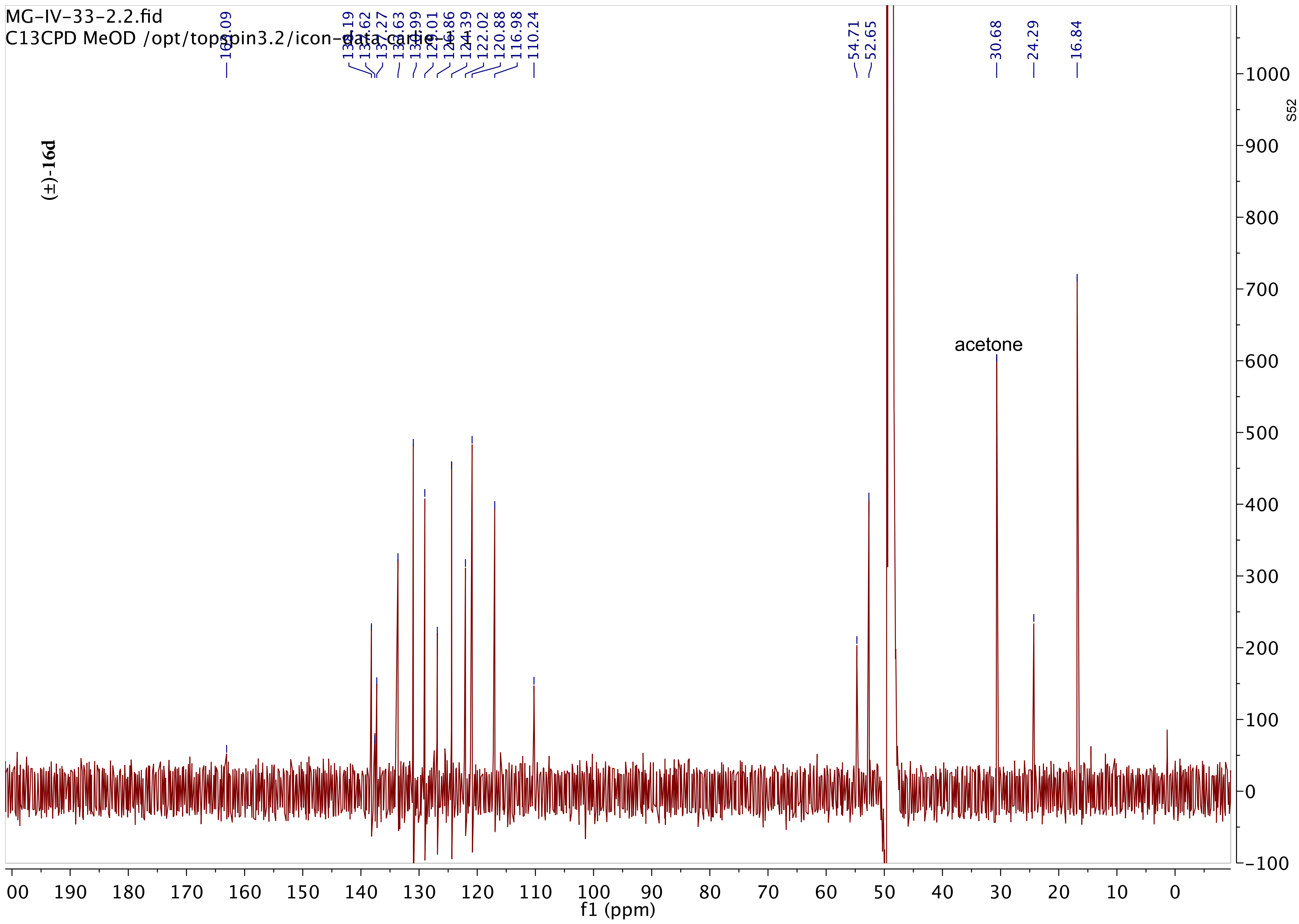
MG-IV-33-2.2.fid
C13CPD MeOD /opt/topspin3.2/icon-data carlie
(±)-16d
137.27
133.63
129.01
124.39
122.02
120.88
116.98
110.24
54.71
52.65
30.68
24.29
16.84
acetone
f1 (ppm)

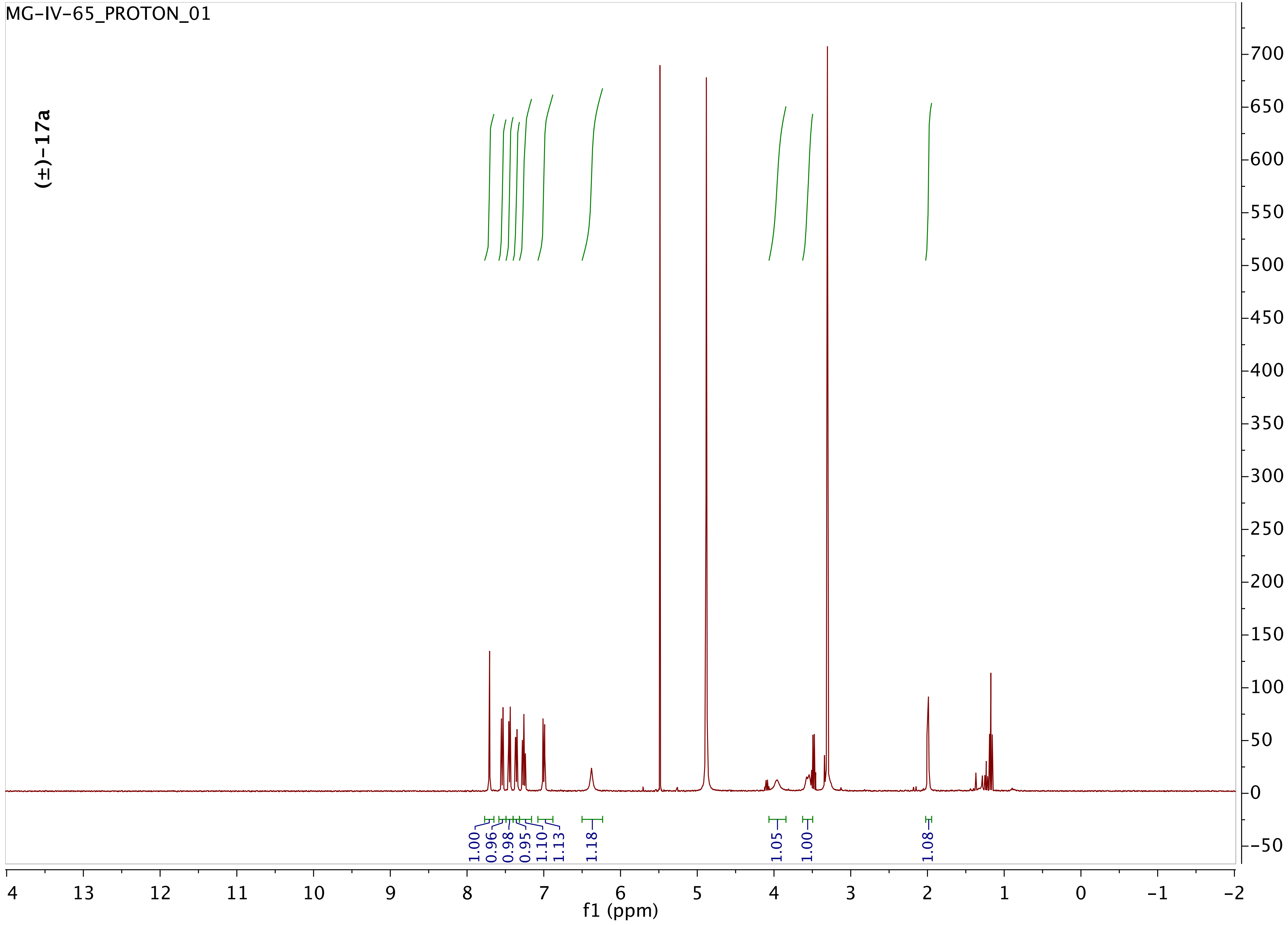
MG-IV-65_PROTON_01
(±)-17a
1.00
0.96
0.98
0.95
1.10
1.13
1.18
1.05
1.00
1.08
f1 (ppm)

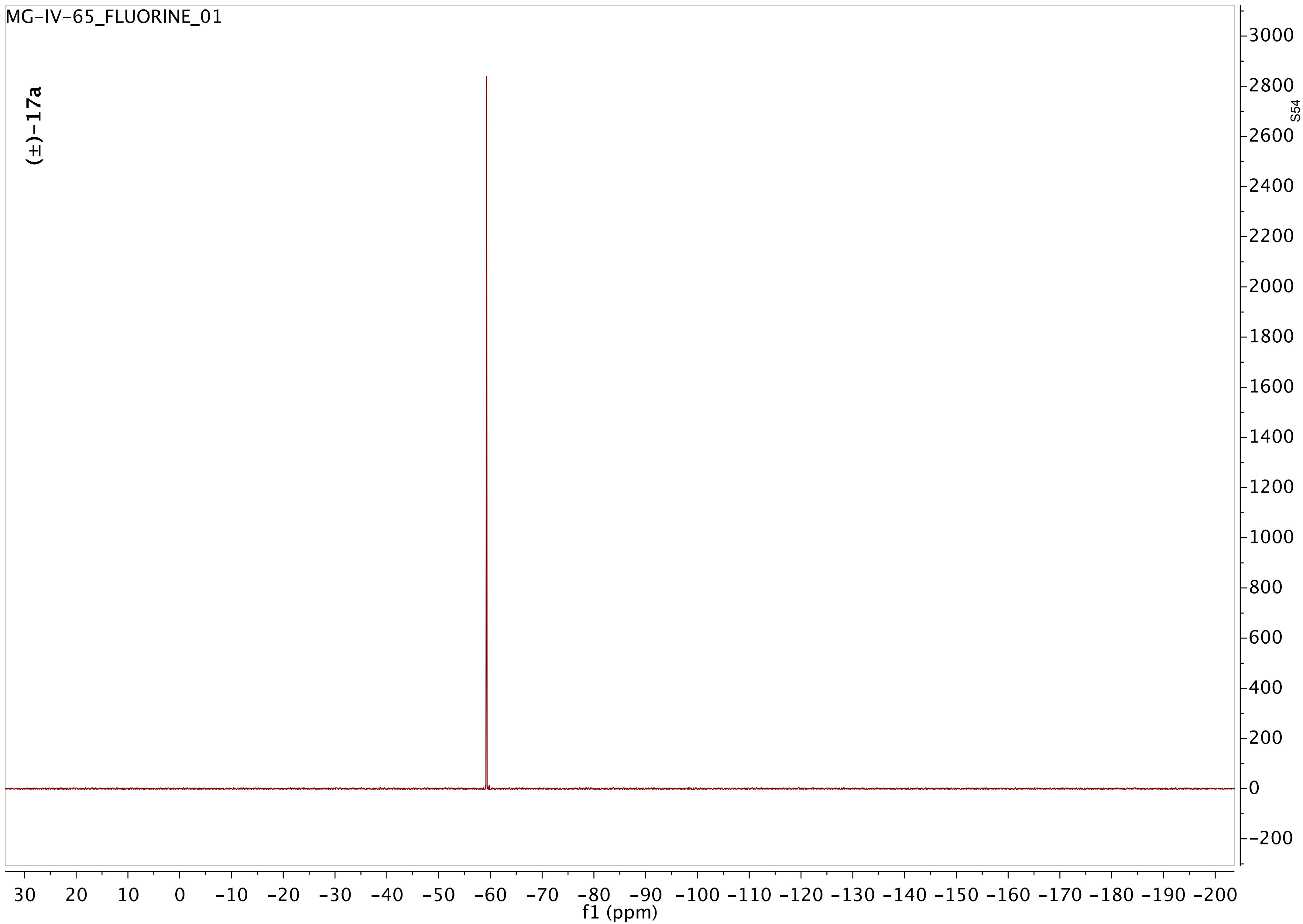
MG-IV-65_FLUORINE_01
(±)-17a
3000
2800
2600
2400
2200
2000
1800
1600
1400
1200
1000
800
600
400
200
0
-200
30 20 10 0 -10 -20 -30 -40 -50 -60 -70 -80 -90 -100 -110 -120 -130 -140 -150 -160 -170 -180 -190 -200
f1 (ppm)

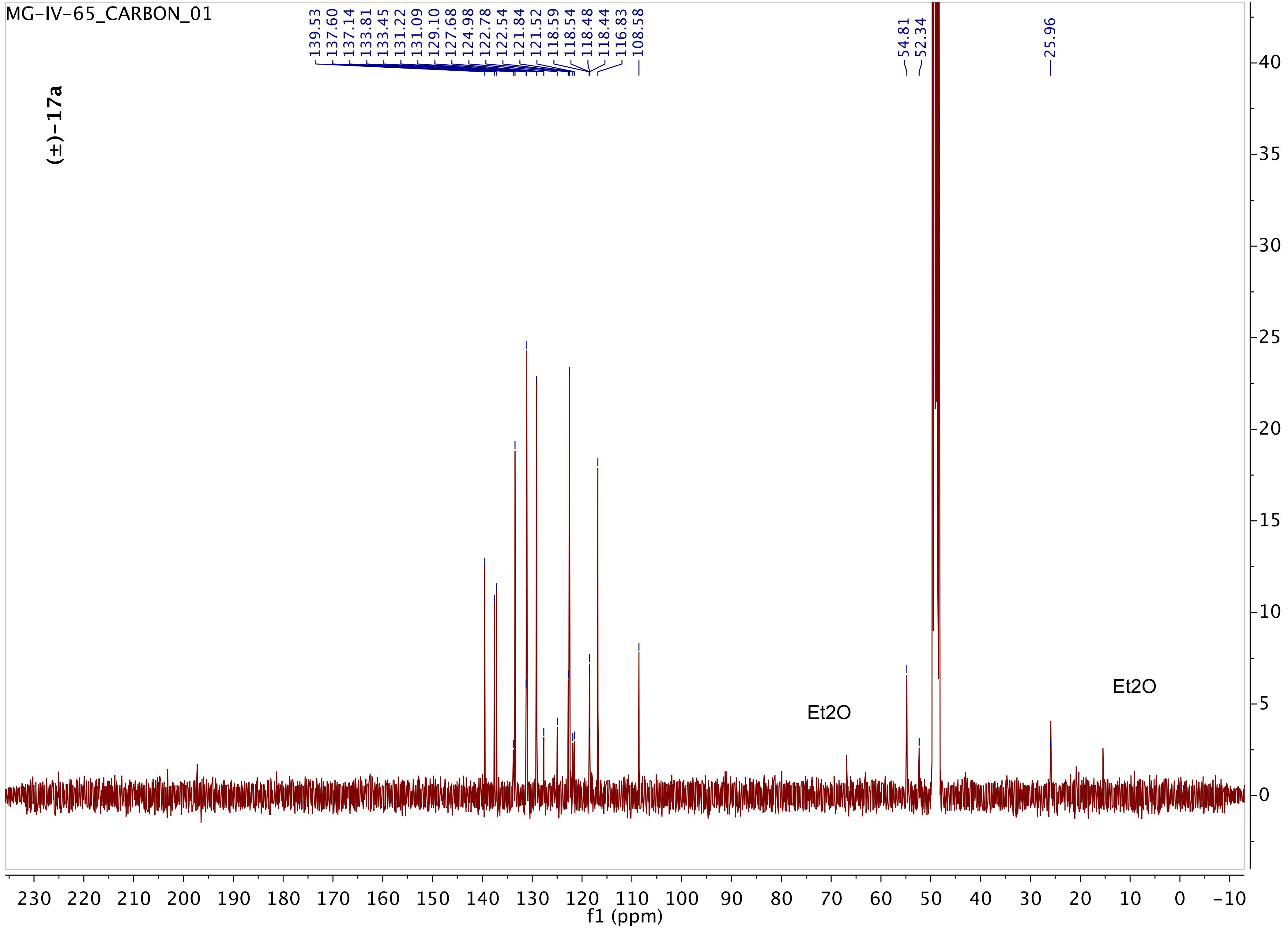
MG-IV-65_CARBON_01
(±)-17a
139.53
137.60
137.14
133.81
133.45
131.22
131.09
129.10
127.68
124.98
122.78
122.54
121.84
121.52
118.59
118.54
118.48
118.44
116.83
108.58
54.81
52.34
25.96
Et2O
Et2O
f1 (ppm)

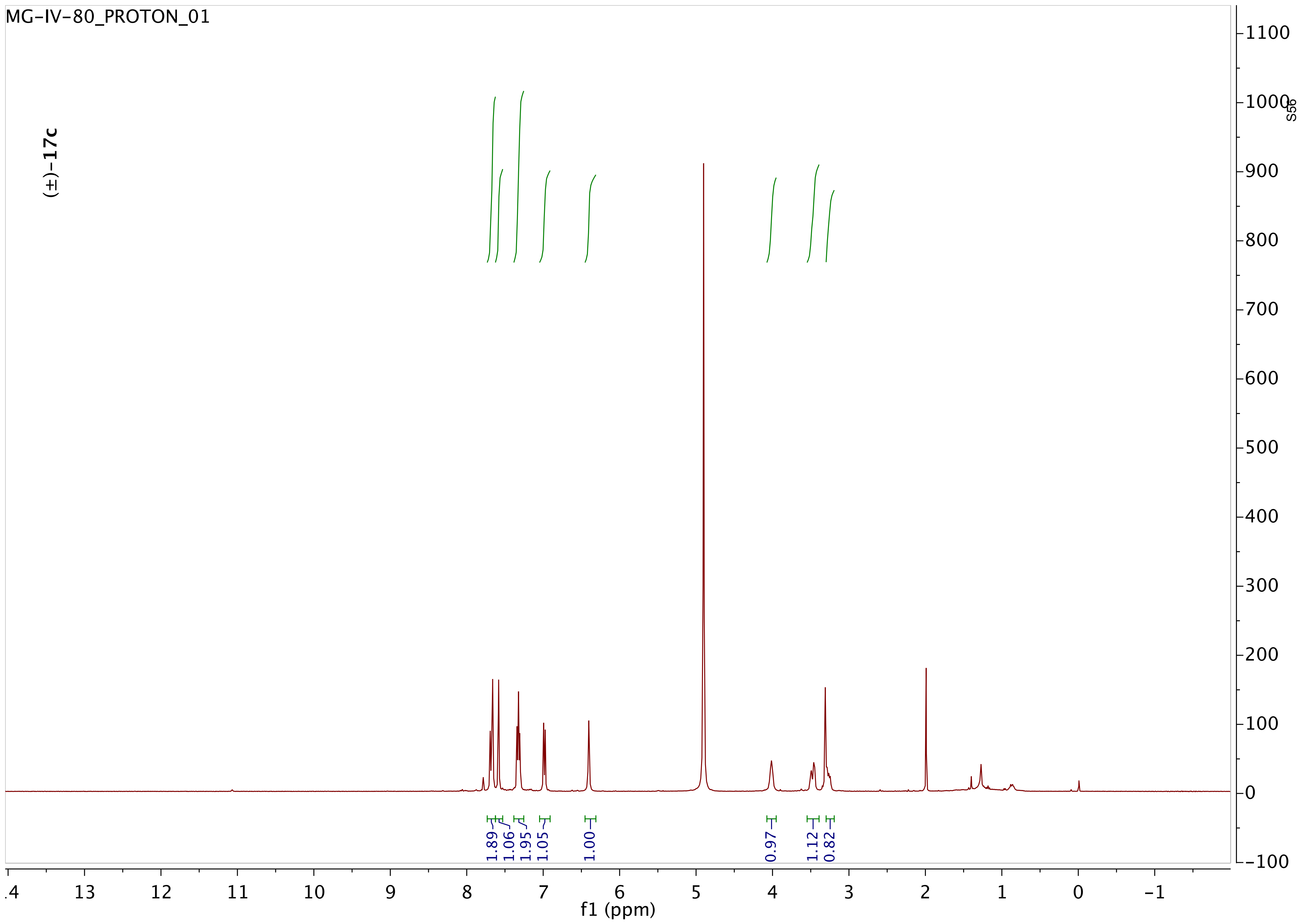
MG-IV-80_PROTON_01
(±)-17c
1.89
1.06
1.95
1.05
1.00
0.97
1.12
0.82
f1 (ppm)

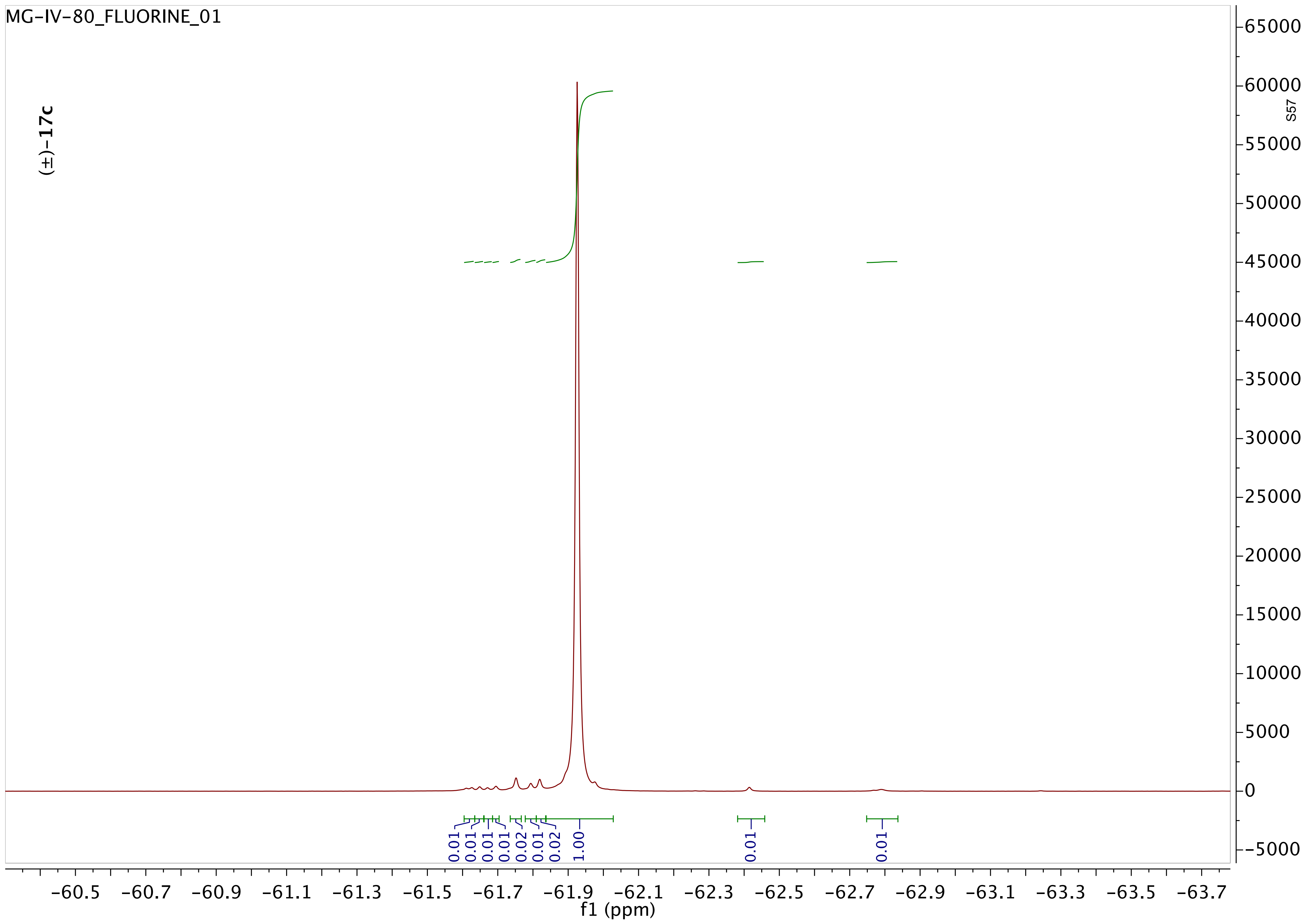
MG-IV-80_FLUORINE_01
(±)-17c
0.01
0.01
0.01
0.01
0.02
0.01
0.02
1.00
0.01
0.01
-60.5
-60.7
-60.9
-61.1
-61.3
-61.5
-61.7
-61.9
-62.1
-62.3
-62.5
-62.7
-62.9
-63.1
-63.3
-63.5
-63.7
f1 (ppm)
65000
60000
55000
50000
45000
40000
35000
30000
25000
20000
15000
10000
5000
0
-5000

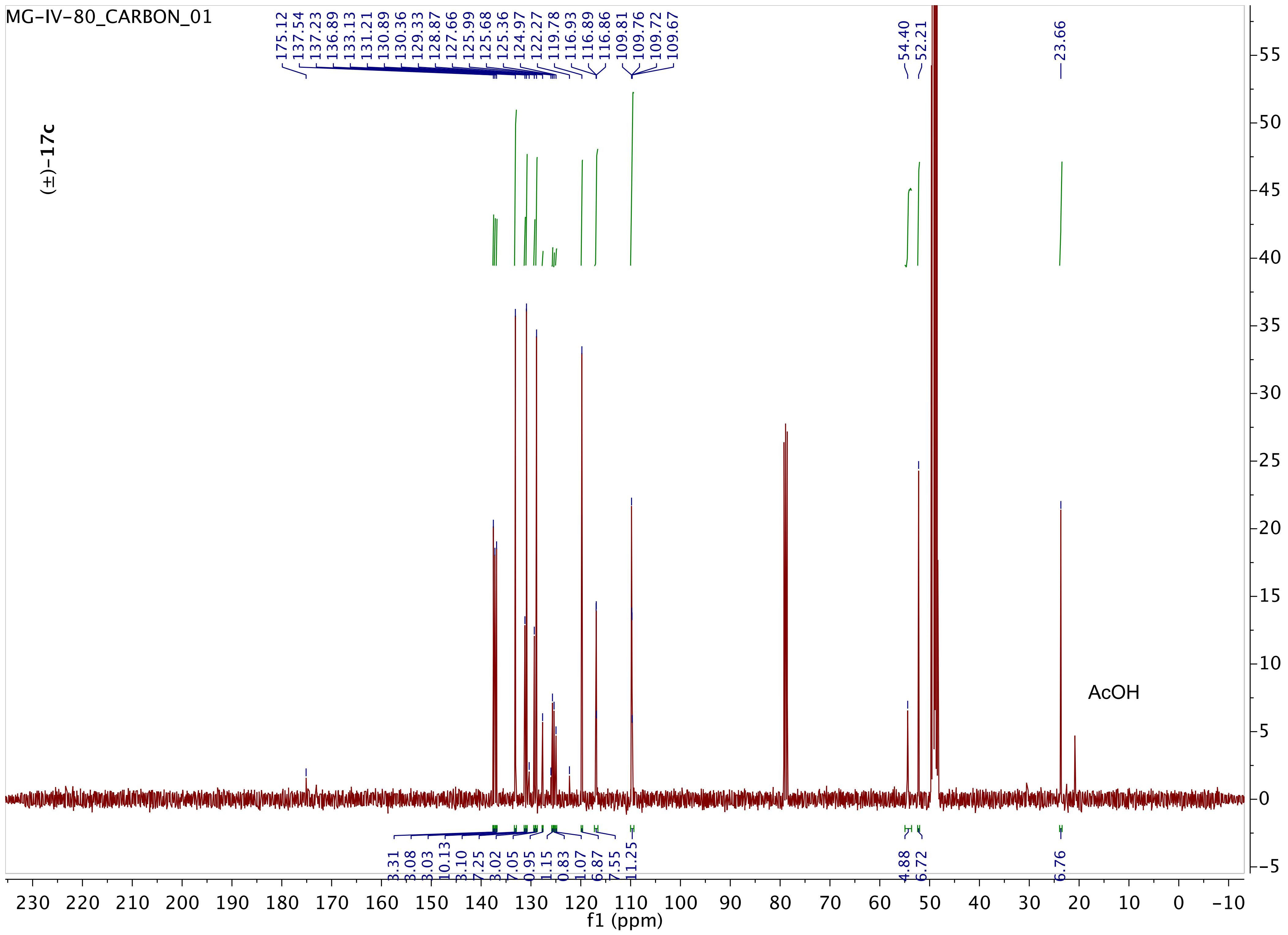

MG-IV-80_CARBON_01
(±)-17c
175.12
137.54
137.23
136.89
133.13
131.21
130.89
130.36
129.33
128.87
127.66
125.99
125.68
125.36
124.97
122.27
119.78
116.93
116.89
116.86
109.81
109.76
109.72
109.67
54.40
52.21
23.66
AcOH
3.31
3.08
3.03
10.13
3.10
7.25
3.02
7.05
0.95
1.15
0.83
1.07
6.87
7.55
11.25
4.88
6.72
6.76
f1 (ppm)

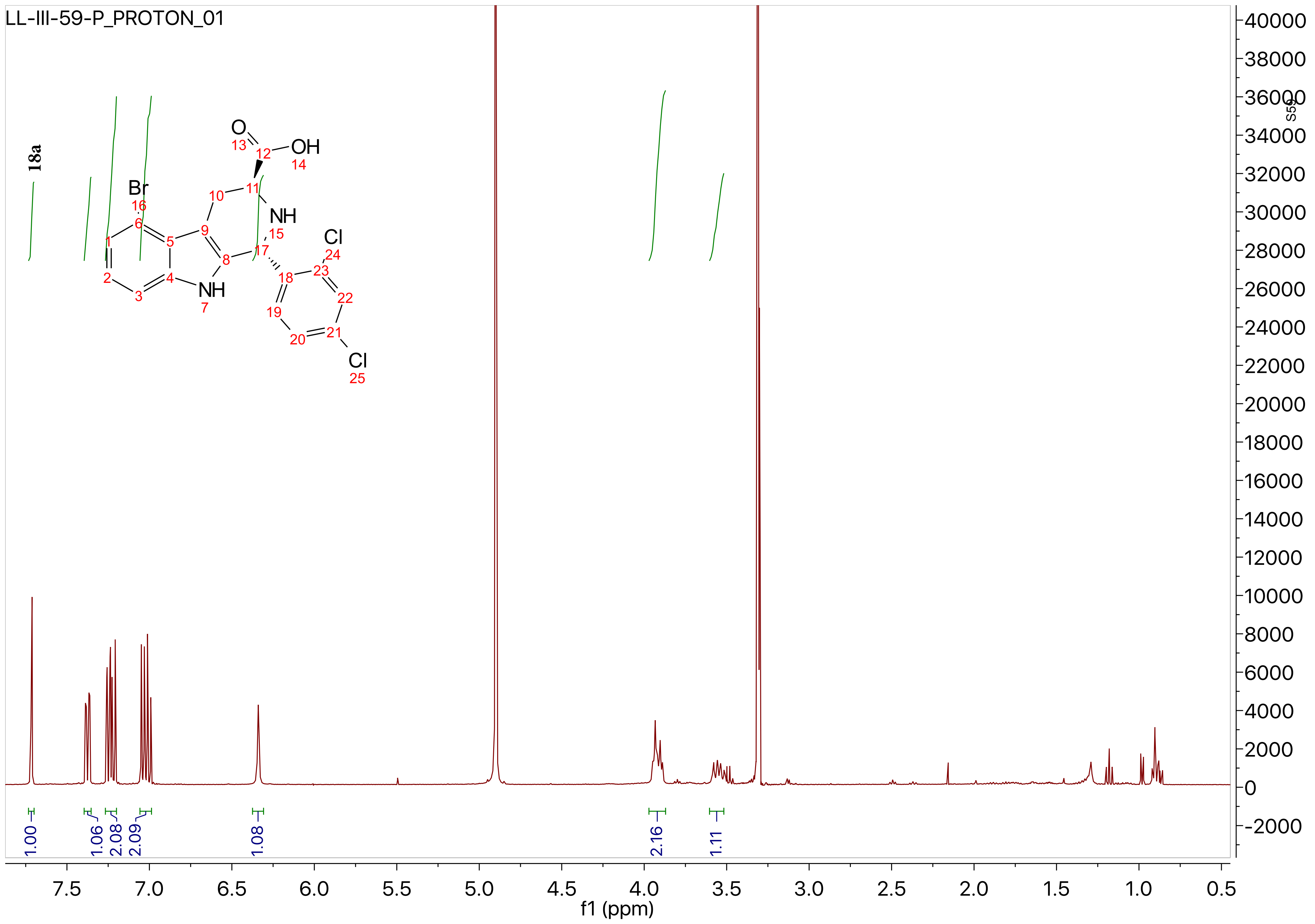
LL-III-59-P_PROTON_01
18a
Br
NH
NH
O
OH
Cl
Cl
1.00
1.06
2.08
2.09
1.08
2.16
1.11
f1 (ppm)

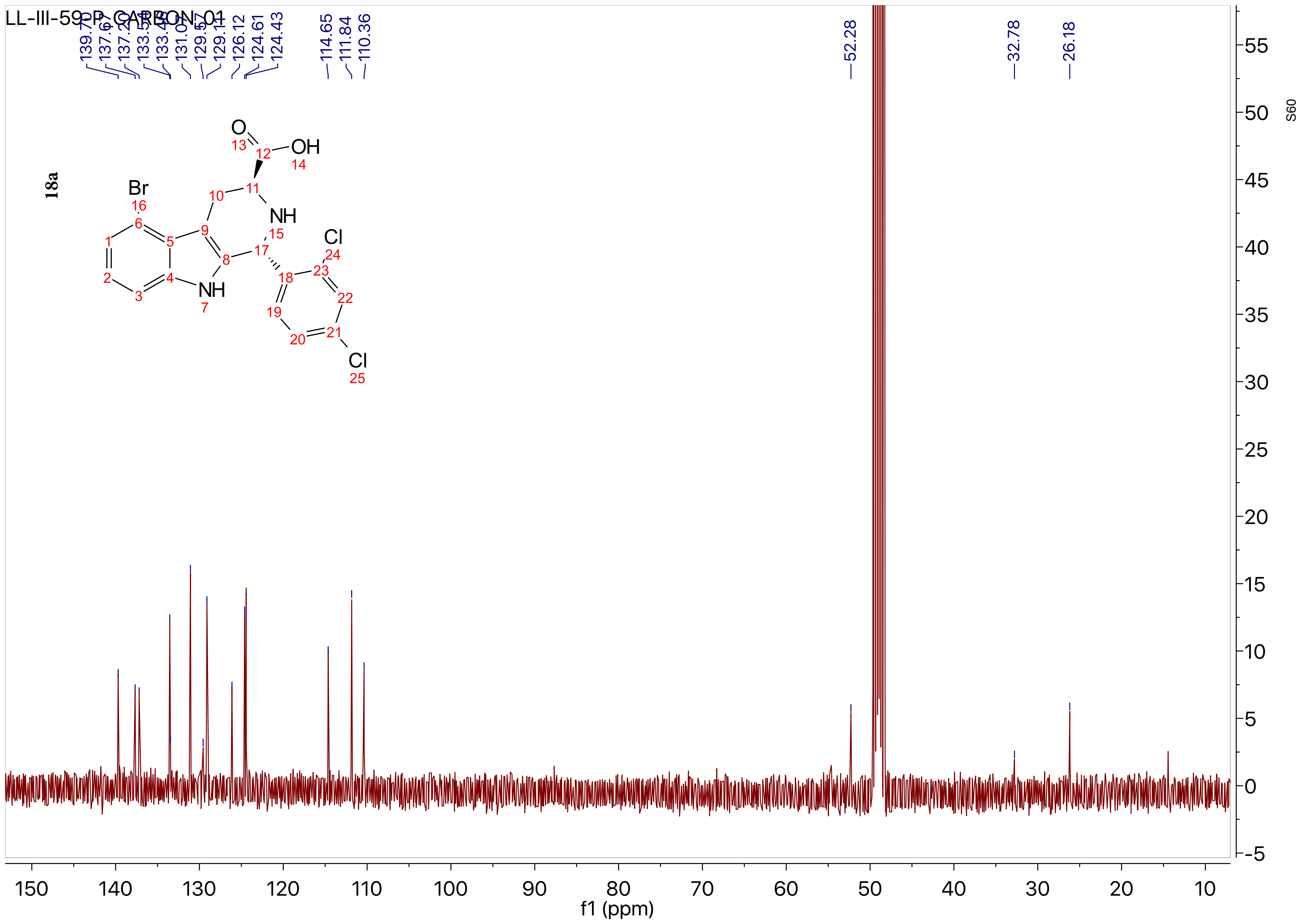
LL-III-59-P CARBON_01
18a
139.70
129.57
129.11
126.12
124.61
124.43
114.65
111.84
110.36
52.28
32.78
26.18
Br
O
OH
NH
Cl
Cl
f1 (ppm)

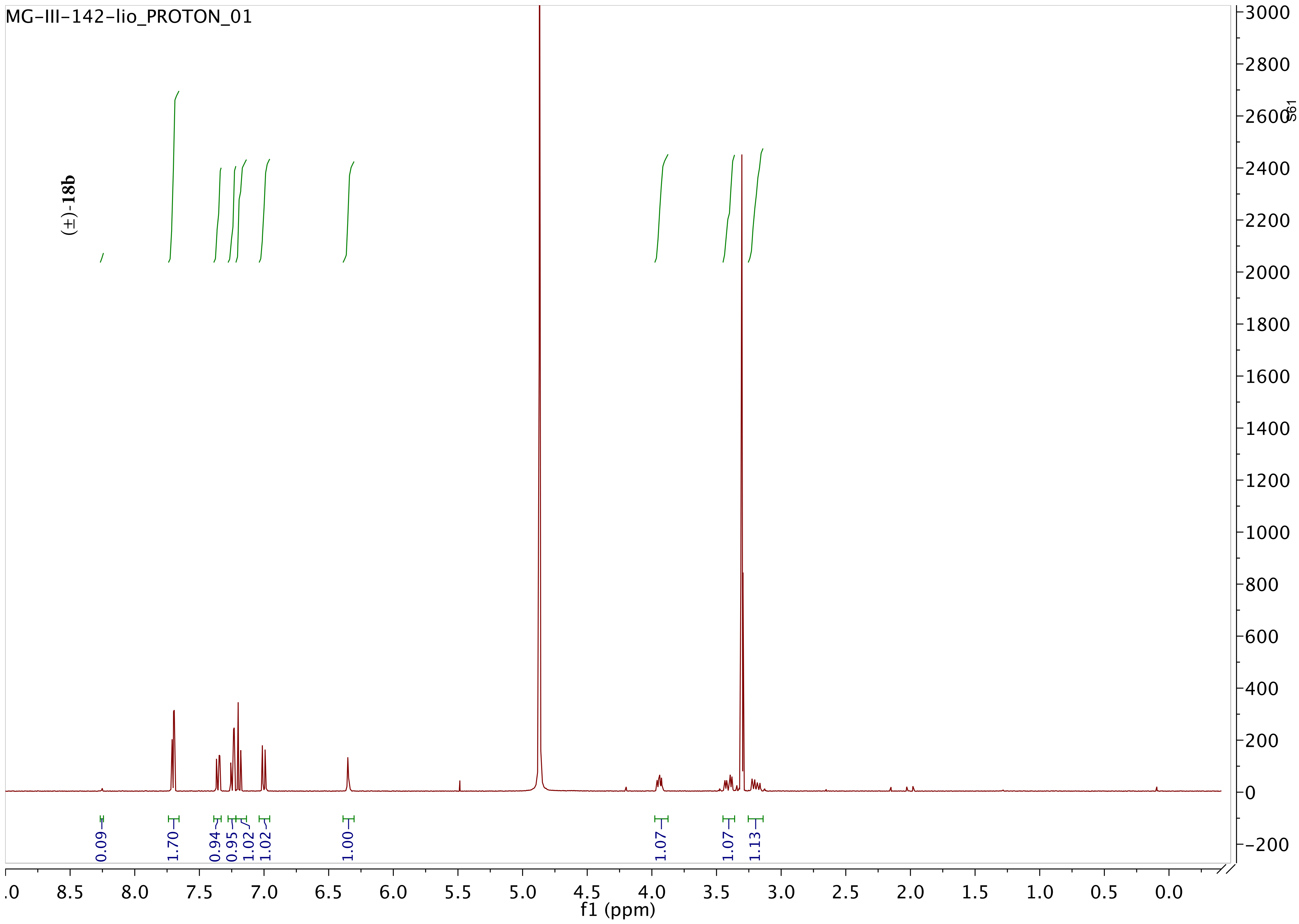
MG–III–142–lio_PROTON_01
(±)-18b
0.09
1.70
0.94
0.95
1.02
1.02
1.00
1.07
1.07
1.13
f1 (ppm)

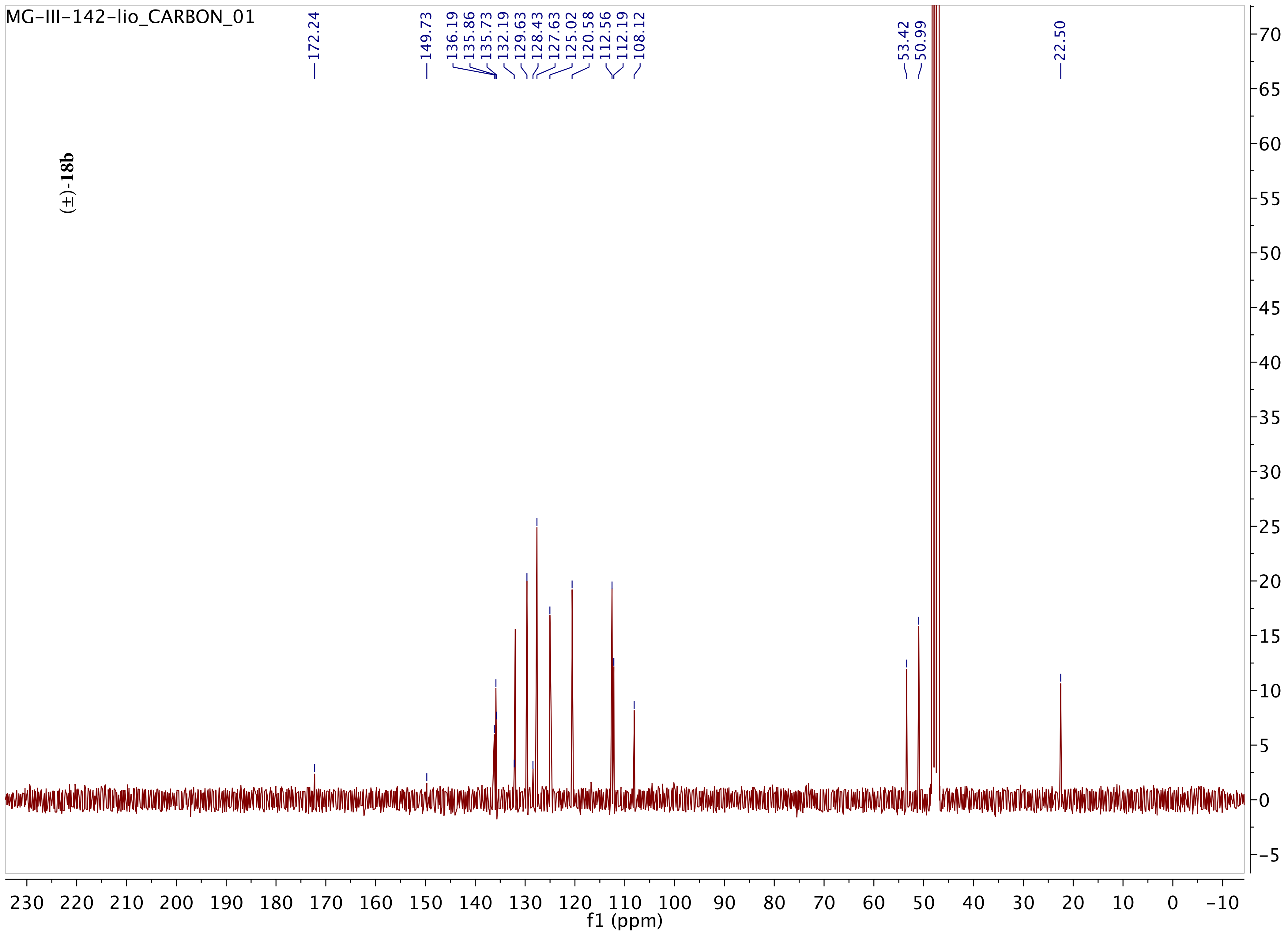

MG-III-142-lio_CARBON_01
(±)-18b
172.24
149.73
136.19
135.86
135.73
132.19
129.63
128.43
127.63
125.02
120.58
112.56
112.19
108.12
53.42
50.99
22.50
230 220 210 200 190 180 170 160 150 140 130 120 110 100 90 80 70 60 50 40 30 20 10 0 -10
f1 (ppm)
-5 0 5 10 15 20 25 30 35 40 45 50 55 60 65 70

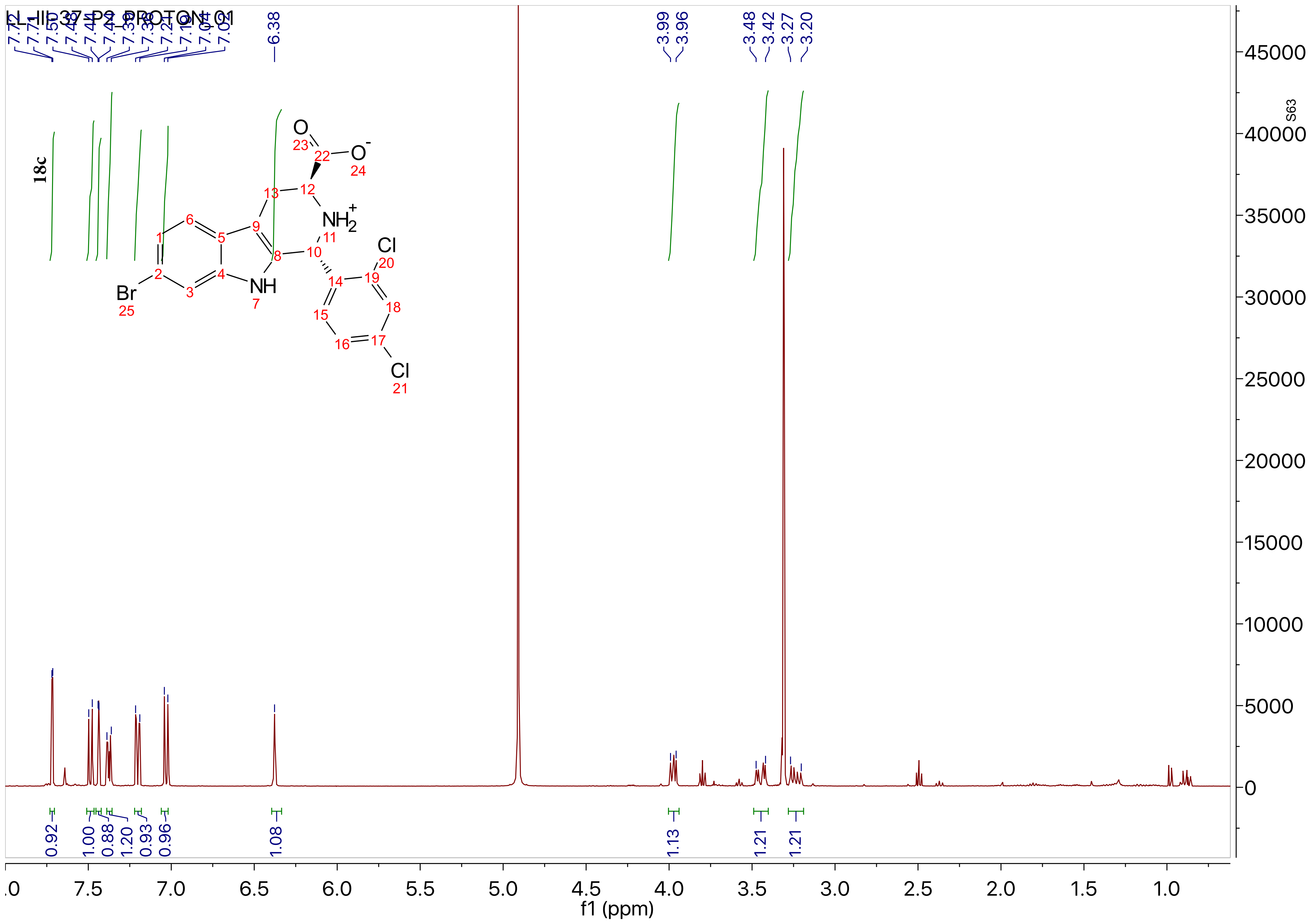
LL-III-37-P2_PROTON-01
18c
7.72
7.71
7.50
7.48
7.44
7.44
7.39
7.38
7.21
7.19
7.04
7.02
6.38
3.99
3.96
3.48
3.42
3.27
3.20
0.92
1.00
0.88
1.20
0.93
0.96
1.08
1.13
1.21
1.21
f1 (ppm)
S63

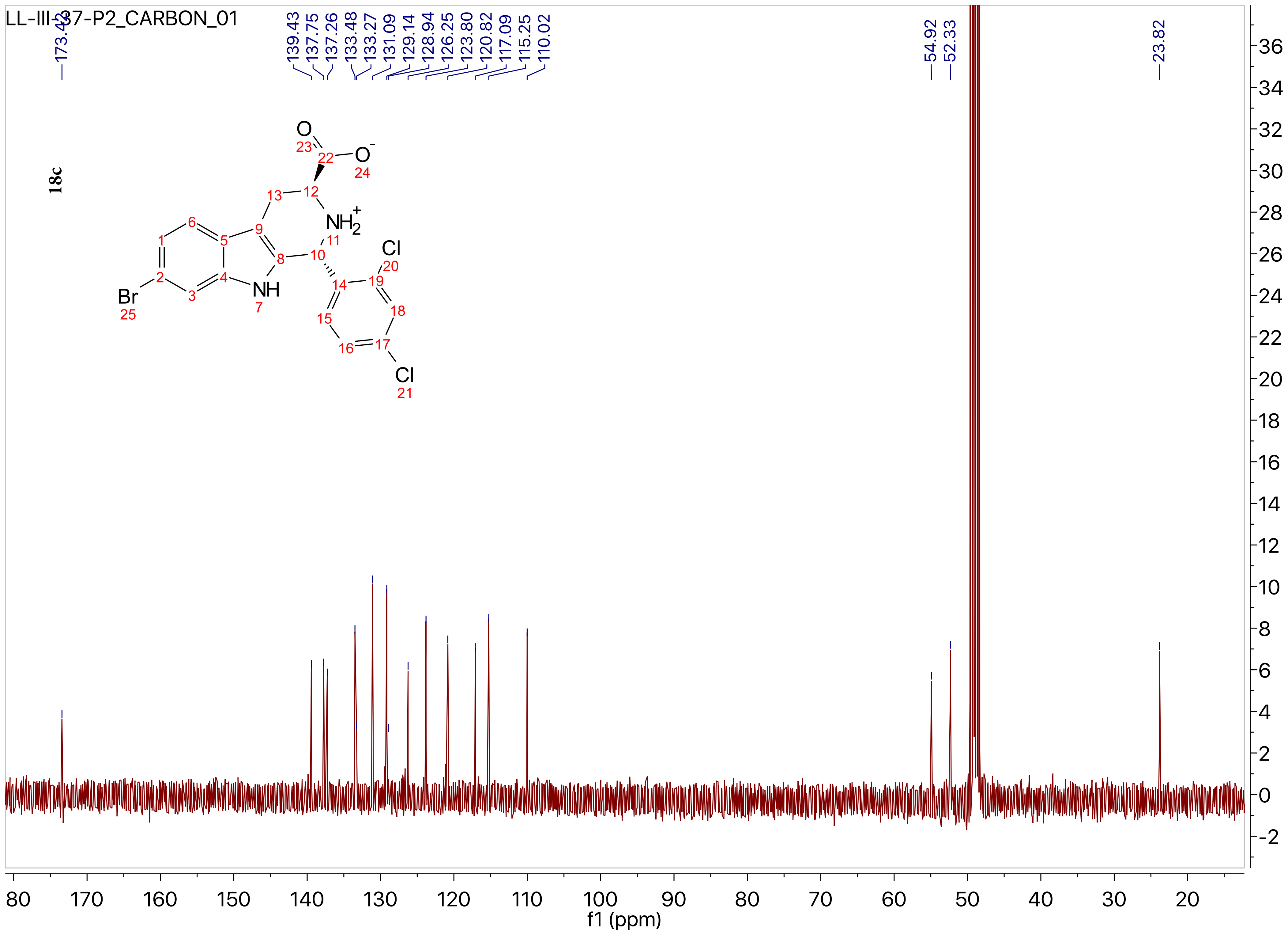
LL-III-37-P2_CARBON_01
18c
173.42
139.43
137.75
137.26
133.48
133.27
131.09
129.14
128.94
126.25
123.80
120.82
117.09
115.25
110.02
54.92
52.33
23.82
f1 (ppm)

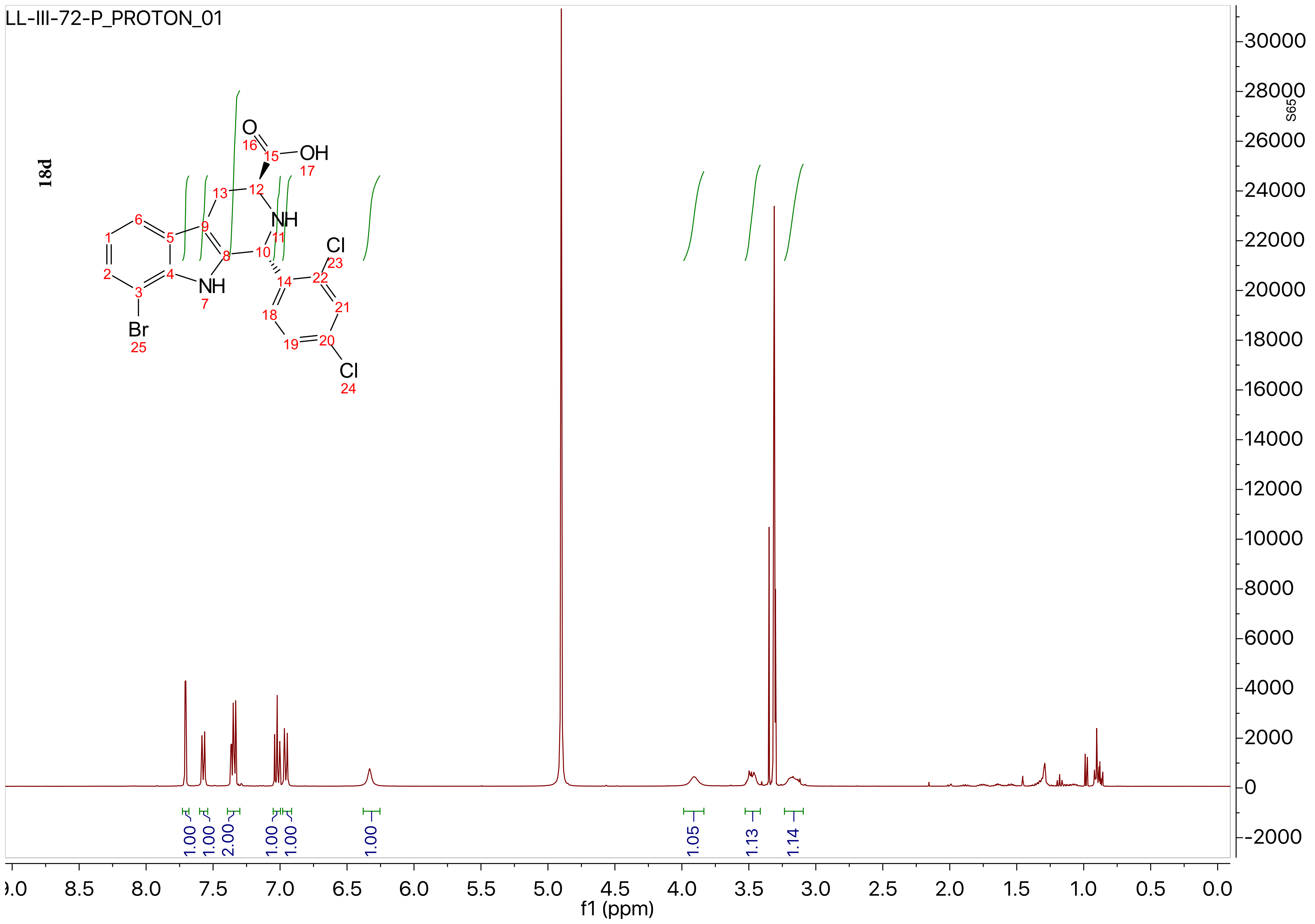
LL-III-72-P_PROTON_01
18d
1.00
1.00
2.00
1.00
1.00
1.00
1.05
1.13
1.14
f1 (ppm)

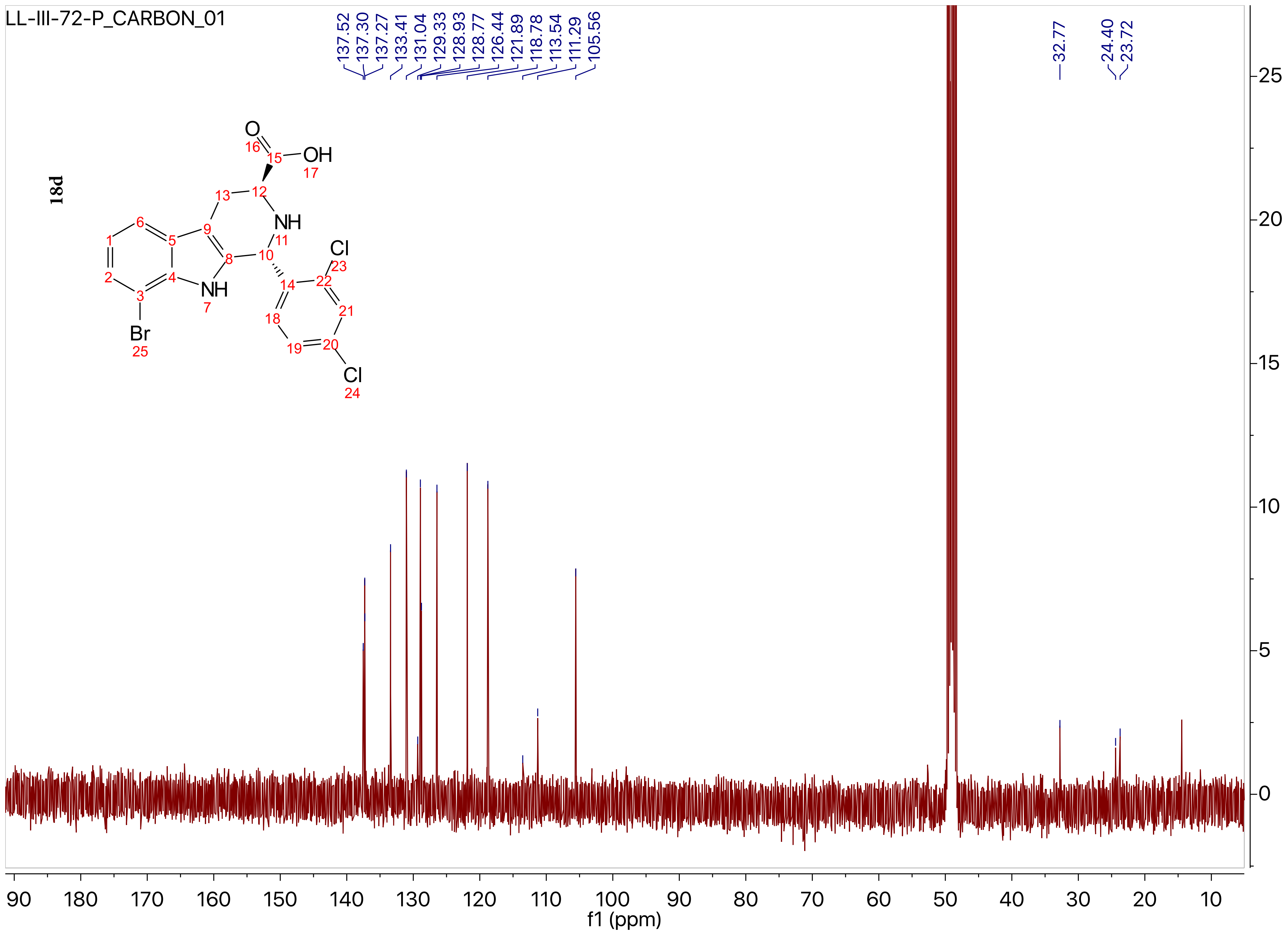
LL-III-72-P_CARBON_01
18d
137.52
137.30
137.27
133.41
131.04
129.33
128.93
128.77
126.44
121.89
118.78
113.54
111.29
105.56
32.77
24.40
23.72
Br
NH
NH
O
OH
Cl
Cl
f1 (ppm)

MG-IV-36_PROTON_01

(±)-**19a**

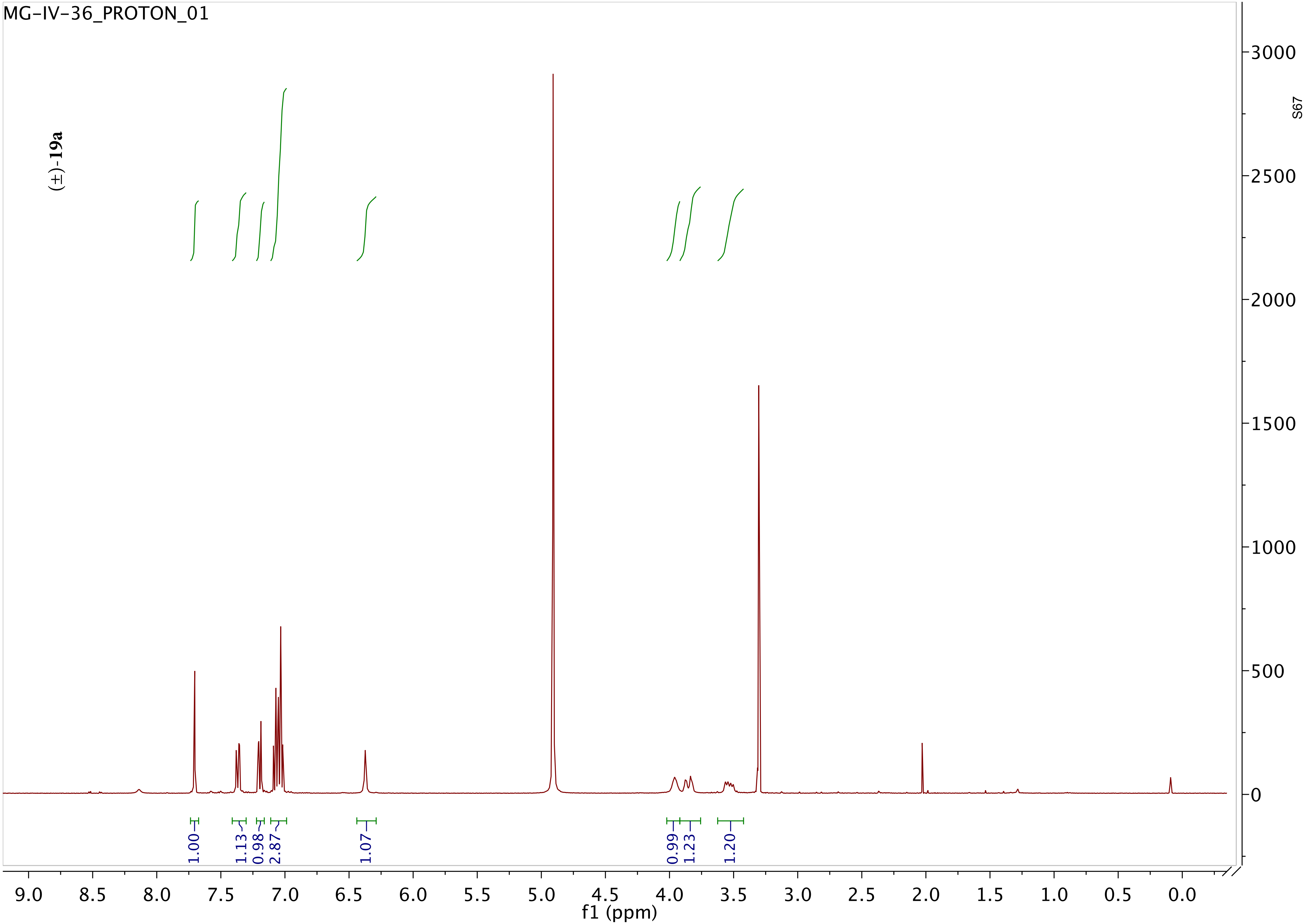

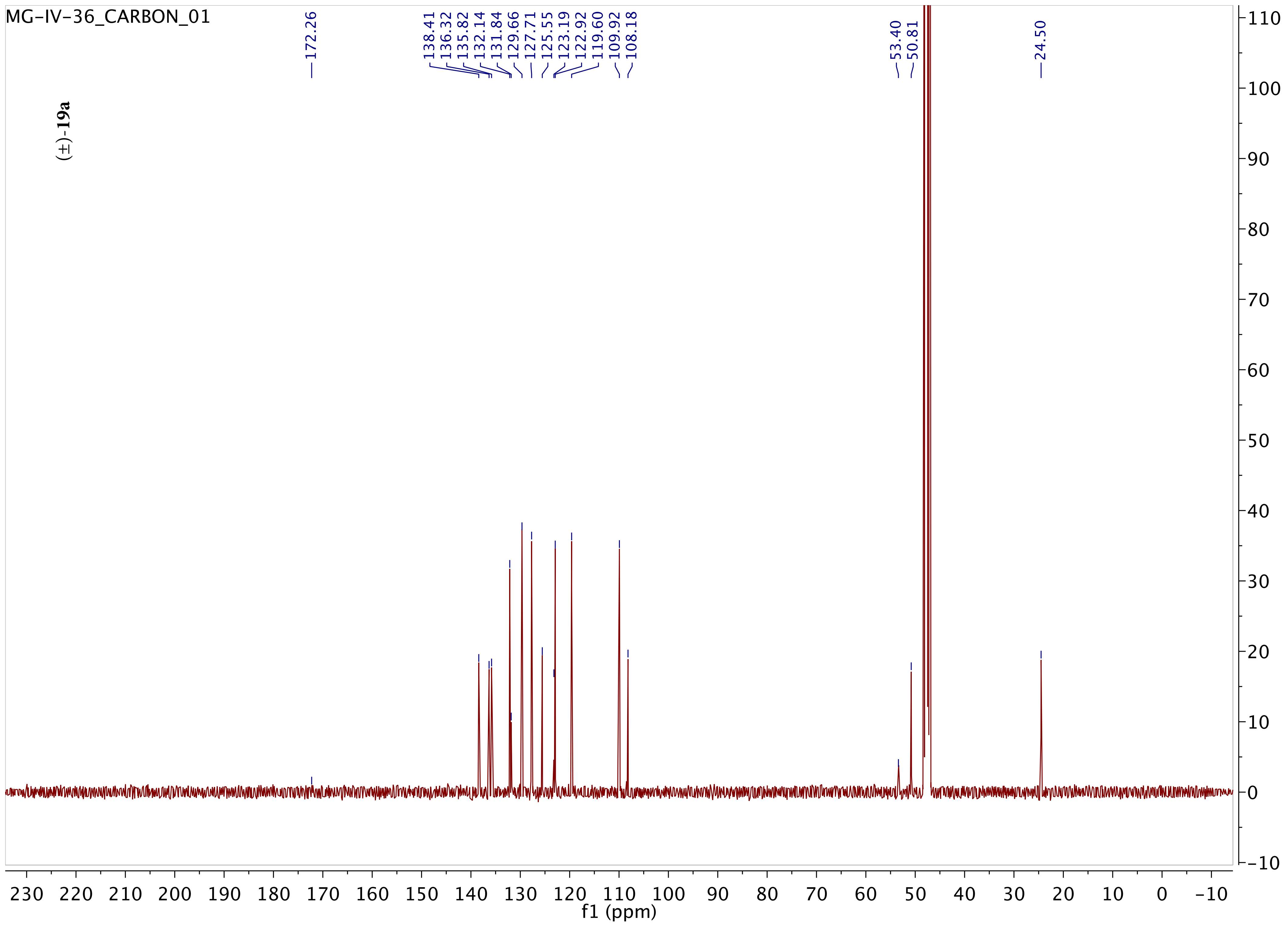
MG-IV-36_CARBON_01
(±)-19a
172.26
138.41
136.32
135.82
132.14
131.84
129.66
127.71
125.55
123.19
122.92
119.60
109.92
108.18
53.40
50.81
24.50
230 220 210 200 190 180 170 160 150 140 130 120 110 100 90 80 70 60 50 40 30 20 10 0 −10
f1 (ppm)
110 100 90 80 70 60 50 40 30 20 10 0 −10

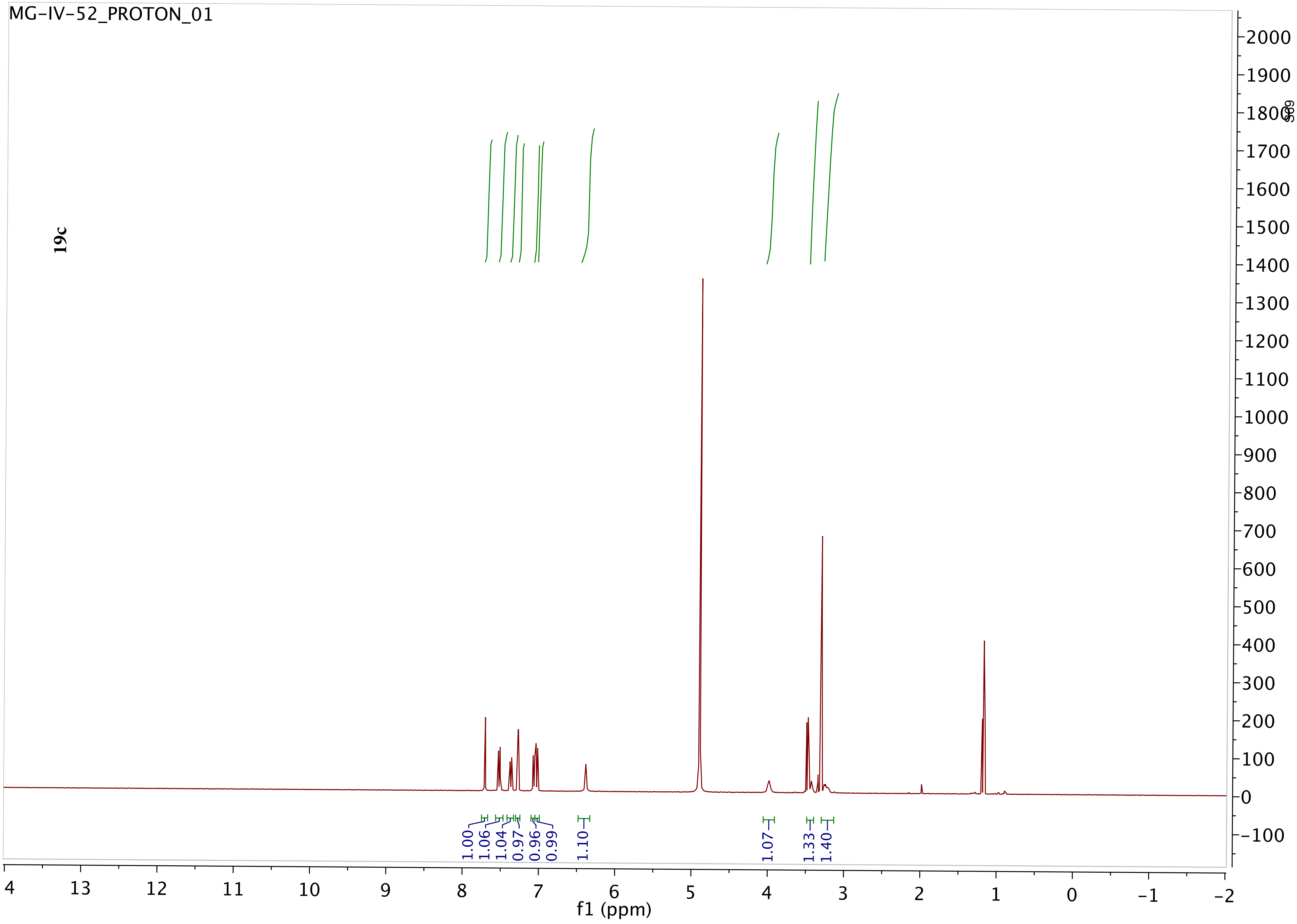
MG-IV-52_PROTON_01
19c
1.00
1.06
1.04
0.97
0.96
0.99
1.10
1.07
1.33
1.40
f1 (ppm)

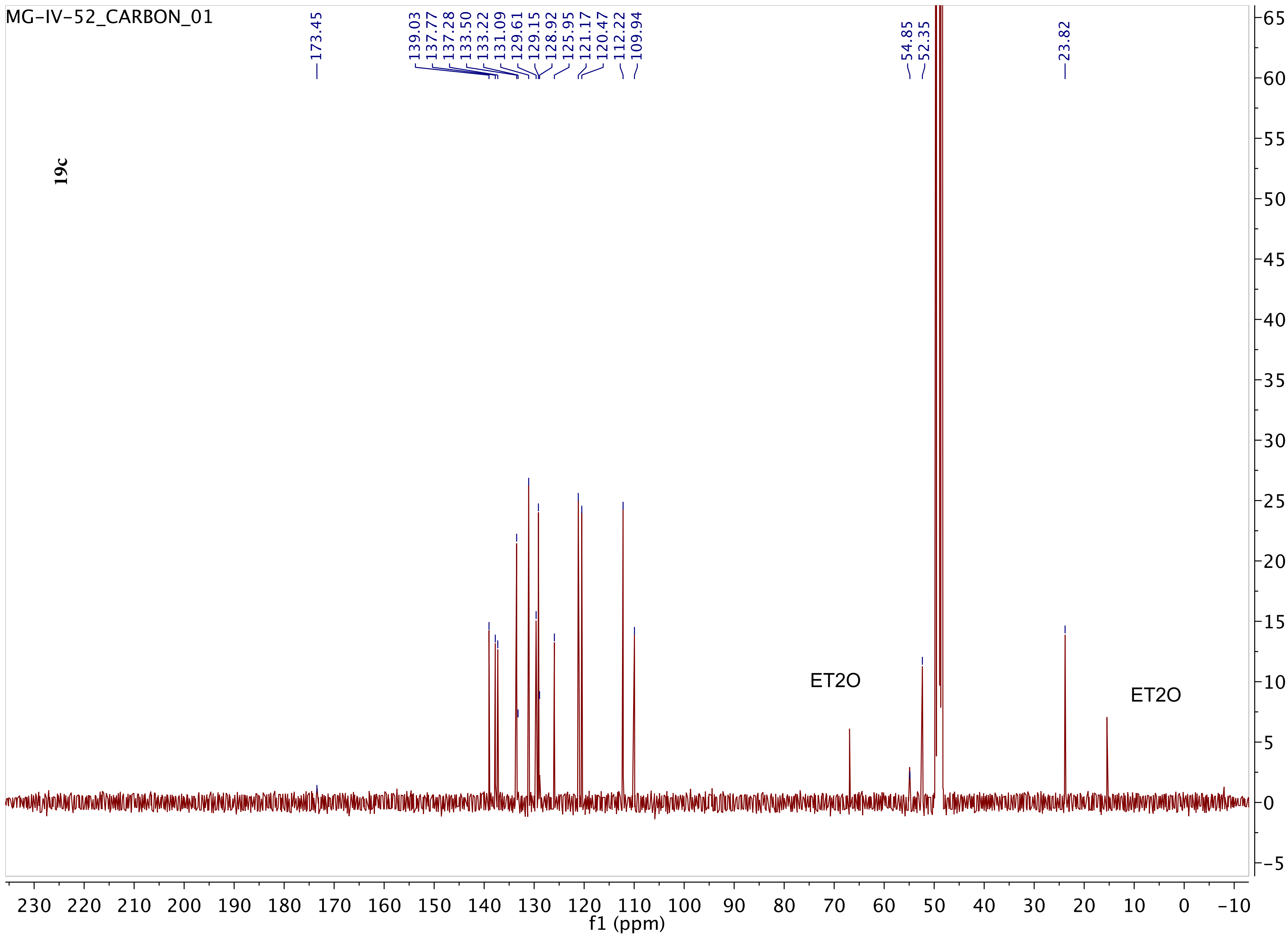
MG-IV-52_CARBON_01
19c
173.45
139.03
137.77
137.28
133.50
133.22
131.09
129.61
129.15
128.92
125.95
121.17
120.47
112.22
109.94
54.85
52.35
23.82
ET2O
ET2O
f1 (ppm)

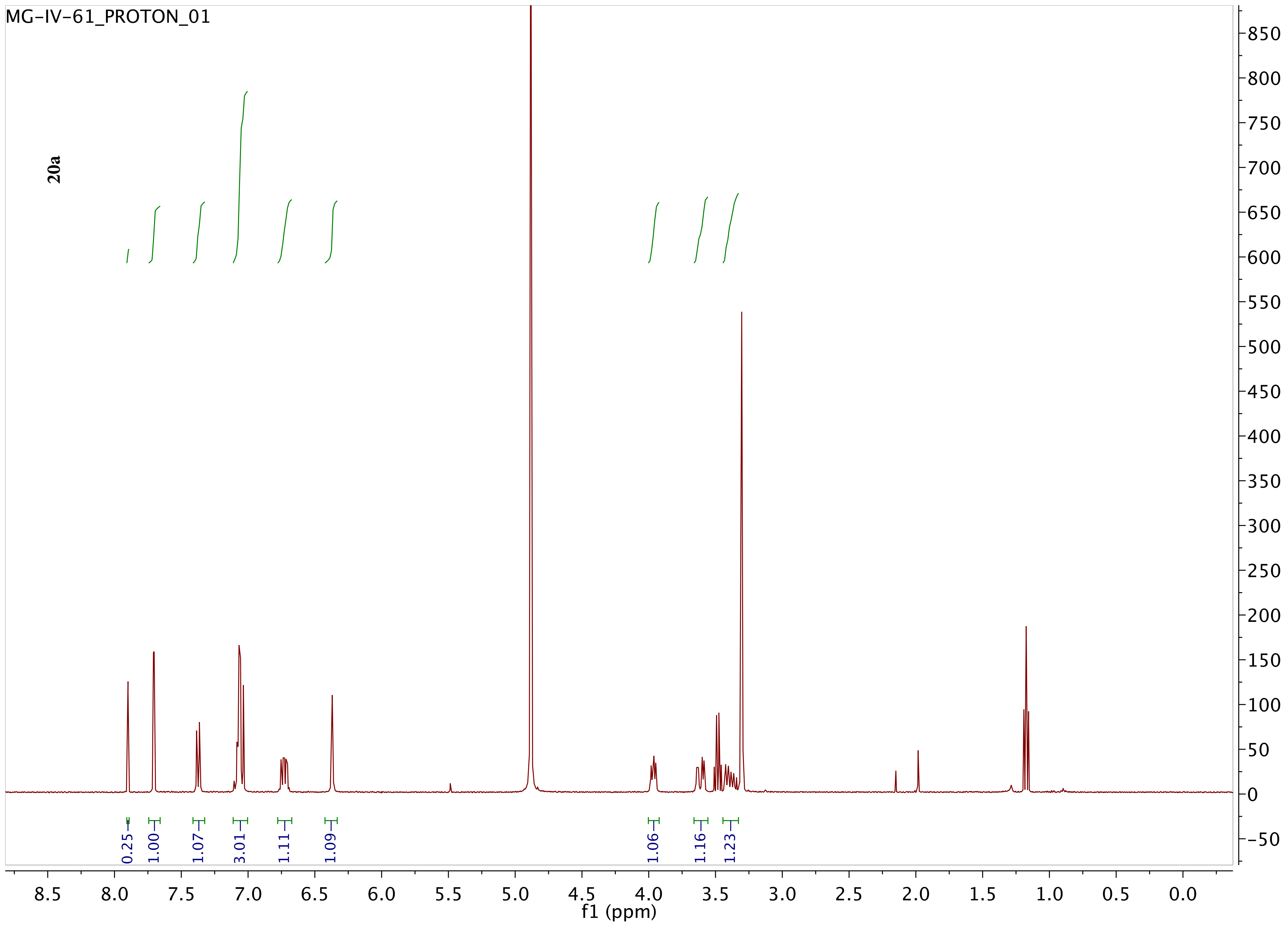
MG-IV-61_PROTON_01
20a
0.25
1.00
1.07
3.01
1.11
1.09
1.06
1.16
1.23
8.5
8.0
7.5
7.0
6.5
6.0
5.5
5.0
4.5
4.0
3.5
3.0
2.5
2.0
1.5
1.0
0.5
0.0
f1 (ppm)
850
800
750
700
650
600
550
500
450
400
350
300
250
200
150
100
50
0
–50

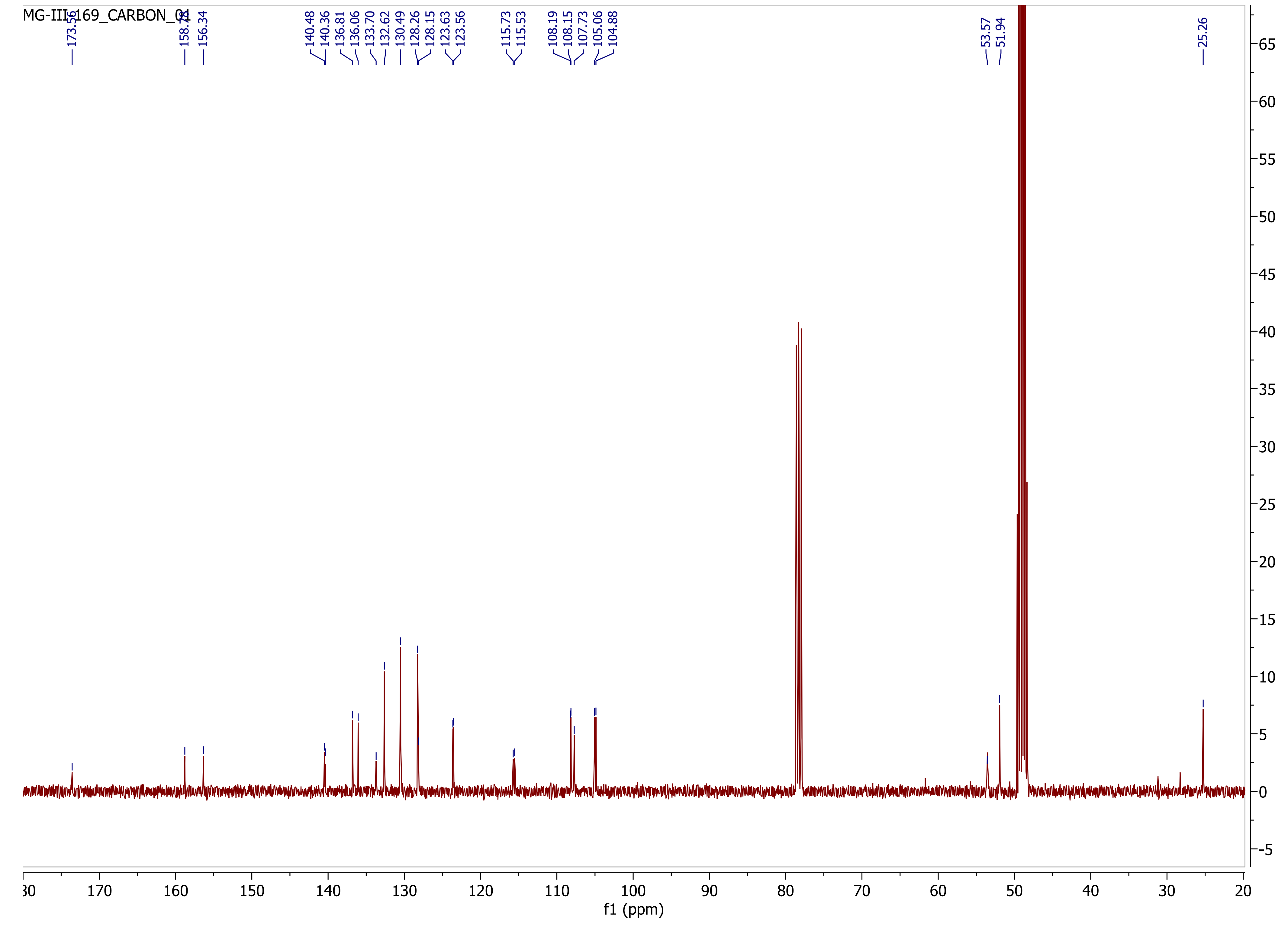
MG-III-169_CARBON_01
173.56
158.78
156.34
140.48
140.36
136.81
136.06
133.70
132.62
130.49
128.26
128.15
123.63
123.56
115.73
115.53
108.19
108.15
107.73
105.06
104.88
53.57
51.94
25.26
f1 (ppm)

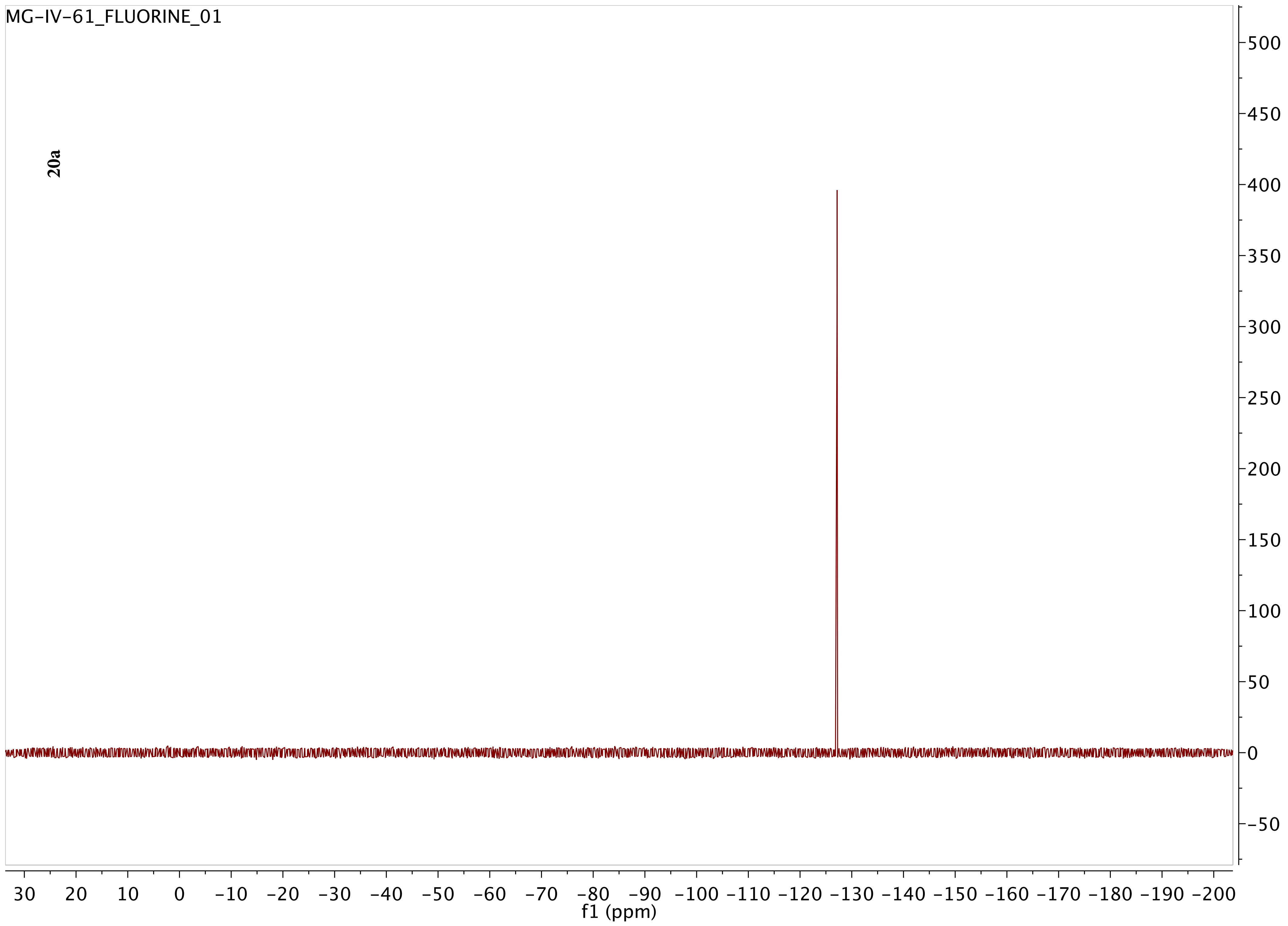

MG-IV-61_FLUORINE_01
20a
500
450
400
350
300
250
200
150
100
50
0
-50
30 20 10 0 -10 -20 -30 -40 -50 -60 -70 -80 -90 -100 -110 -120 -130 -140 -150 -160 -170 -180 -190 -200
f1 (ppm)

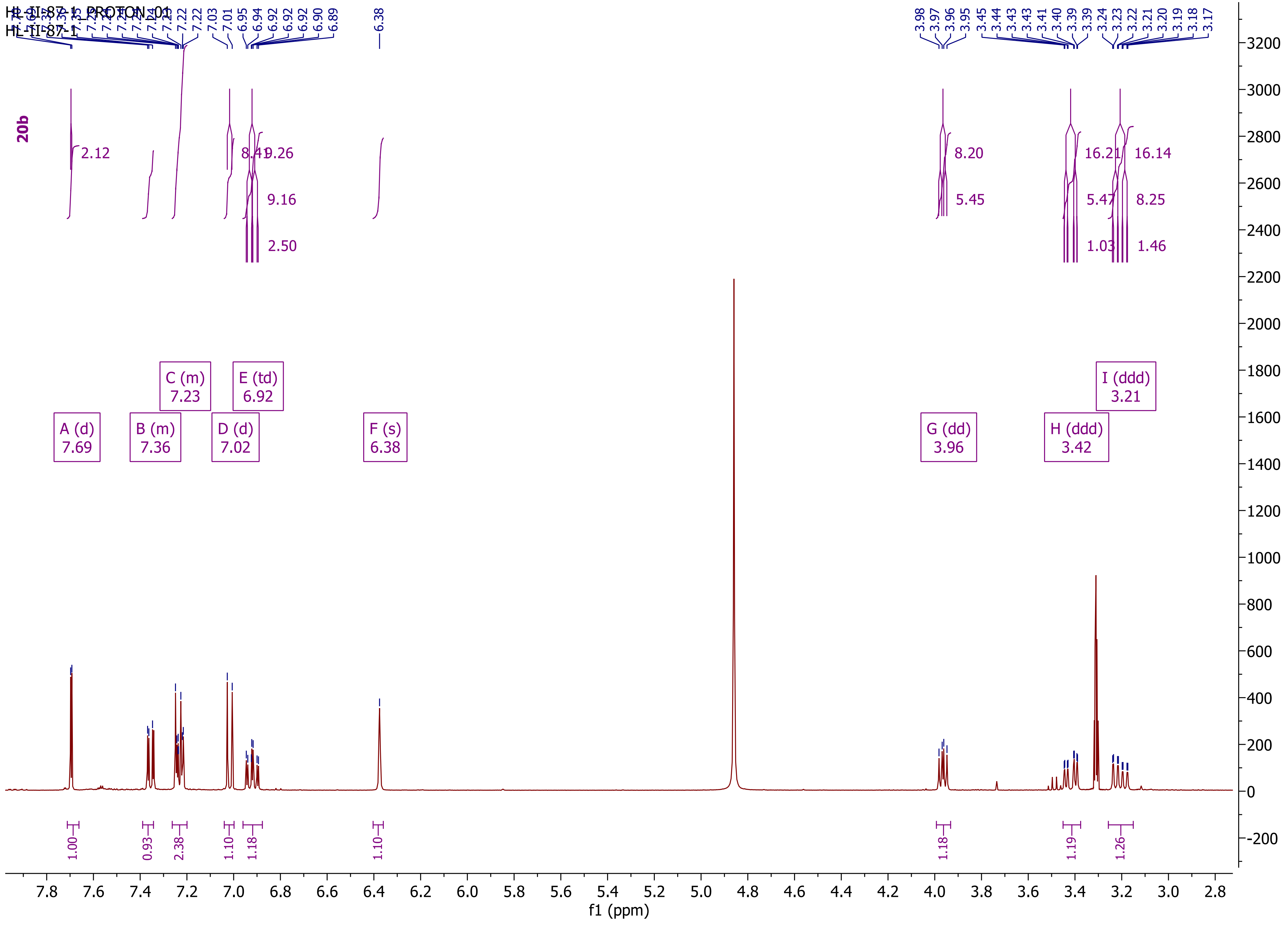

HL-II-87-1_PROTON_01
HL-II-87-1
20b
A (d) 7.69
B (m) 7.36
C (m) 7.23
D (d) 7.02
E (td) 6.92
F (s) 6.38
G (dd) 3.96
H (ddd) 3.42
I (ddd) 3.21
f1 (ppm)

HL-II-87-1_FLUORINE_01
HL-II-87-1
^{19}F NMR (376 MHz, Methanol-d_4) δ -126.52 (td, J = 9.5, 4.3 Hz).

20b

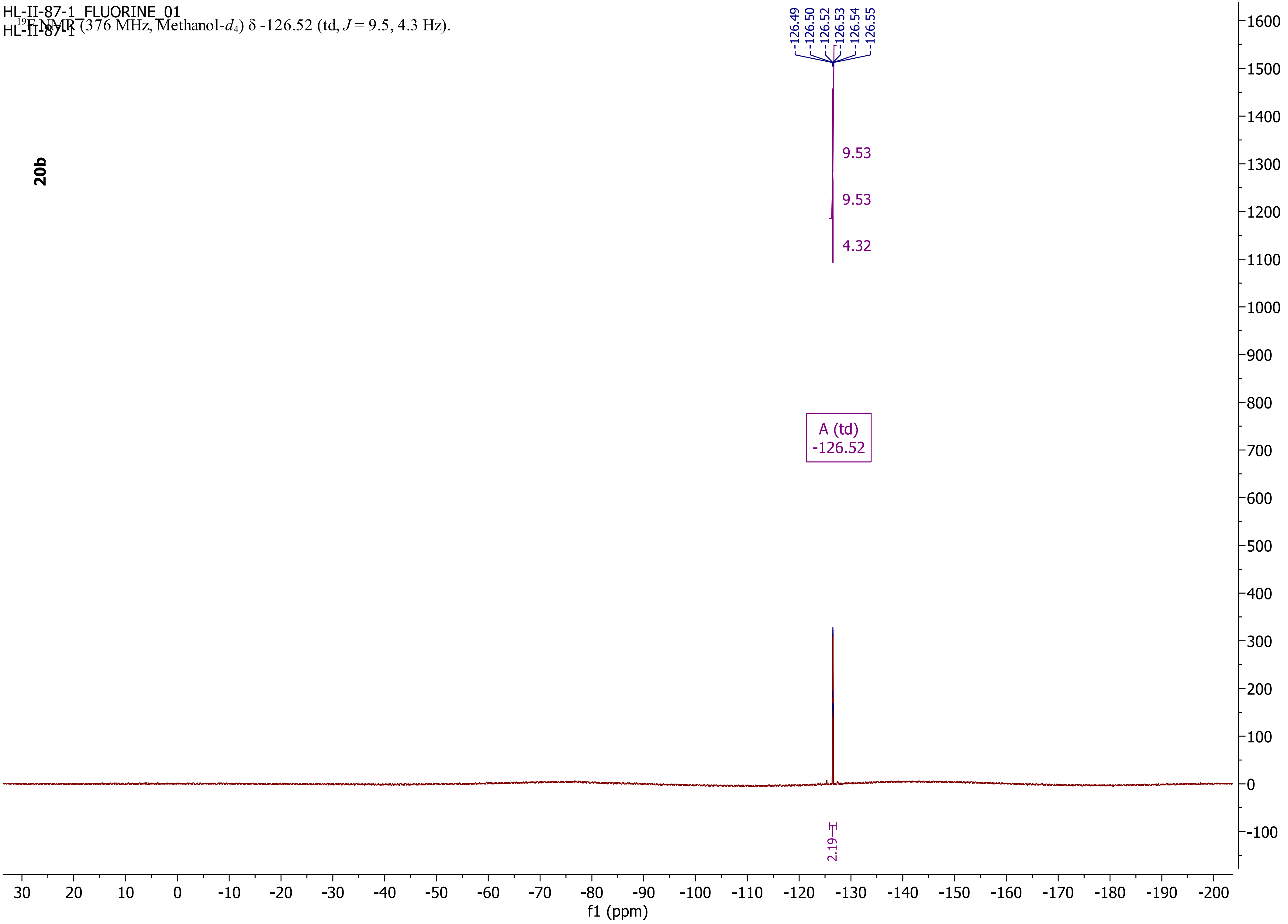

HL-II-87-1_CARBON_01

HL-II-87-1

^{13}C NMR (101 MHz, cd$_3$od) δ 173.56, 160.40, 158.07, 137.66, 137.18, 135.21, 133.57, 133.49, 131.05, 130.07, 129.07, 127.67, 127.57, 113.26, 113.17, 111.92, 111.65, 109.96, 109.91, 104.29, 104.05, 54.93, 52.51, 23.95.

20b

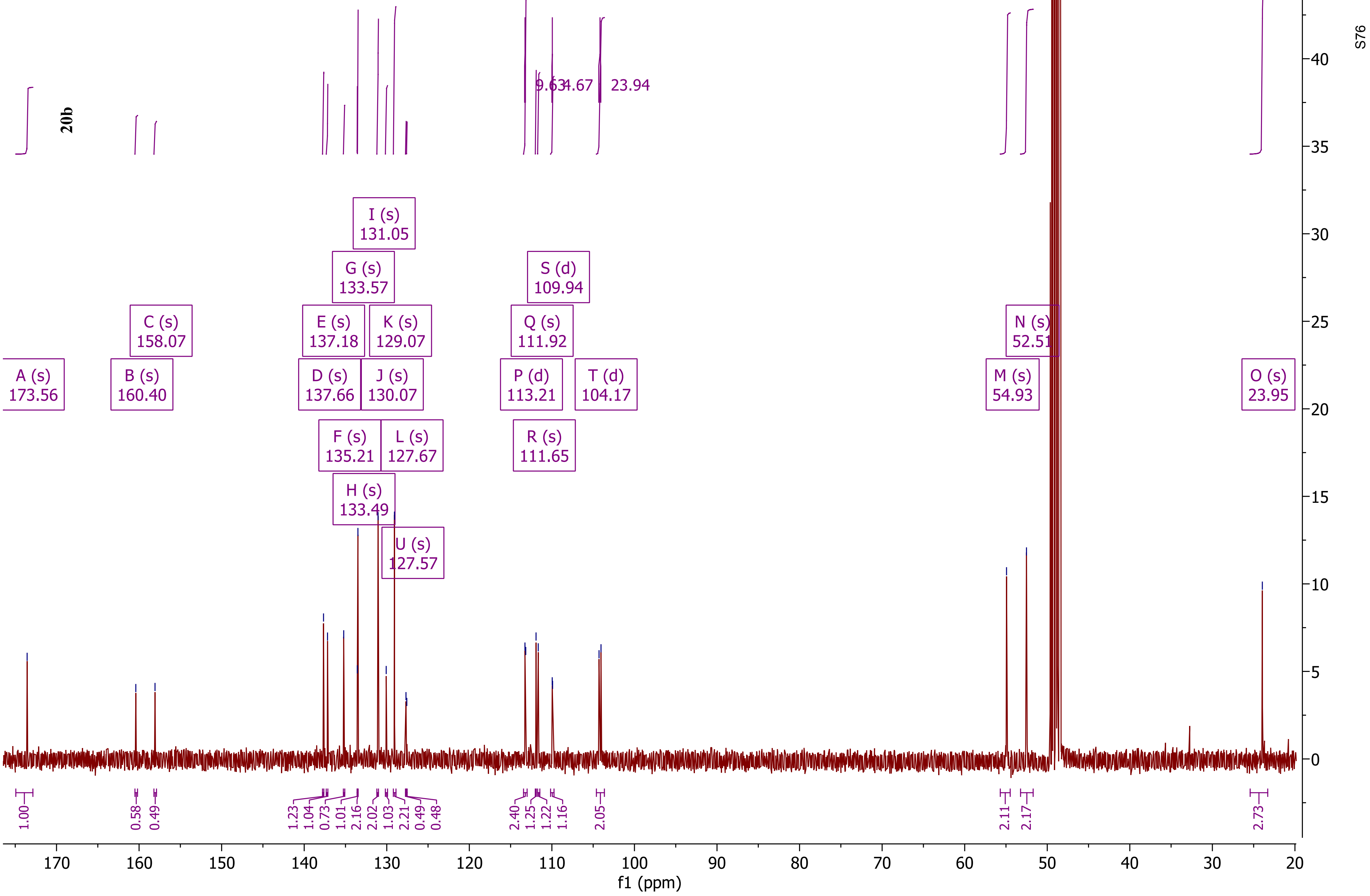

HL-II-87-1 CARBON_01

^{13}C NMR (101 MHz, cd$_3$od) δ 173.56, 160.40, 158.07, 137.66, 137.18, 135.21, 133.57, 133.49, 131.05, 130.07, 129.07, 127.67, 127.57, 113.26, 113.17, 111.92, 111.65, 109.96, 109.91, 104.29, 104.05, 54.93, 52.51, 23.95.

20b

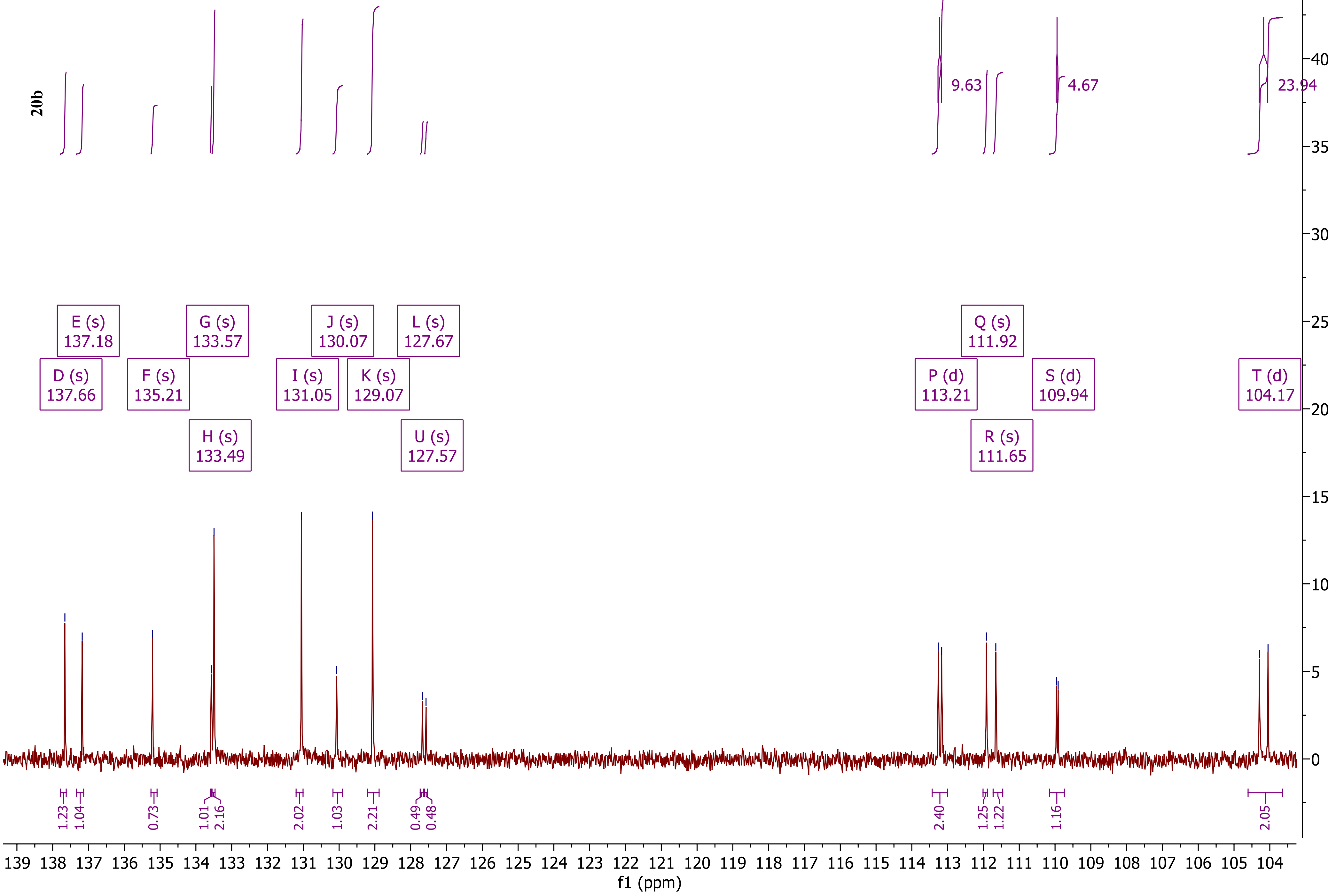

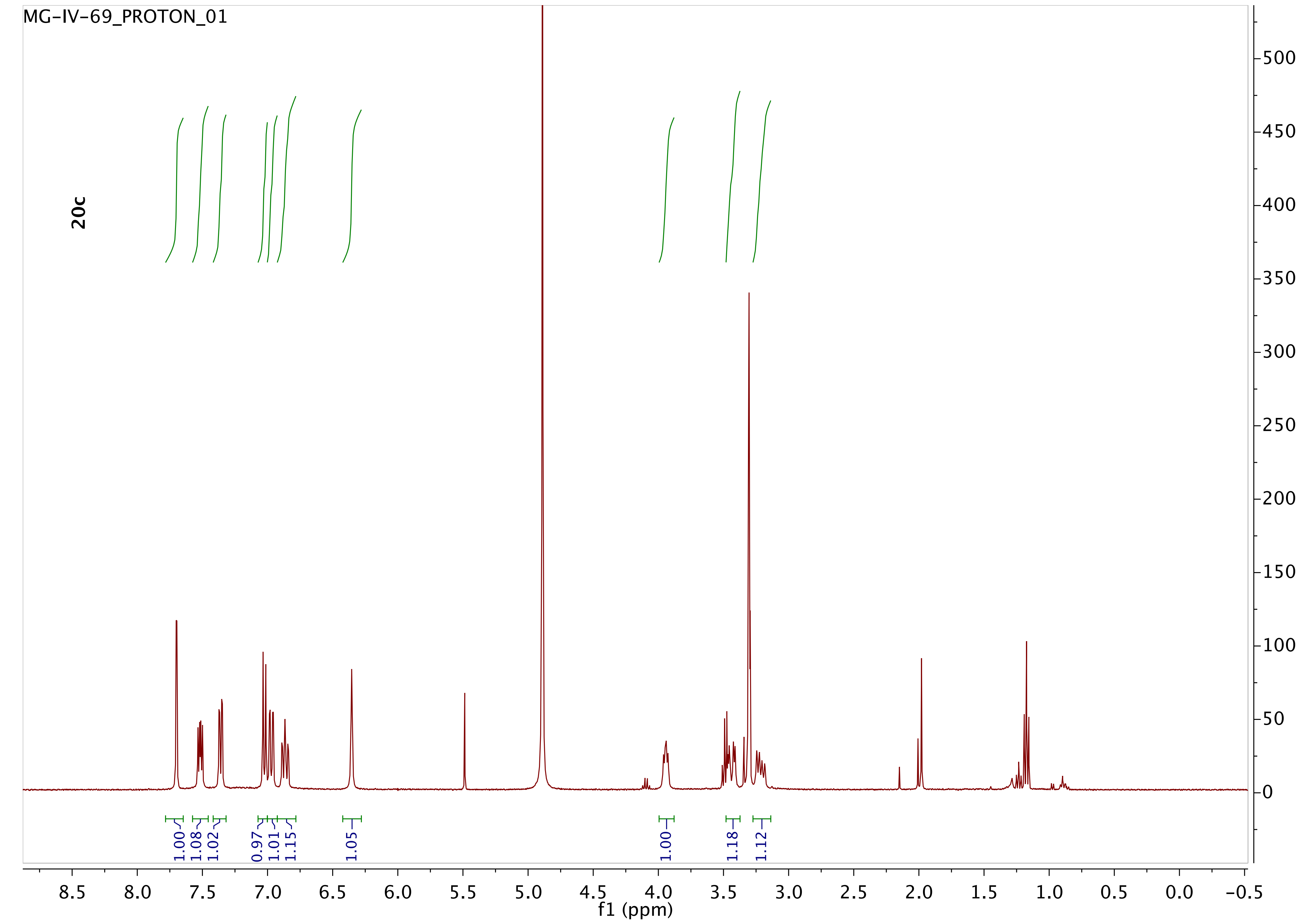
MG-IV-69_PROTON_01
20c
1.00
1.08
1.02
0.97
1.01
1.15
1.05
1.00
1.18
1.12
8.5 8.0 7.5 7.0 6.5 6.0 5.5 5.0 4.5 4.0 3.5 3.0 2.5 2.0 1.5 1.0 0.5 0.0 -0.5
f1 (ppm)
500 450 400 350 300 250 200 150 100 50 0

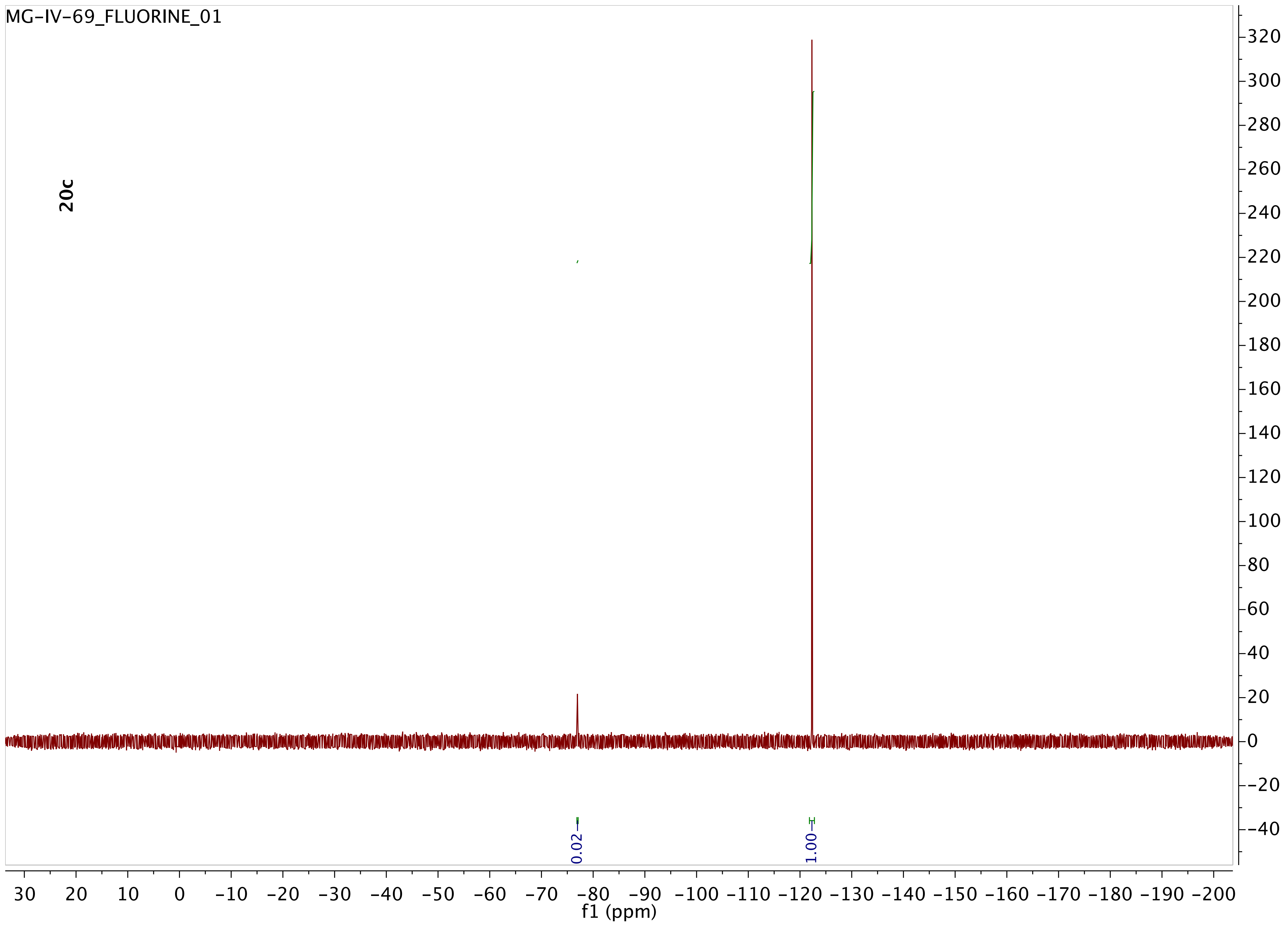

MG-IV-69_FLUORINE_01
20c
0.02
1.00
30 20 10 0 −10 −20 −30 −40 −50 −60 −70 −80 −90 −100 −110 −120 −130 −140 −150 −160 −170 −180 −190 −200
f1 (ppm)
320 300 280 260 240 220 200 180 160 140 120 100 80 60 40 20 0 −20 −40

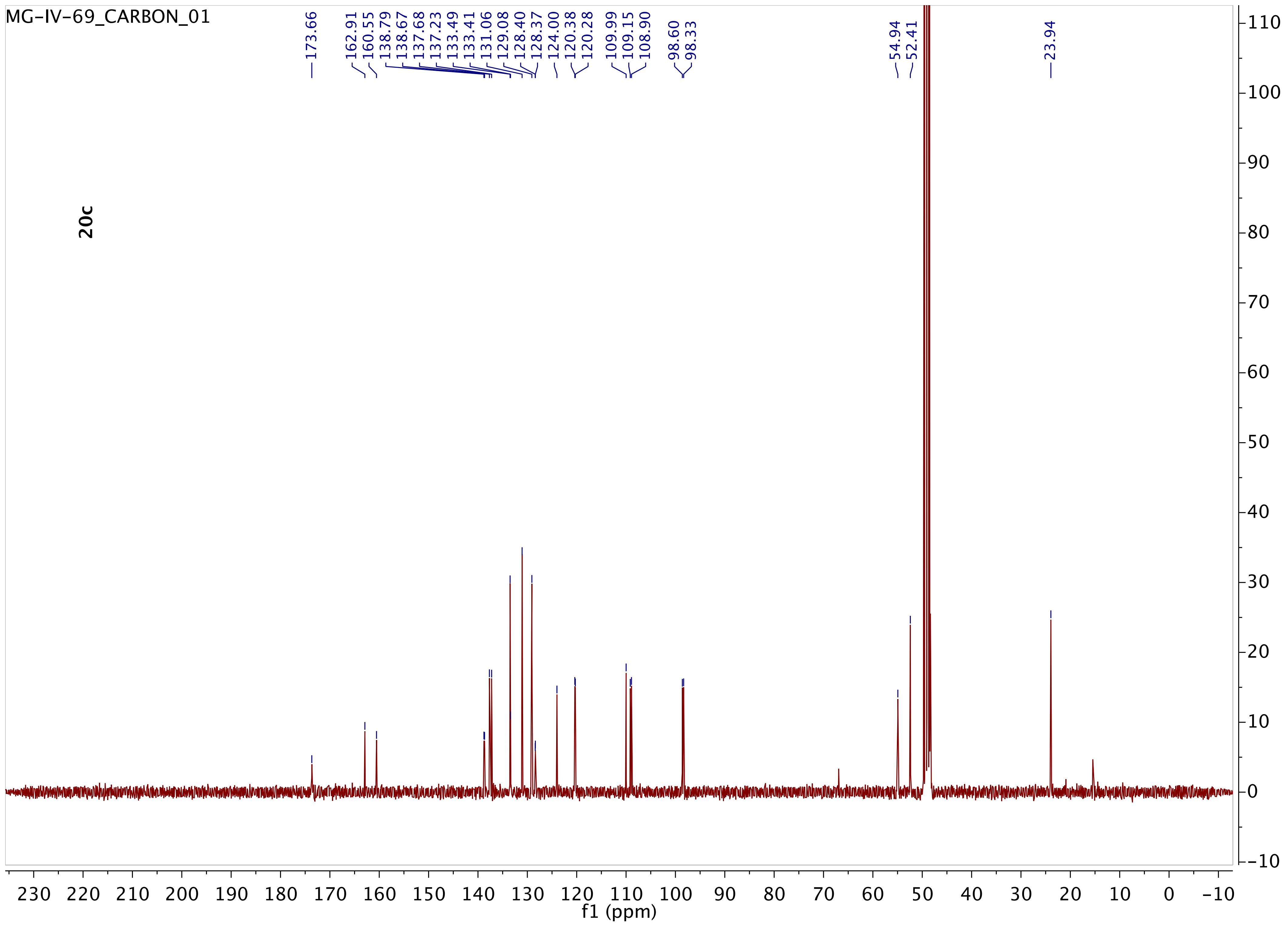
MG-IV-69_CARBON_01
20c
173.66
162.91
160.55
138.79
138.67
137.68
137.23
133.49
133.41
131.06
129.08
128.40
128.37
124.00
120.38
120.28
109.99
109.15
108.90
98.60
98.33
54.94
52.41
23.94
230 220 210 200 190 180 170 160 150 140 130 120 110 100 90 80 70 60 50 40 30 20 10 0 -10
f1 (ppm)
110 100 90 80 70 60 50 40 30 20 10 0 -10

S80

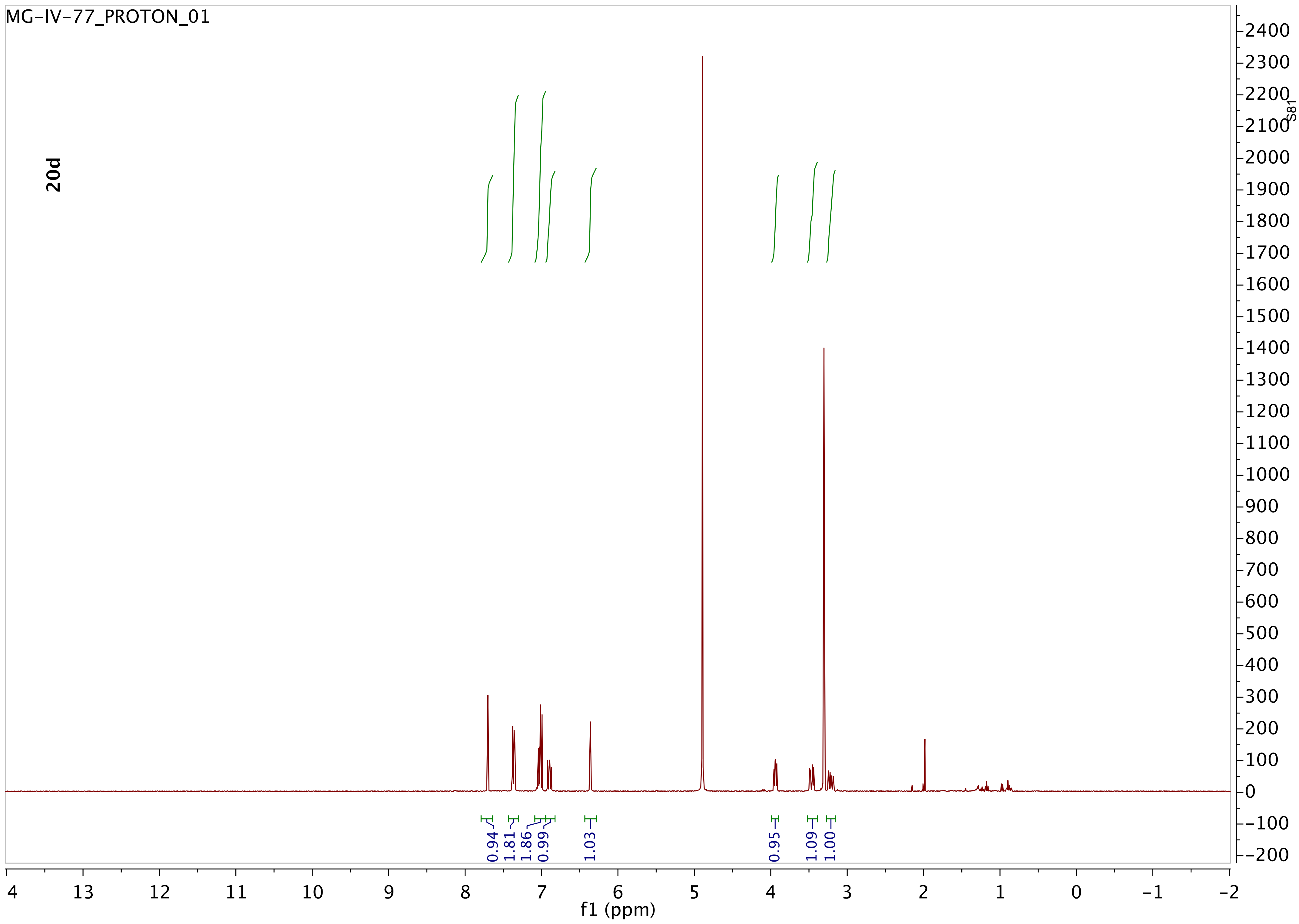
MG-IV-77_PROTON_01
20d
0.94
1.81
1.86
0.99
1.03
0.95
1.09
1.00
f1 (ppm)

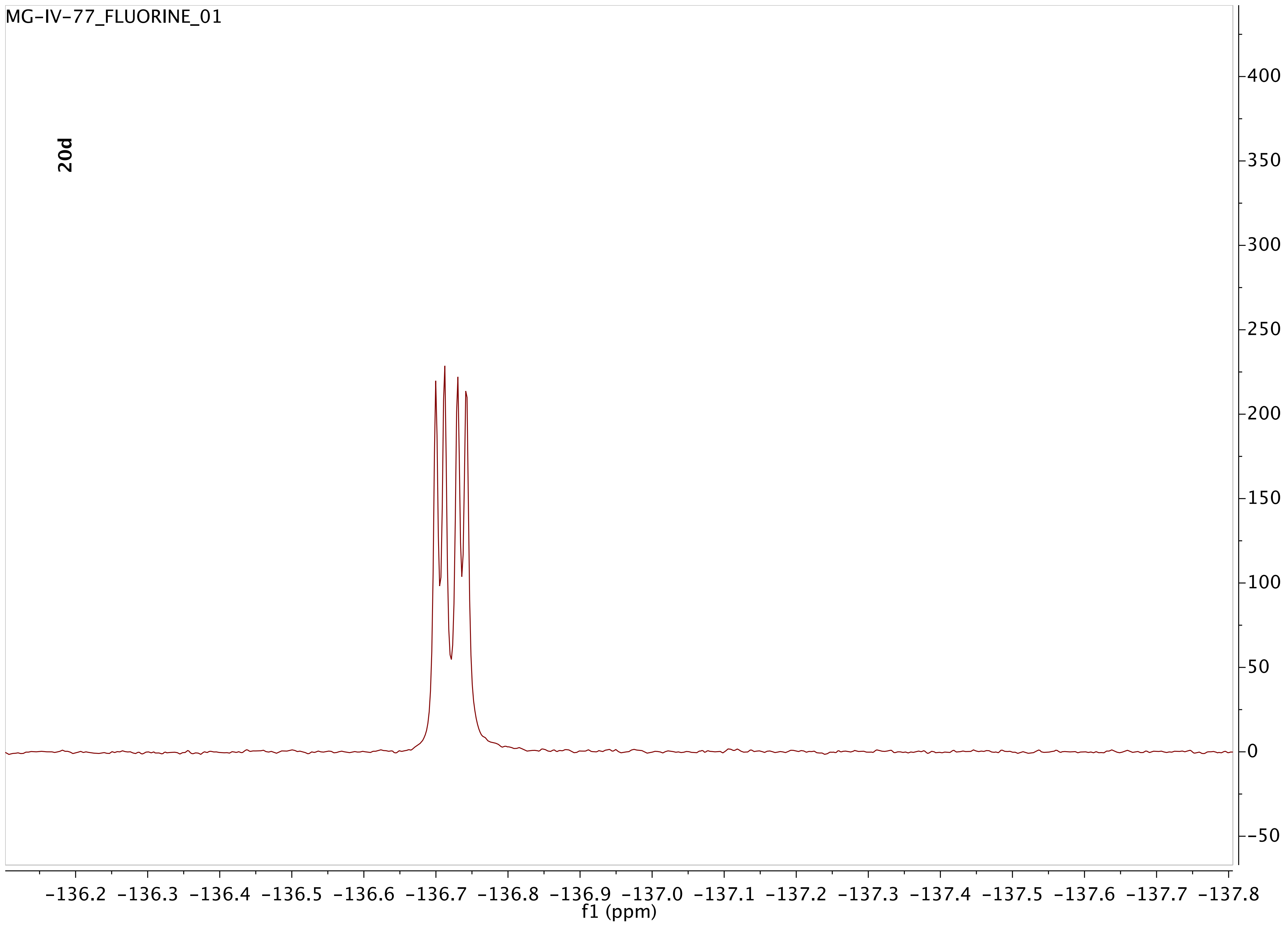
MG-IV-77_FLUORINE_01
20d
400
350
300
250
200
150
100
50
0
−50
−136.2 −136.3 −136.4 −136.5 −136.6 −136.7 −136.8 −136.9 −137.0 −137.1 −137.2 −137.3 −137.4 −137.5 −137.6 −137.7 −137.8
f1 (ppm)

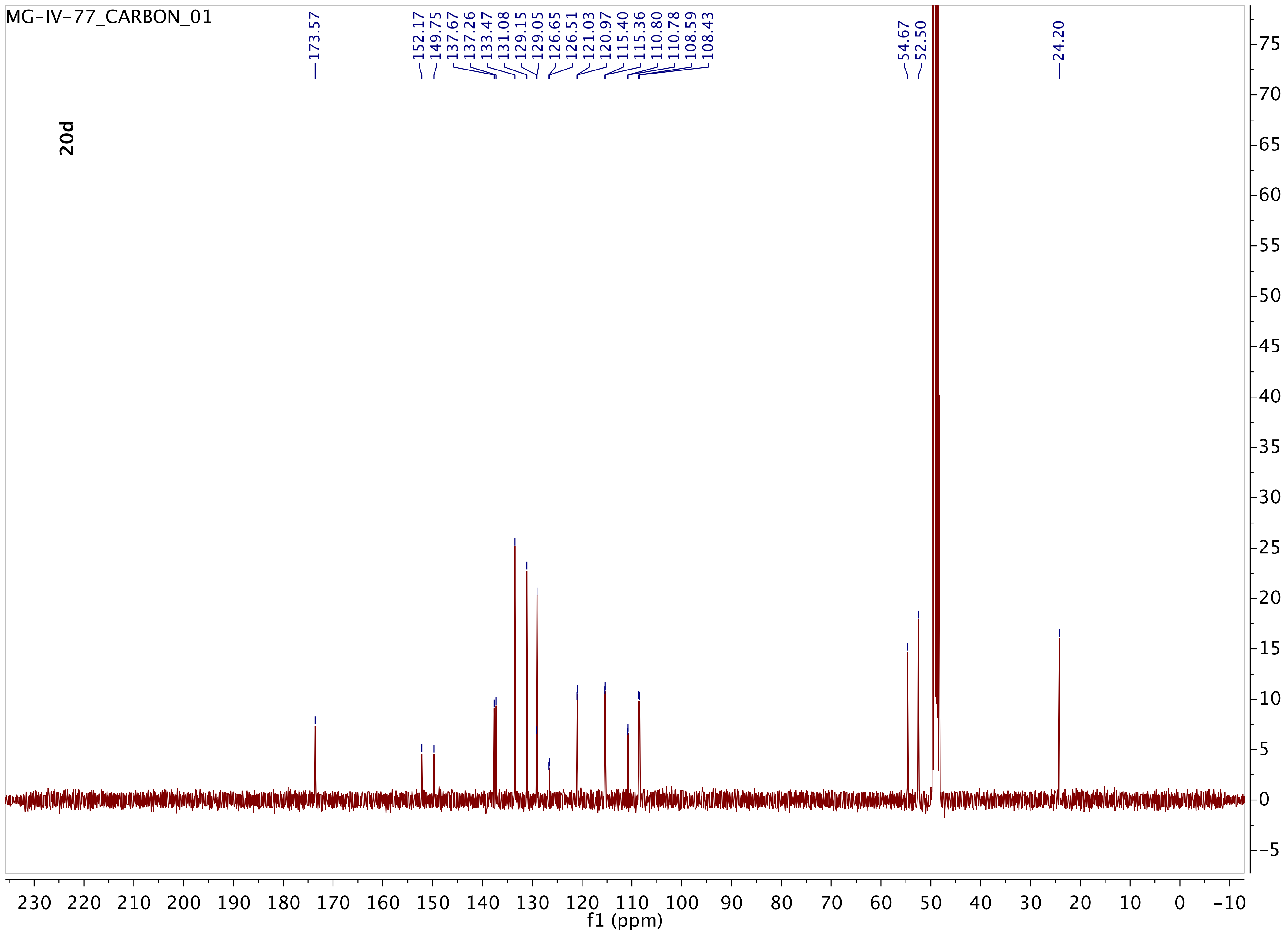

MG-IV-77_CARBON_01
20d
173.57
152.17
149.75
137.67
137.26
133.47
131.08
129.15
129.05
126.65
126.51
121.03
120.97
115.40
115.36
110.80
110.78
108.59
108.43
54.67
52.50
24.20
f1 (ppm)

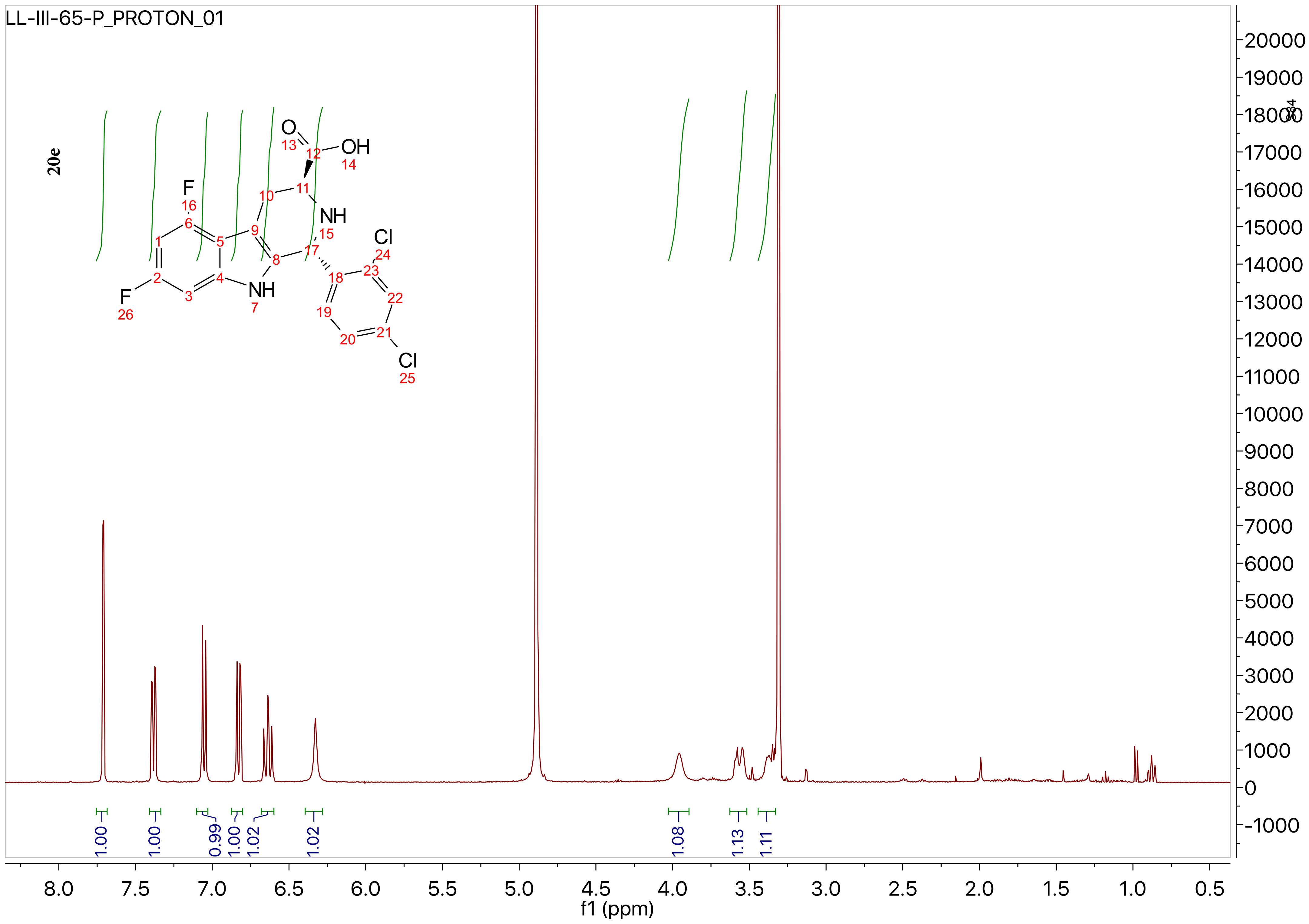

LL-III-65-P_PROTON_01
20e
F
16
6
1
5
9
10
11
12
O
13
OH
14
NH
15
17
8
2
F
26
3
4
NH
7
18
23
Cl
24
22
19
20
21
Cl
25
1.00
1.00
0.99
1.00
1.02
1.02
1.08
1.13
1.11
8.0
7.5
7.0
6.5
6.0
5.5
5.0
4.5
4.0
3.5
3.0
2.5
2.0
1.5
1.0
0.5
f1 (ppm)
20000
19000
18000
17000
16000
15000
14000
13000
12000
11000
10000
9000
8000
7000
6000
5000
4000
3000
2000
1000
0
-1000

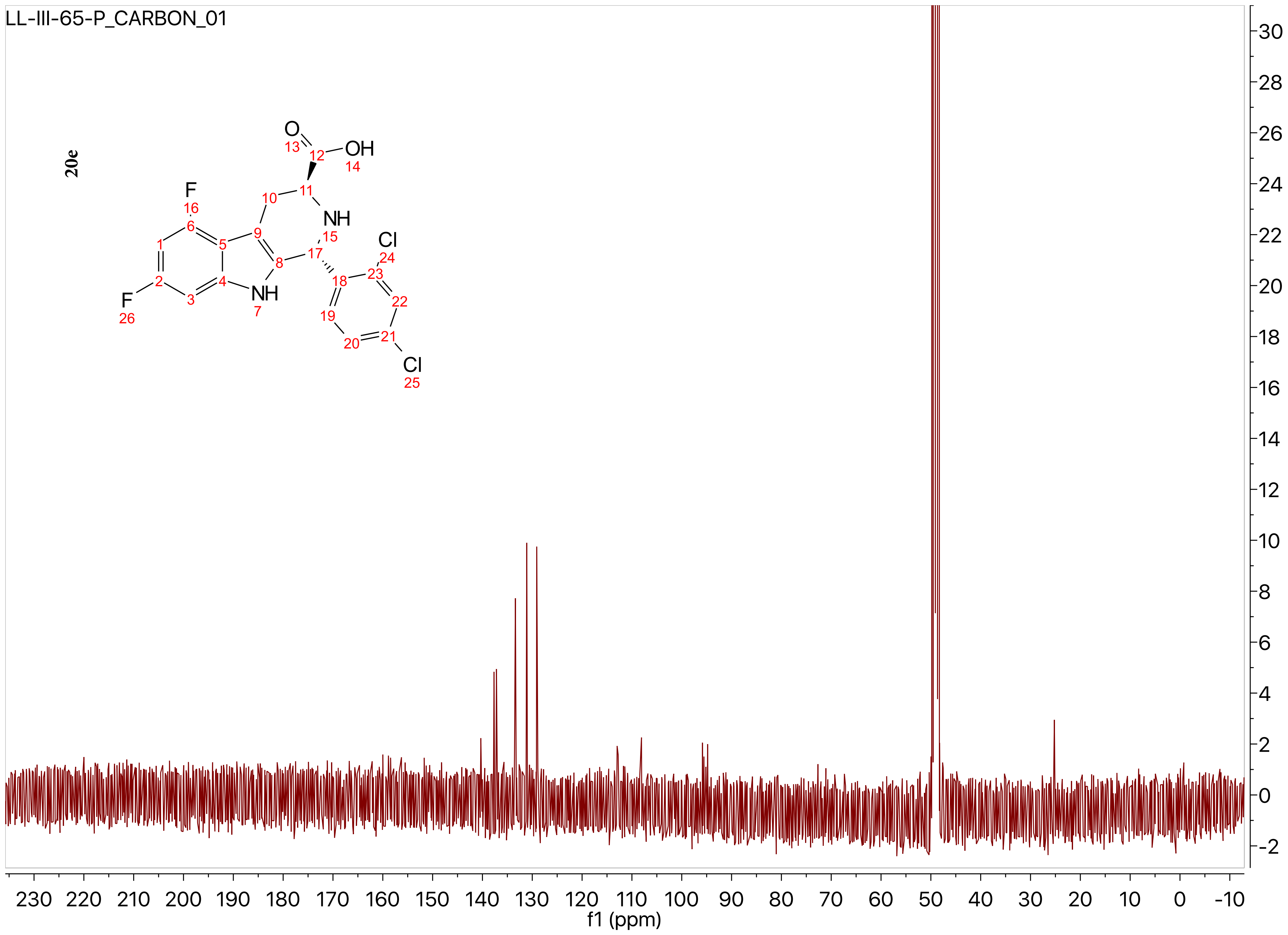
LL-III-65-P_CARBON_01
20e
f1 (ppm)

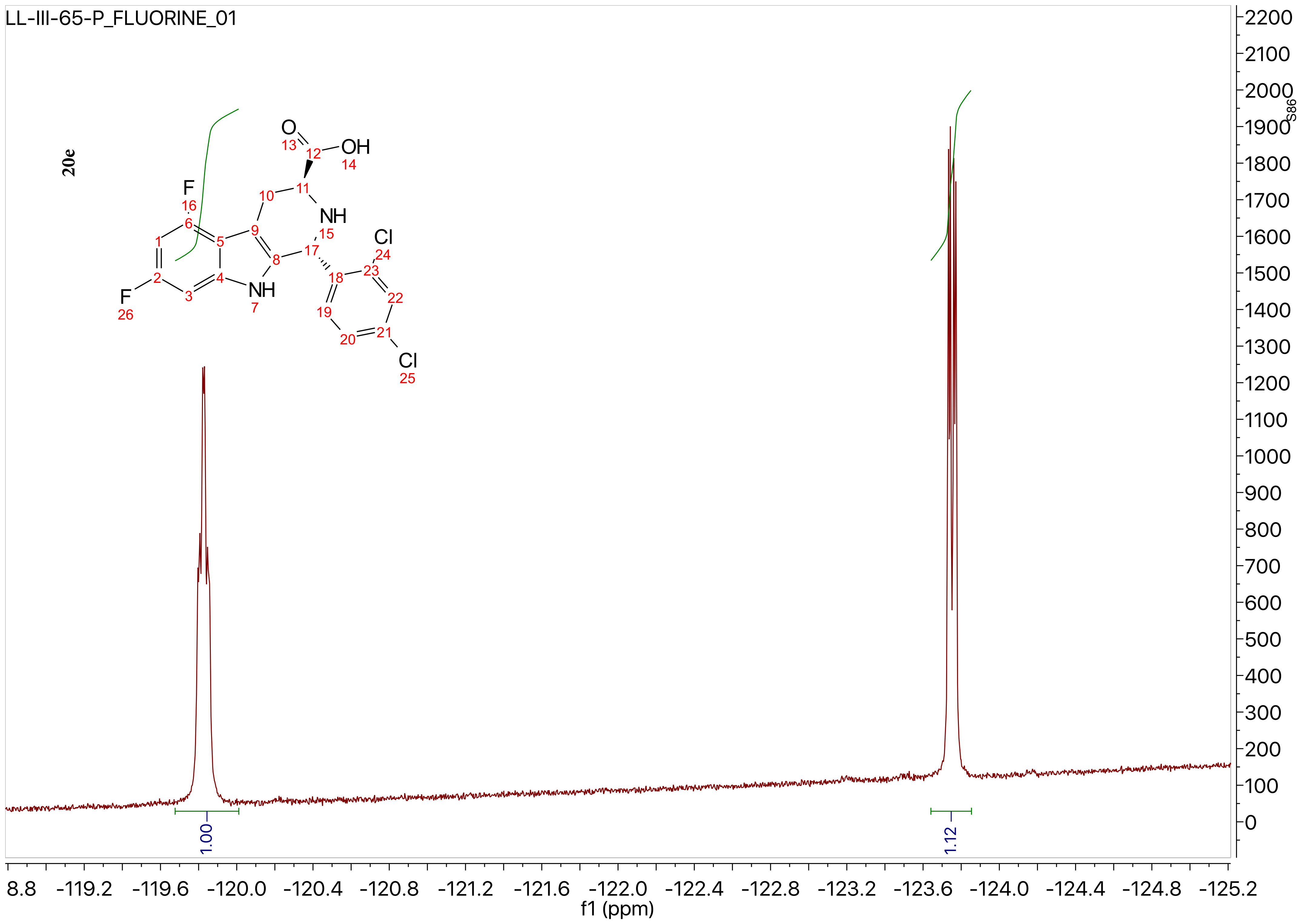
LL-III-65-P_FLUORINE_01
20e
F 16
F 26
NH 7
NH 15
O 13
OH 14
Cl 24
Cl 25
1.00
1.12
8.8 -119.2 -119.6 -120.0 -120.4 -120.8 -121.2 -121.6 -122.0 -122.4 -122.8 -123.2 -123.6 -124.0 -124.4 -124.8 -125.2
f1 (ppm)
2200 2100 2000 1900 1800 1700 1600 1500 1400 1300 1200 1100 1000 900 800 700 600 500 400 300 200 100 0

HL-I-177-1_PROTON_01

21a

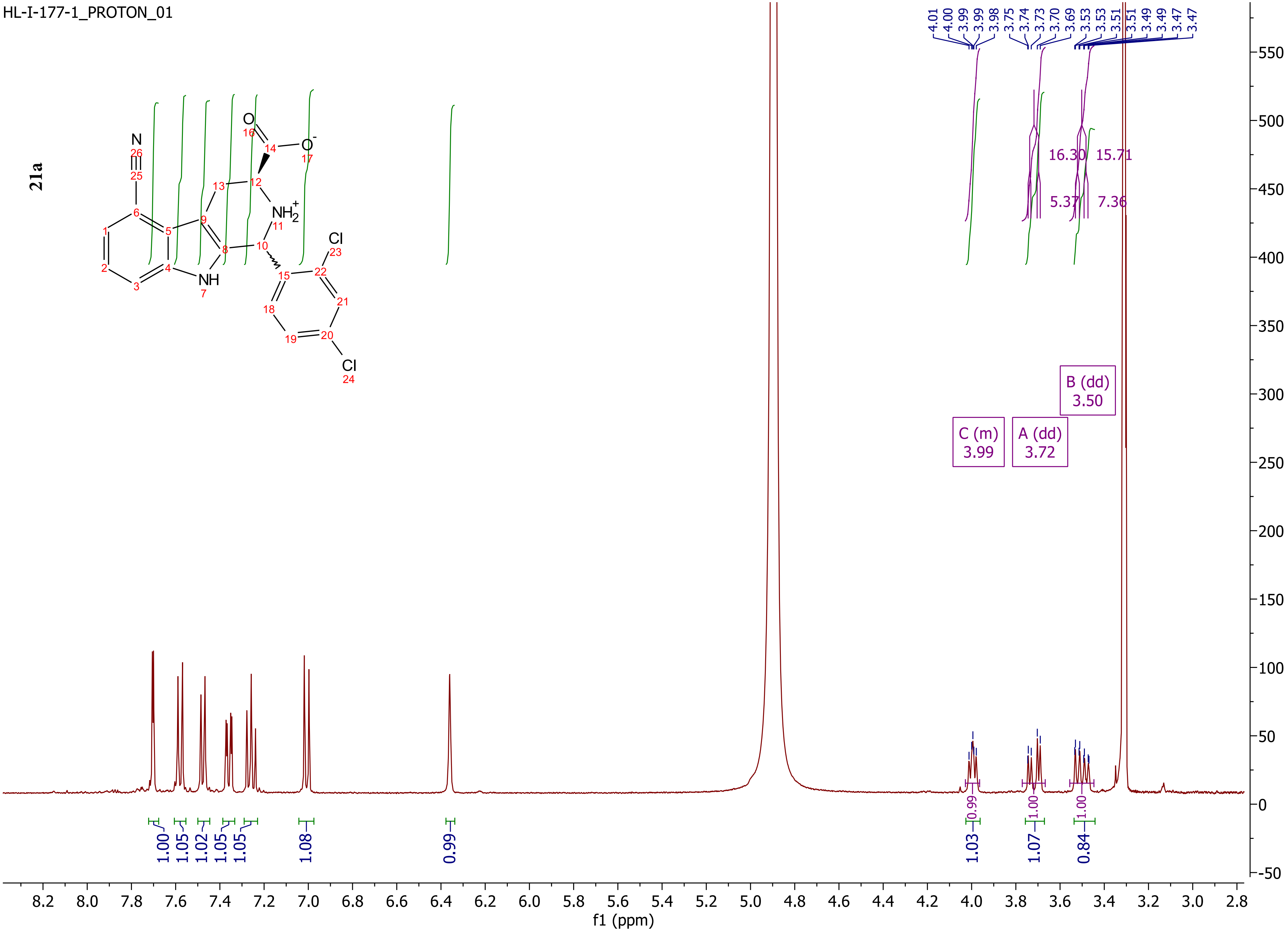

HL-IV-107-1.2.fid
13C
C13CPG_ICON MeOD /opt/topspin3.2/icon-data carrier-4 7

21a

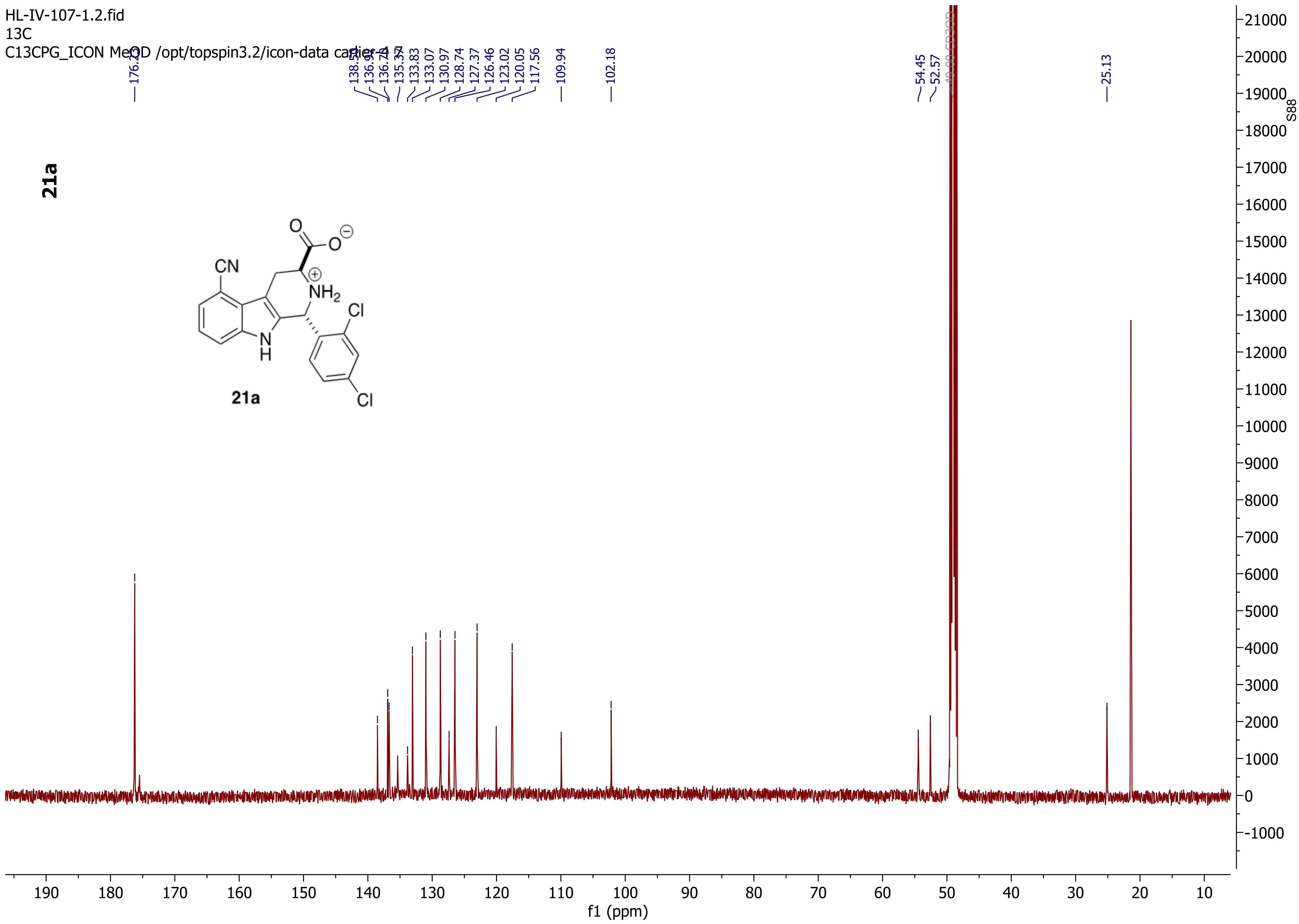

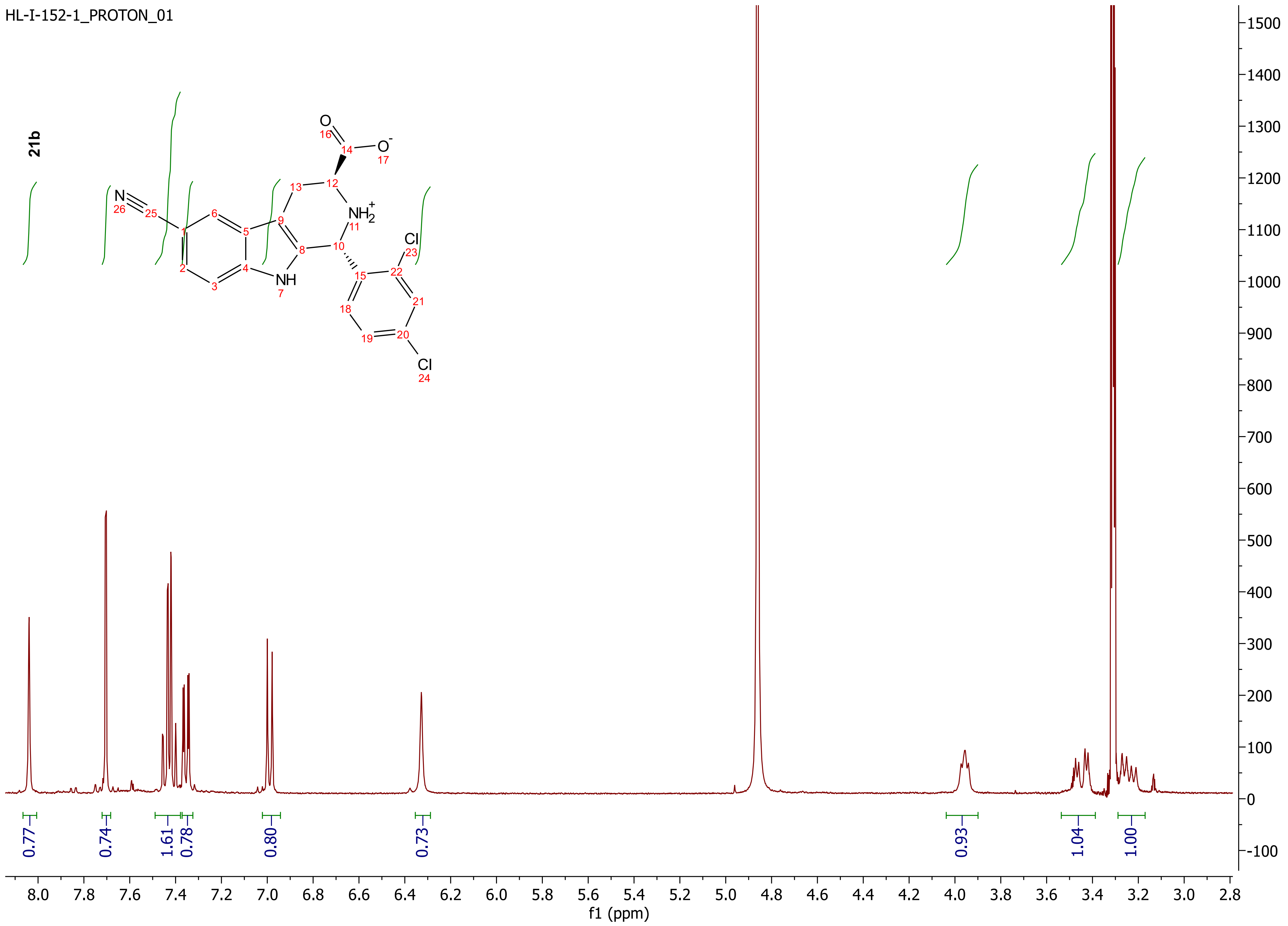
HL-I-152-1_PROTON_01
21b
N
26
25
1
2
3
4
5
6
NH
7
8
9
10
NH2+
11
12
13
14
O
16
O-
17
15
18
19
20
21
22
Cl
23
Cl
24
0.77
0.74
1.61
0.78
0.80
0.73
0.93
1.04
1.00
8.0
7.8
7.6
7.4
7.2
7.0
6.8
6.6
6.4
6.2
6.0
5.8
5.6
5.4
5.2
5.0
4.8
4.6
4.4
4.2
4.0
3.8
3.6
3.4
3.2
3.0
2.8
f1 (ppm)
1500
1400
1300
1200
1100
1000
900
800
700
600
500
400
300
200
100
0
-100

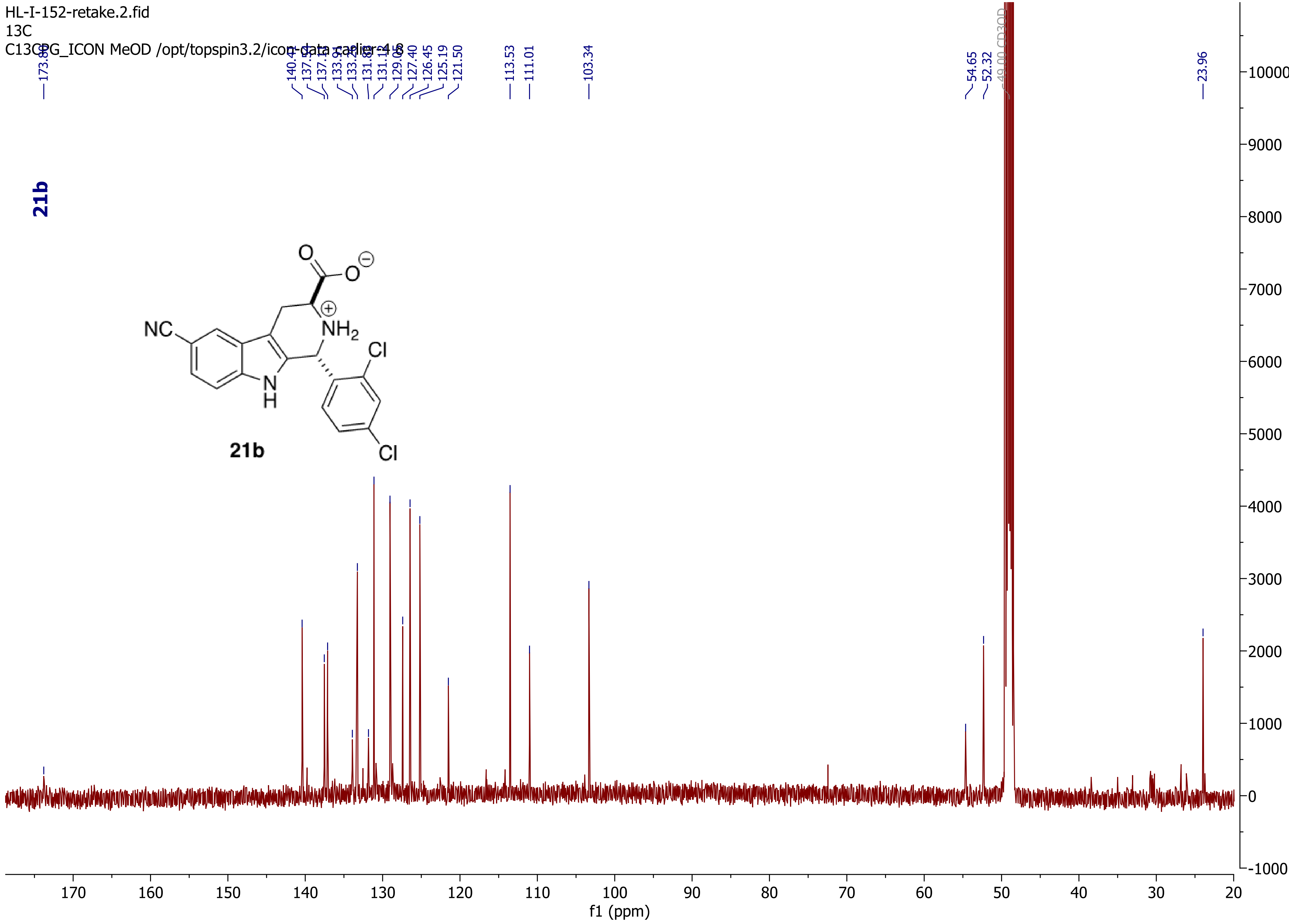

HL-I-152-retake.2.fid
13C
C13CPG_ICON MeOD /opt/topspin3.2/icon-data
21b
129.05
127.40
126.45
125.19
121.50
113.53
111.01
103.34
54.65
52.32
49.00 CD3OD
23.96
NC
O
NH2
N
H
Cl
Cl
f1 (ppm)

HL-I-127-1_PROTON_01

21c

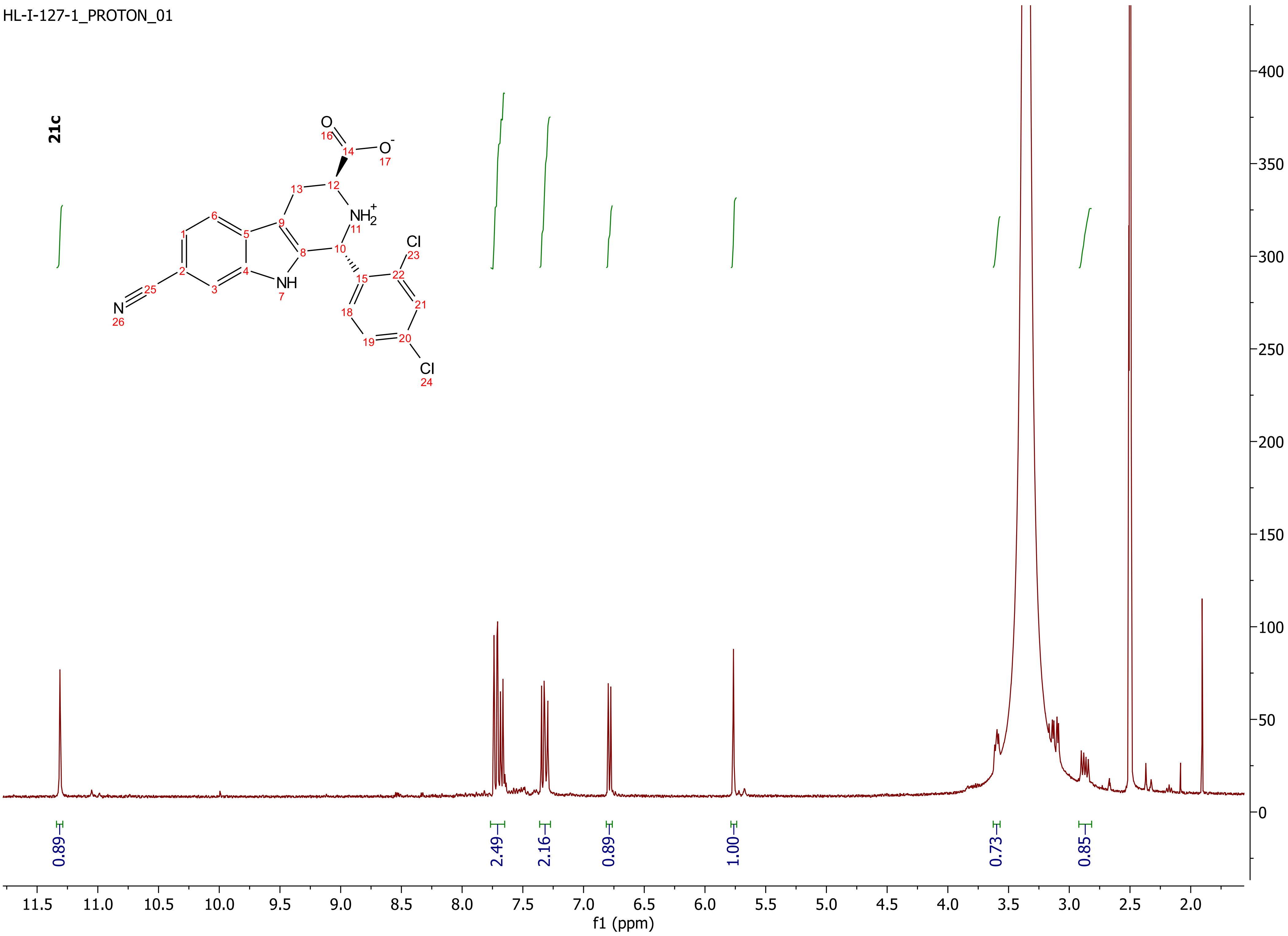

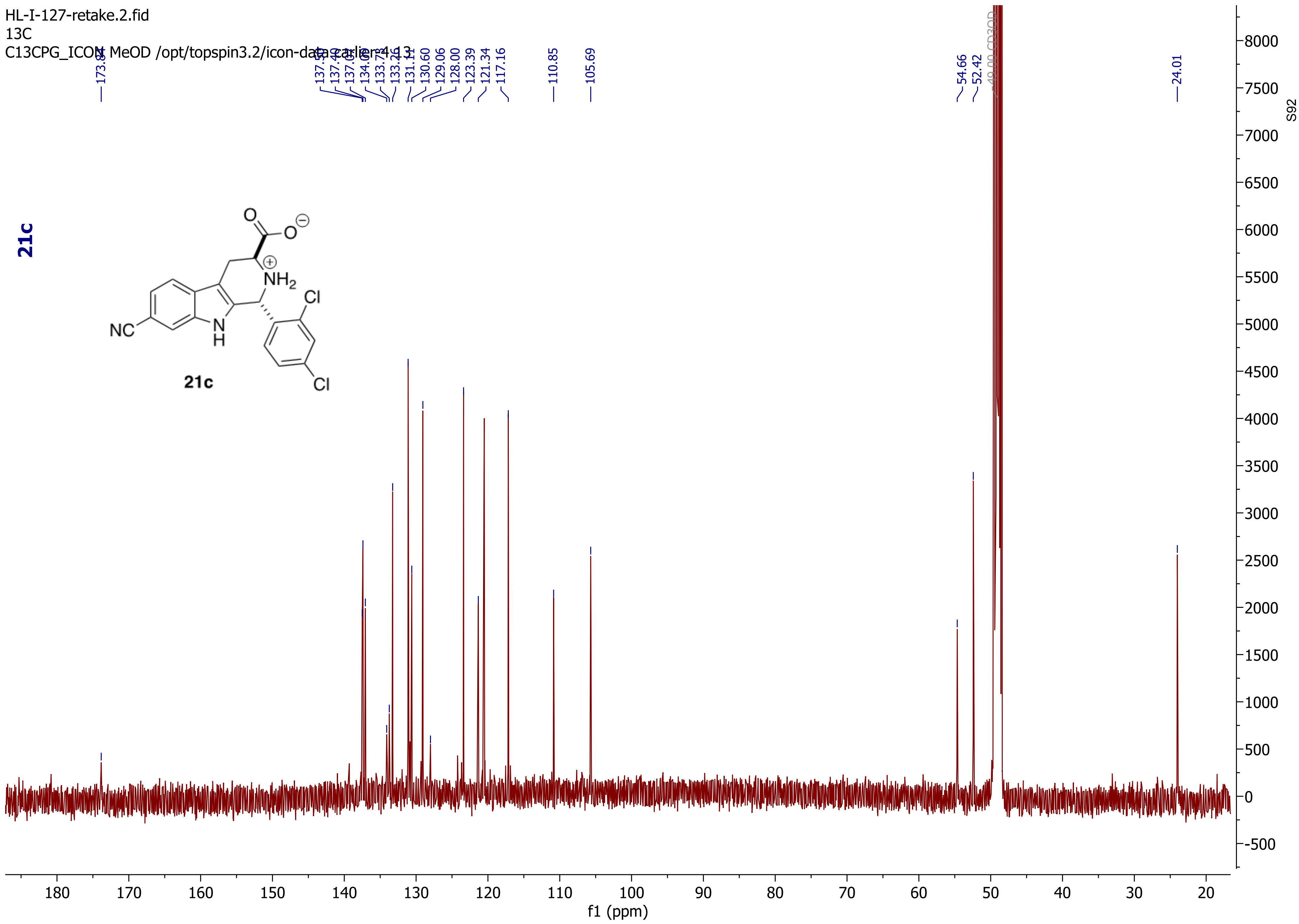
HL-I-127-retake.2.fid
13C
C13CPG_ICON MeOD /opt/topspin3.2/icon-data-earlier-4-13
21c
173.84
137.50
137.40
137.07
134.05
133.73
133.26
131.11
130.60
129.06
128.00
123.39
121.34
117.16
110.85
105.69
54.66
52.42
49.00 CD3OD
24.01
NC
N
H
NH2
O
O
Cl
Cl
21c
f1 (ppm)
